# Synthesis and Anti-Diabetic Evaluation of 2-Phenyl-3*H*-quinazolin-4-one Derivatives

**DOI:** 10.3390/pharmaceutics18081013

**Published:** 2026-08-16

**Authors:** K. P. D. H. Pathirana, D. A. Upeka Chathurangani, Julian Vlad, D. M. W. S. Dissanayake, Yasiru Vindula Alwis, G. Mashooda Jayah, K. P. S. S. Pathirana, Dinusha Nishani Udukala, Nishal M. Egodawaththa, Jason P. Farrah, Nasri Nesnas, Medha Jaimini Gunaratna

**Affiliations:** 1 College of Chemical Sciences, Institute of Chemistry Ceylon, Rajagiriya, Sri Jayawardenepura Kotte 10107, Sri Lanka; stu215821@student.ichemc.edu.lk (K.P.D.H.P.); upeka@ichemc.edu.lk (D.A.U.C.); warshadissanayake0@gmail.com (D.M.W.S.D.); vindulaalwis95@gmail.com (Y.V.A.); stu215775@student.ichemc.edu.lk (G.M.J.); dinusha@ichemc.edu.lk (D.N.U.); 2 Department of Chemistry and Chemical Engineering, Florida Institute of Technology, 150 West University Boulevard, Melbourne, FL 32901, USA; jvlad2024@my.fit.edu (J.V.); enishalmaduj2020@my.fit.edu (N.M.E.); nesnas@fit.edu (N.N.); 3 Department of Chemistry, Faculty of Science, University of Kelaniya, Kelaniya 11600, Sri Lanka; swapnapathirana@gmail.com; 4 Body Revolution Aesthetics & Wellness, 1211 48th Avenue, Myrtle Beach, SC 29577, USA; drfarrah@bodyrevolutionwellness.com

**Keywords:** 2-Phenyl-3*H*-quinazolin-4-one, Quinazolinone derivative, α-Amylase inhibition, α-Glucosidase inhibition, Antidiabetic activity

## Abstract

**Background/Objectives**: Diabetes mellitus is a common endocrine disorder characterized by hyperglycemia, which is increasing steadily all over the world. Current medications are limited due to lower efficacy and side effects; therefore, the introduction of novel compounds is crucial to improve therapeutic outcomes for diabetic patients. The objective of this study was to synthesize and evaluate 2-phenyl-3*H*-quinazolin-4-one and its derivatives for the glucose uptake across the yeast cell membranes, alpha-amylase inhibition, and alpha-glucosidase inhibition in silico and in vitro. **Methods**: The synthesis of 2-phenyl-3*H*-quinazolin-4-one and its derivatives was carried out by oxidative cyclocondensation of 2-aminobenzamide (anthranilamide) with various benzaldehydes in the presence of aqueous iron (III) chloride. The synthesized compounds were characterized by melting point determination and spectroscopic techniques, including FTIR, NMR and HRMS. **Results**: In the glucose uptake by yeast assay, compound **3f** had a lower 50% glucose uptake value of 24.02 ± 0.96 mM, compared with the standard drug metformin (25.99 ± 2.41 mM). The alpha-amylase inhibition bioassay showed that compound **3e** exhibited very potent inhibition, with an IC_50_ of 0.43 ± 0.05 mM, compared with the standard drug acarbose (0.53 ± 0.21 mM). Compound **3l** exhibited strong alpha-glucosidase inhibition, with an IC_50_ of 0.42 ± 0.10 mM, compared with the standard drug acarbose (0.29 ± 0.50 mM). **Coclusions**: These findings suggest the potential of synthesized 2-phenyl-3*H*-quinazolin-4-one derivatives, **3a**–**o**, as promising candidates for managing diabetes mellitus. However, further studies are required to validate their efficacy and determine their mechanisms of action.

## 1. Introduction

Diabetes mellitus (DM) is a chronic endocrine disorder characterized by hyperglycemia or elevated blood glucose levels. It is an emerging public health concern that affects every country and every age group worldwide. In 2015, it was estimated that approximately 415 million people worldwide were affected by diabetes, and it is expected to exceed 640 million by the year 2040 [1]. Type 1 DM, also known as insulin-dependent diabetes mellitus, is characterized by the autoimmune destruction of insulin-secreting pancreatic β cells, leading to insulin deficiency [2,3]. Type 2 DM, also known as non-insulin-dependent diabetes mellitus, is the most common form of diabetes that results in 90–95% of cases [2]. In the early stages of the disease, body cells are resistant to the insulin action, even though insulin production is normal. The capacity of pancreatic β-cells to release insulin gradually diminishes as the glycemia level increases [2]. It is estimated that 439 million people will have type 2 DM by the year 2030, with most cases being in developing countries, where most patients are aged 45 to 64 years [4].

The mechanism of glucose transport across the cell membrane of *Saccharomyces cerevisiae* (baker’s yeast) has gained considerable attention as a valuable in vitro tool for screening the hyperglycemic potential of synthetic compounds and medicinal plant extracts [5]. The yeast cell model is simple, cost-effective, and physiologically relevant for investigating glucose transport mechanisms. This is due to the presence of transporter systems that have close similarities to those in mammalian cells. Glucose uptake in human tissues, particularly skeletal muscle, liver, and adipose tissue, is mediated by glucose transporters (GLUTs) through facilitated diffusion [6]. Under-expression of these glucose transporters can result in elevated blood glucose levels and associated complications. Therefore, targeting GLUTs has emerged as a promising therapeutic approach in managing type 2 DM. *S. cerevisiae* utilizes glucose-like mammalian cells, converting glucose into energy, ethanol, and carbon dioxide [6,7]. Yeast cells also take up glucose primarily through facilitated diffusion, utilizing their own hexose transporters, which are functionally analogous to mammalian GLUTs [8]. Recent studies have shown that *Saccharomyces pastorianus* extracts enhance GLUT4 translocation and activate PI3K/AKT signaling in mammalian cells [9]. *Saccharomyces boulardii* reduces glycemia and improves organ function in diabetic models [10]. Despite lacking insulin-mediated regulation, yeast remains a reliable preliminary screening system, with findings requiring validation in mammalian models.

The alpha-amylase enzyme is an important digestive enzyme that hydrolyzes complex carbohydrates such as starch. It catalyzes the hydrolysis of internal alpha-1,4-glycosidic linkages in starch to generate low-molecular-weight products, such as glucose and maltose units [11]. This process directly contributes to the elevated blood glucose levels. The rate of glucose production can be reduced by inhibiting the alpha-amylase-mediated breakdown of starch, thereby helping to control postprandial hyperglycemia, a key therapeutic target in type 2 DM.

Alpha-glucosidase is a hydrolase enzyme present in the brush border of intestinal cells that facilitates the absorption of glucose in the small intestine. This is done by catalyzing the hydrolytic cleavage of oligosaccharides into absorbable monosaccharides. Alpha-glucosidase inhibitors slow the digestion of oligosaccharides into monosaccharides in the intestine and thereby reduce glucose levels in the bloodstream, including postprandial hyperglycemia. Alpha-glucosidase inhibitors are utilized orally to treat type 2 DM [12].

Quinazolin-3*H*-quinazolin-4-one moiety has emerged as a robust and versatile scaffold in medicinal chemistry, serving as a core framework for the design of numerous pharmacologically active agents [13,14,15]. Unlike conventional α-glucosidase inhibitors such as acarbose and miglitol, which often cause gastrointestinal side effects due to excessive carbohydrate fermentation in the colon, recent studies on quinazolinone scaffolds have demonstrated promising alternatives with improved potency and tolerability [16]. The structural diversity of these scaffolds provides opportunities to reduce toxicity while maintaining efficacy. The bicyclic system, characterized by a fused benzene and pyrimidine ring with a carbonyl oxygen at the C-4 position, possesses unique physicochemical properties that facilitate high-affinity molecular recognition. These include a moderate dipole character, significant hydrogen-bonding capability through the amide (N3-H) and carbonyl (C=O) groups, and the potential for ion–dipole and cation-π interactions [17,18]. Furthermore, the planar, electron-rich aromatic system allows for effective π-π stacking and van der Waals interactions within the hydrophobic catalytic pockets of target enzymes, while the inherent rigidity of the framework ensures conformational stability during binding [19,20,21].

In the present study, a library of 2-phenyl-3*H*-quinazolin-4-one derivatives (**3a**-**3o**) was strategically designed to systematically explore the structure–activity relationship (SAR) arising from diverse electronic and steric modifications at the C-2 position. The quinazolinone scaffold confers a wide range of pharmaceutical properties, including antioxidant, anti-inflammatory, antimicrobial, antifungal, anticancer, and antidiabetic activities [21]. The structurally related quinazolinones have been reported as potential antidiabetic lead compounds as alpha-glucosidase inhibitors, providing the direct literature targeting carbohydrate hydrolysis enzymes. Quinazolinone–triazole hybrids exhibited significant α-glucosidase and α-amylase inhibition [22]. These previously synthesized 2-phenyl-3*H*-quinazolin-4-one derivatives with potent α-glucosidase inhibitory activity motivated our scaffold design [14]. Further, the quinazolinone ring system contains various functional groups including carbonyls, amines, and amides, which are necessary to interact with the targets of most clinically used antidiabetic agents (e.g., acarbose, metformin) [23]. Thus, the scaffold provides a synthetically accessible starting point for the development of dual α-amylase/α-glucosidase inhibitors with the potential to modulate cellular glucose uptake through structural variation at the 2-aryl position. A rapid, high-yielding synthetic route gives access to diverse 2-phenyl-substituted compounds for further drug exploration [24].

In 1869, Griess synthesized the first quinazoline derivative, 2-cyano-3,4-dihydro-4-oxoquinazoline, by reacting cyanogens with anthranilic acid [14,19]. Since then, various methods have been developed to synthesize quinazolinones using different catalysts and anthranilic acid derivatives. The synthesis of these derivatives was achieved via a one-pot oxidative cyclocondensation between 2-aminobenzamide (anthranilamide) and various substituted aryl aldehydes in the presence of iron (III) chloride (FeCl_3_) as a catalyst. This synthetic route was selected for its efficiency and environmental compatibility, utilizing FeCl_3_ as a mild oxidizing agent to facilitate ring closure under relatively benign conditions [24]. By incorporating a wide range of aryl substituents including electron-donating hydroxyl and methoxy groups as well as electron-withdrawing nitro and halogen moieties, thereby aiming to modulate the lipophilicity and binding orientation of the scaffold. This approach allows for a comprehensive evaluation of how specific 2-aryl substitutions influence the dual inhibition of α-amylase and α-glycosidase relative to the positive control, acarbose, alongside the facilitation of cellular glucose uptake, which is assessed against metformin [25,26,27].

Though there are many different antidiabetic medications such as metformin, acarbose, miglitol, and voglibose on the market today, many of them have serious drawbacks, such as gastrointestinal side effects, decreased effectiveness with prolonged use, and a lack of selectivity [23]. These disadvantages show the urgent need for new therapeutic agents. Therefore, this study aims to evaluate a library of 2-phenyl-3*H*-quinazolin-4-one derivatives for the dual inhibition of α-amylase and α-glucosidase, alongside their potential to modulate glucose uptake in yeast, thereby addressing the limitations of current antidiabetic therapies.

## 2. Materials and Methods

### 2.1. General

Chemicals and solvents were of analytical grade and purchased from Sigma-Aldrich (Saint Louis, MO, USA) and Sisco Research Laboratories Pvt. Ltd. (Mumbai, India). All chemical reactions were monitored with thin-layer chromatography (TLC) using Merck silica gel 60 F254 plates (Darmstadt, Germany). The melting ranges were determined by an open capillary method using the Afon^®^ DMP100 melting point device (Guangzhou, China). FT-IR spectra of the synthesized compounds were obtained from the ABB MB3000 FTIR spectrometer (Quebec City, QC, Canada). ^1^H NMR and ^13^C NMR spectra were recorded on a Bruker 400 MHz spectrometer (Billerica, MA, USA). Mass spectrometry data were analyzed using an Agilent 6230 spectrometer (Agilent Technologies, Santa Clara, CA, USA) or a JEOL AccuTOF DART-MS (JEOL USA, Peabody, MA, USA).

### 2.2. General Procedure for the Synthesis of 2-Phenyl-3H-quinazolin-4-one Derivatives, **3a**–**3o**

**Scheme 1:**
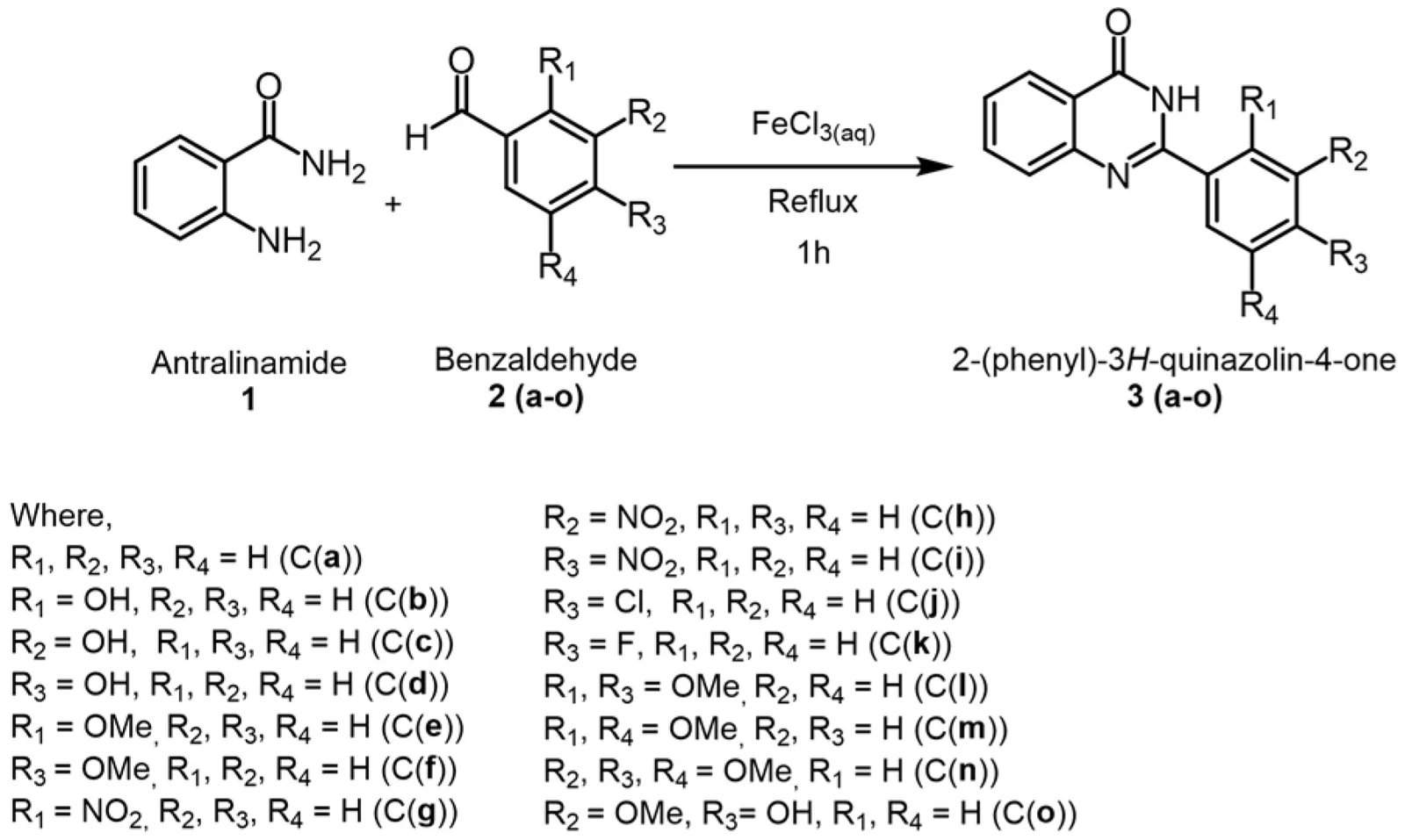
Synthesis of 2-(phenyl)-3*H*-quinazolin-4-one derivatives (**3a**–**3o**).

A mixture of anthranilamide (5 mmol, 680 mg) and the respective benzaldehyde (6 mmol) were refluxed in a solution of 0.2 mol dm^−3^ FeCl_3_ (10 mmol, 50.0 mL) in a 100 mL round-bottom flask immersed in a salt bath for 1 h at 100 °C (Scheme 1). After the reflux, a saturated solution of ethylenediaminetetraacetic acid (EDTA) (pH 4.65) (5.0 mL) was added and stirring continued for an additional 5 min. The precipitated crude product was collected by vacuum filtration and recrystallized using a mixed solvent system of DMF:water (*v*/*v* = 4:1). The solution was allowed to cool slowly to room temperature and then left to stand until crystallization was complete. The crystals were collected by vacuum filtration and dried to yield the series of quinazolinone derivatives (**3a**–**o**) in 65–96% yields.

### 2.3. Analysis of Physical and Spectroscopic Data of Synthesized Compounds

#### 2.3.1. 2-Phenyl-3*H*-quinazolin-4-one (**3a**)

Recrystallization solvent: mixed solvent system of DMF and water; white fiber-like, crystals (0.98 g, 88%); FTIR (cm^−1^): 3293.6–2847.7 (N-H stretching), 1660 (C=O stretching); ^1^H NMR (400 MHz, DMSO) δ 12.58 (s, 1H, NH), 8.29–8.14 (m, 3H, Ar-H), 7.88 (t, J = 7.7 Hz, 1H, Ar-H), 7.78 (d, J = 8.1 Hz, 1H, Ar-H), 7.67–7.51 (m, 4H, Ar-H); ^13^C NMR (101 MHz, DMSO) δ 162.86 (C=O), 152.97 (C=N), 149.42 (Ar-Cq), 135.23 (Ar-CH), 133.41 (Ar-Cq), 132.03 (Ar-CH), 129.26 (Ar-CH), 128.42 (Ar-CH), 128.17 (Ar-CH), 127.22 (Ar-CH), 126.51 (Ar-CH), 121.67 (Ar-Cq); HRMS *m*/*z* [M+H]^+^ calcd; C_14_H_10_N_2_O^+^, 223.2530, found; 223.0858. mp 237.2–237.8 °C.

#### 2.3.2. 2-(2-Hydroxyphenyl)-3*H*-quinazolin-4-one (**3b**)

Recrystallization solvent: mixed solvent system of DMF and water; white fiber-like crystalline solid (1.2 g, 70%); FTIR (cm^−1^): 3300–2900 (broad) (N-H stretching), 1658 (C=O stretching); ^1^H NMR (400 MHz, DMSO) δ 13.80 (s, 1H, NH), 12.54 (s, 1H, OH), 8.27 (dd, *J* = 8.1, 1.6 Hz, 1H, Ar-H), 8.20 (dd, *J* = 8.0, 1.5 Hz, 1H, Ar-H), 7.90 (ddd, *J* = 7.6, 7.1, 1.5 Hz, 1H, Ar-H), 7.81 (d, *J* = 8.1 Hz, 1H, Ar-H), 7.64–7.54 (m, 1H, Ar-H), 7.49 (ddd, *J* = 8.5, 7.1, 1.5 Hz, 1H, Ar-H), 7.05 (dd, *J* = 8.31, 1.10 Hz, 1H, Ar-H), 7.02–6.98 (m, 1H, Ar-H). ^13^C NMR (101 MHz, DMSO) δ 162.38 (C=O), 161.01 (Ar-C-OH), 152.42 (C=N), 148.66 (Ar-Cq), 135.94 (Ar-Cq), 134.65 (Ar-Cq), 128.64 (Ar-CH), 127.87 (Ar-CH), 126.97 (Ar-CH), 121.64 (Ar-CH), 119.74 (Ar-CH), 118.82 (Ar-CH), 114.68 (Ar-Cq); HRMS *m*/*z* [M+H]^+^ calcd; C_14_H_10_N_2_O_2_^+^, 239.2518, found 239.0807. mp 252.3–254.1 °C.

#### 2.3.3. 2-(3-Hydroxyphenyl)-3*H*-quinazolin-4-one (**3c**)

Recrystallization solvent: mixed solvent system of DMF and water; white fiber-like crystalline solid (0.59 g, 56%); FTIR (cm^−1^): 3443–3062 (N-H stretching), 1639 (C=O stretching); ^1^H NMR (400 MHz, DMSO) δ 12.46 (s, 1H, NH), 9.81 (s, 1H, OH), 8.19 (d, *J* = 8.0 Hz, 1H, Ar-H), 7.86 (t, *J* = 7.5 Hz, 1H, Ar-H), 7.76 (d, *J* = 8.2 Hz, 1H, Ar-H), 7.67–7.61 (m, 2H, Ar-H), 7.55 (t, *J* = 7.7 Hz, 1H, Ar-H), 7.38 (t, *J* = 7.9 Hz, 1H, Ar-H), 7.02 (d, *J* = 8.0 Hz, 1H, Ar-H); ^13^C NMR (101 MHz, DMSO) δ 162.21 (C=O), 157.50 (Ar-C-OH), 152.42 (C=N), 148.66 (Ar-Cq), 134.50 (Ar-CH), 134.06 (Ar-Cq), 129.61 (Ar-CH), 127.34 (Ar-CH), 126.45 (Ar-CH), 125.83 (Ar-CH), 120.98 (Ar-Cq), 118.49 (Ar-CH), 118.35 (Ar-CH), 114.60 (Ar-CH); HRMS *m*/*z* [M+H]^+^ calcd; C_14_H_10_N_2_O_2_^+^, 239.2518, found 239.0973. mp 270.8–272.3 °C.

#### 2.3.4. 2-(4-Hydroxyphenyl)-3*H*-quinazolin-4-one (**3d**)

Recrystallization solvent: mixed solvent system of DMF and water; off white needle-like crystalline solid (0.91 g, 76%); FTIR (cm^−1^): 3300–2500 (N-H and O-H stretching overlapped), 1654 (C=O stretching); ^1^H NMR (400 MHz, DMSO) δ 12.32 (s, 1H, NH), 10.17 (s, 1H, OH), 8.17–8.11 (m, 3H, Ar-H), 7.84 (t, *J* = 7.7 Hz, 1H, Ar-H), 7.71 (d, *J* = 8.2 Hz, 1H, Ar-H), 7.50 (t, *J* = 7.6 Hz, 1H, Ar-H), 6.96–6.87 (m, 2H, Ar-H); ^13^C NMR (101 MHz, DMSO) δ 163.26 (C=O), 161.46 (Ar-C-OH), 153.09 (C=N), 149.86 (Ar-Cq), 135.31 (Ar-CH), 130.45 (Ar-CH), 127.96 (Ar-CH), 126.69 (Ar-CH), 124.12 (Ar-Cq), 121.47 (Ar-Cq), 116.24 (Ar-CH); HRMS *m*/*z* [M+H]+ calcd; C_14_H_10_N_2_O_2_^+^, 239.2518, found 239.0971. mp 310.8–312.3 °C.

#### 2.3.5. 2-(2-Methoxyphenyl)-3*H*-quinazolin-4-one (**3e**)

Recrystallization solvent: mixed solvent system of DMF and water; white needle-like crystalline solid (0.85 g, 78.2%); FTIR (cm^−1^): 3500–3100 (N-H stretching), 1650 (C=O stretching); ^1^H NMR (400 MHz, DMSO) δ 12.10 (s, 1H, NH), 8.16 (dd, *J* = 8.0, 1.6 Hz, 1H, Ar-H), 7.89–7.80 (m, 1H, Ar-H), 7.77–7.73 (m, 2H, Ar-H), 7.60–7.54 (m, 2H, Ar-H), 7.23 (d, *J* = 8.4 Hz, 1H, Ar-H), 7.13 (t, J = 7.5 Hz, 1H, Ar-H), 3.87 (s, 3H, OCH_3_). ^13^C NMR (101 MHz, DMSO) δ 162.16 (C=O), 158.07 (Ar-C-OCH_3_), 153.29 (C=N), 149.94 (Ar-Cq), 135.32 (Ar-CH), 133.13 (Ar-CH), 131.37 (Ar-CH), 128.29 (Ar-CH), 127.46 (Ar-CH), 126.70 (Ar-CH), 123.58 (Ar-Cq), 121.92 (Ar-Cq), 121.36 (Ar-CH), 112.80 (Ar-CH), 56.70 (CH_3_-O); HRMS *m*/*z* [M+H]^+^ calcd; C_15_H_12_N_2_O_2_^+^, 253.0977, found 253.1247. mp 203.9–206.7 °C.

#### 2.3.6. 2-(4-Methoxyphenyl)-3*H*-quinazolin-4-one (**3f**)

Recrystallization solvent: mixed solvent system of DMF and water; off white needle-like crystalline solid (1.1 g, 88%); FTIR (cm^−1^): 3300–2800 (N-H stretching), 1654 (C=O stretching); ^1^H NMR (400 MHz, DMSO) δ 12.43 (s, 1H, NH), 8.28–8.14 (m, 3H, Ar-H), 7.89–7.68 (m, 2H, Ar-H), 7.52 (t, *J* = 7.4 Hz, 1H, Ar-H), 7.13 (d, *J* = 8.8 Hz, 2H, Ar-H), 3.89 (s, 3H, OCH_3_); ^13^C NMR (101 MHz, DMSO) δ 163.38 (C=O), 162.96 (Ar-C-OCH_3_), 152.98 (C=N), 149.99 (Ar-Cq), 135.55 (Ar-CH), 130.52 (Ar-CH), 128.31 (Ar-CH), 127.15 (Ar-CH), 126.89 (Ar-CH), 125.92 (Ar-Cq), 121.76 (Ar-Cq), 115.07, 56.53 (CH_3_-O); HRMS *m*/*z* [M+H]^+^ calcd; C_15_H_12_N_2_O_2_^+^, 253.0977, found 253.1138. mp 247.5–249.9 °C.

#### 2.3.7. 2-(2-Nitrophenyl)-3*H*-quinazolin-4-one (**3g**)

Recrystallization solvent: mixed solvent system of DMF and water; off white needle-like crystalline solid (0.74 g, 55%) FTIR (cm^−1^): 3200–2700 (N-H stretching), 1654 (C=O stretching), 1350 (N-O stretching); ^1^H NMR (400 MHz, DMSO) δ 12.87 (s, 1H, NH), 8.28–8.21 (m, 2H, Ar-H), 7.99–7.84 (m, 4H, Ar-H), 7.70 (d, 1H, *J* = 8.1 Hz, Ar-H), 7.62 (t, 1H, *J* = 7.6 Hz, Ar-H); ^13^C NMR (101 MHz, DMSO) δ 162.41 (C=O), 152.57 (C=N), 149.36 (Ar-C-NO_2_), 148.35 (Ar-Cq), 135.48 (Ar-CH), 134.74 (Ar-CH), 132.38 (Ar-CH), 132.34 (Ar-CH), 130.08 (Ar-Cq), 128.17 (Ar-CH), 127.95 (Ar-CH), 126.75 (Ar-CH), 125.36 (Ar-CH), 122.06 (Ar-Cq); HRMS *m*/*z* [M+H]^+^ calcd; C_14_H_9_N_3_O_3_^+^, 268.0722, found 268.0880. mp 230.6–232.1 °C.

#### 2.3.8. 2-(3-Nitrophenyl)-3*H*-quinazolin-4-one (**3h**)

Recrystallization solvent: mixed solvent system of DMF and water; off white fiber-like crystalline solid (0.88 g, 72%) FTIR (cm^−1^): 3200–2800 (N-H stretching), 1670 (C=O stretching), 1350 (N-O stretching); ^1^H NMR (400 MHz, DMSO) δ 12.91 (s, 1H, NH), 9.07 (s, 1H, Ar-H), 8.66 (d, *J* = 8.1 Hz, 1H, Ar-H), 8.48 (d, *J* = 7.9 Hz, 1H, Ar-H), 8.22 (d, *J* = 7.7 Hz, 1H, Ar-H), 7.94–7.82 (m, 3H, Ar-H), 7.62 (t, *J* = 8.0 Hz, 1H, Ar-H); ^13^C NMR (101 MHz, DMSO) δ 164.34 (C=O), 152.73 (C=N), 149.96 (Ar-C-NO_2_), 149.36 (Ar-Cq), 136.51 (Ar-Cq), 135.79 (Ar-CH), 135.36 (Ar-CH), 131.59 (Ar-CH), 128.83 (Ar-CH), 128.16 (Ar-CH), 127.28 (Ar-CH), 126.94 (Ar-CH), 124.00 (Ar-CH), 122.66 (Ar-Cq); HRMS *m*/*z* [M+H]^+^ calcd; C_14_H_9_N_3_O_3_^+^, 268.0722, found 268.0870. mp 362.7–364.2 °C.

#### 2.3.9. 2-(4-Nitrophenyl)-3*H*-quinazolin-4-one (**3i**)

Recrystallization solvent: mixed solvent system of DMF and water; off white fiber-like crystalline solid (1.52 g, 65%) FTIR (cm^−1^): 3211–2741 (N-H stretching), 1671 (C=O stretching), 1350 (N-O stretching); ^1^H NMR (400 MHz, DMSO) δ 12.92 (s, 1H, NH), 9.11–8.17 (m, 4H, Ar-H), 7.92 (d, *J* = 7.5 Hz, 1H, Ar-H), 7.91–7.88 (m, 1H, Ar-H), 7.89 (d, *J* = 8.4 Hz, 1H, Ar-H), 7.62 (t, *J* = 7.4 Hz, 1H, Ar-H); ^13^C NMR (101 MHz, DMSO) δ 162.02 (C=O), 150.73 (C=N), 149.00 (Ar-C-NO_2_), 148.34 (Ar-Cq), 138.55 (Ar-Cq), 134.80 (Ar-CH), 129.30 (Ar-CH), 127.73 (Ar-CH), 127.36 (Ar-CH), 125.91 (Ar-CH), 123.62 (Ar-CH), 121.24 (Ar-Cq); HRMS *m*/*z* [M+H]^+^ calcd; C_14_H_9_N_3_O_3_^+^, 268.0722, found 268.1045. mp 322.1–342.9 °C.

#### 2.3.10. 2-(4-Bromophenyl)-3*H*-quinazolin-4-one (**3j**)

Recrystallization solvent: mixed solvent system of DMF and water; off white fiber-like crystalline solid (1.35 g, 71%) FTIR (cm^−1^): 3200–2800 (broad) (N-H stretching), 1670 (C=O stretching), 771 (C-Br stretching); ^1^H NMR (400 MHz, DMSO) δ 12.65 (s, 1H, NH), 8.24–8.13 (m, 3H, Ar-H), 7.94–7.74 (m, 4H, Ar-H), 7.69–7.49 (m, 1H, Ar-H); ^13^C NMR (101 MHz, DMSO) δ 162.92 (C=O), 152.24 (C=N), 149.21 (Ar-Cq), 135.30 (Ar-CH), 132.67 (Ar-Cq), 132.28 (Ar-C-Br), 131.89 (Ar-CH), 130.48 (Ar-CH), 129.73 (Ar-CH), 128.11 (Ar-CH), 127.42 (Ar-CH), 126.55 (Ar-CH), 125.88 (Ar-CH), 121.70 (Ar-Cq); HRMS *m*/*z* [M+H]^+^ (^79^Br) calcd; C_14_H_10_BrN_2_O^+^, 300.9971, found 300.9951, [M+H]^+^ (^81^Br) calcd; C_14_H_10_BrN_2_O^+^, 302.9950, found 302.9930. mp 315.4–316.6 °C.

#### 2.3.11. 2-(4-Flurophenyl)-3*H*-quinazolin-4-one (**3k**)

Recrystallization solvent: mixed solvent system of DMF and water; white fiber-like crystalline solid (1.40 g, 80%) FTIR (cm^−1^): 3259–2830 (N-H stretching), 1659 (C=O stretching); ^1^H NMR (400 MHz, DMSO) δ 12.68 (s, 1H, NH), 8.64–8.13 (m, 3H, Ar-H), 8.06–7.63 (m, 2H, Ar-H), 7.67–7.32 (m, 3H, Ar-H); ^13^C NMR (101 MHz, DMSO) δ 165.77 (Ar-C-F), 162.68 (C=O), 151.87 (C=N), 149.15 (Ar-Cq), 135.12 (Ar-CH), 130.90 (Ar-CH), 129.74 (Ar-Cq), 127.95 (Ar-CH), 127.10 (Ar-CH), 126.34 (Ar-CH), 121.38 (Ar-Cq), 116.21 (Ar-CH); HRMS *m*/*z* [M+H]^+^ calcd; C_14_H_9_FN_2_O^+^, 241.0777, found 241.1067. mp 283.5–285.8 °C.

#### 2.3.12. 2-(2,4-Dimethoxyphenyl)-3*H*-quinazolin-4-one (**3l**)

Recrystallization solvent: mixed solvent system of DMF and water; white fiber-like crystalline solid (1.510 g, 73%) FTIR (cm^−1^): 3300–2800 (N-H stretching), 1677 (C=O stretching); ^1^H NMR (400 MHz, DMSO) δ 11.84 (s, 1H, NH), 8.16 (d, *J* = 7.9 Hz, 1H, Ar-H), 7.91–7.68 (m, 3H, Ar-H), 7.53 (d, *J* = 7.6 Hz, 1H, Ar-H), 6.81–6.66 (m, 2H, Ar-H), 3.91 (s, 6H, OCH_3_); ^13^C NMR (101 MHz, DMSO) δ 163.42 (C=O), 161.63 (Ar-C-OCH_3_), 159.27 (Ar-C-OCH_3_), 152.36 (C=N), 149.66 (Ar-Cq), 134.82 (Ar-CH), 132.20 (Ar-CH), 127.72 (Ar-CH), 126.59 (Ar-CH), 126.23 (Ar-CH), 121.19 (Ar-Cq), 115.05 (Ar-CH), 106.40 (Ar-CH), 99.03 (Ar-CH), 56.50 (OCH_3_), 56.05 (OCH_3_); HRMS *m*/*z* [M+H]^+^ calcd; C_16_H_14_N_2_O_3_^+^, 283.1082, found 283.1375. mp 160.2–162.3 °C.

#### 2.3.13. 2-(2,5-Dimethoxyphenyl)-3*H*-quinazolin-4-one (**3m**)

Recrystallization solvent: mixed solvent system of DMF and water; white fiber-like crystalline solid (1.35 g, 65%) FTIR (cm^−1^): 3230 (N-H stretching), 1663 (C=O stretching); ^1^H NMR (400 MHz, DMSO) δ 12.11 (s, 1H, NH), 8.19 (d, *J* = 7.9 Hz, 1H, Ar-H), 7.92–7.69 (m, 2H, Ar-H), 7.57 (t, *J* = 7.4 Hz, 1H, Ar-H), 7.40–7.12 (m, 3H, Ar-H), 3.83 (s, 6H, OCH_3_); ^13^C NMR (101 MHz, DMSO) δ 161.83 (C=O), 153.65 (Ar-C-OCH_3_), 152.61 (Ar-C-OCH_3_), 152.01 (C=N), 149.61 (Ar-Cq), 135.09 (Ar-CH), 128.11 (Ar-CH), 127.26 (Ar-CH), 126.45 (Ar-Cq), 123.63 (Ar-CH), 121.71 (Ar-Cq), 118.25 (Ar-CH), 115.97 (Ar-CH), 113.98 (Ar-CH), 56.94 (OCH_3_), 56.37 (OCH_3_); HRMS *m*/*z* [M+H]^+^ calcd; C_16_H_14_N_2_O_3_^+^, 283.1082, found 283.1380. mp 139.6–141.5 °C.

#### 2.3.14. 2-(3,4,5-Trimethoxyphenyl)-3*H*-quinazolin-4-one (**3n**)

Recrystallization solvent: mixed solvent system of DMF and water; white fiber-like crystalline solid (1.55 g, 68%) FTIR (cm^−1^): 3290–2793 (N-H stretching), 2361, 1664 (C=O stretching); ^1^H NMR (400 MHz, DMSO) δ 12.56 (s, 1H, NH), 8.19 (dd, *J* = 7.9, 1.2 Hz, 1H, Ar-H), 7.93–7.70 (m, 2H, Ar-H), 7.60 (s, 2H, Ar-H), 7.55 (t, *J* = 7.9 Hz, 1H, Ar-H), 3.83 (s, 9H, OCH_3_); ^13^C NMR (101 MHz, DMSO) δ 162.79 (C=O), 153.32 (Ar-C-OCH_3_), 152.19 (C=N), 149.09 (Ar-Cq), 140.75 (Ar-C-OCH_3_), 135.02 (Ar-CH), 128.11 (Ar-CH), 127.86 (Ar-CH), 126.87 (Ar-Cq), 126.28 (Ar-CH), 121.26 (Ar-Cq), 105.71 (Ar-CH), 60.59 (OCH_3_), 56.60 (OCH_3_); HRMS *m*/*z* [M+H]^+^ calcd; C_17_H_16_N_2_O_4_^+^, 313.1188, found 313.1509. mp 264.4–265.2 °C.

#### 2.3.15. 2-(4-Hydroxy-3-methoxyphenyl)-3*H*-quinazolin-4-one (**3o**)

Recrystallization solvent: mixed solvent system of DMF and water; brown needle-like crystalline solid (1.39 g, 71%) FTIR (cm^−1^): (3500–3300) (O-H stretching), (3200–3000) (N-H stretching), 1658 (C=O stretching); ^1^H NMR (400 MHz, DMSO) δ 12.38 (s, 1H, NH), 9.81 (s, 1H, OH), 8.16 (dd, *J* = 7.9, 1.2 Hz, 1H, Ar-H), 7.88–7.67 (m, 4H, Ar-H), 7.50 (t, *J* = 7.0 Hz, 1H, Ar-H), 6.96 (d, *J* = 7.9 Hz, 1H, Ar-H), 3.96 (s, 3H, OCH_3_); ^13^C NMR (101 MHz, DMSO) δ 163.23 (C=O), 152.91 (C=N), 150.87 (Ar-C-OH), 149.91 (Ar-C-OCH_3_), 148.39 (Ar-Cq), 135.36 (Ar-CH), 128.11 (Ar-CH), 126.69 (Ar-CH), 124.30 (Ar-Cq), 122.39 (Ar-CH), 121.49 (Ar-Cq), 116.46 (Ar-CH), 116.33 (Ar-CH), 112.31 (Ar-CH), 56.72 (OCH_3_); HRMS *m*/*z* [M+H]^+^ calcd; C_15_H_12_N_2_O_3_^+^, 269.1274, found 269.1078; mp 260.2–262.6 °C.

### 2.4. Alpha-Amylase Inhibition

Sample (200 μL) (5.000–0.312 mM) was dissolved in DMSO, and alpha-amylase (200 μL) (1 mg/mL) was added to each tube and incubated at room temperature for 10 min. Then, starch (200 μL) (0.5% *w*/*v*) was added, and the tubes were incubated at 37 °C for another 10 min. After that, DNSA reagent (200 μL) was added, and the tubes were boiled for 5 min. Finally, the absorbance was measured at 540 nm using a microplate reader. Acarbose (500 μL) (2.000–0.125 mM) was taken as the standard antidiabetic drug. All concentrations were triplicated and reported as mean ± standard deviation [28]. The percentage inhibition of each compound at different concentrations was determined by using Equation (1):

(1) $$\%\;\mathrm{Inhibition}\;=\frac{A\left(control\right)-A\left(test\right)}{A\left(control\right)}\;\times \;100$$

### 2.5. Alpha-Glucosidase Inhibition

A series of compound concentrations (10.00–0.25 mM) and acarbose concentrations (0.40–0.10 mM) were prepared accordingly. A sample/standard (40 μL) from each dilution was mixed with α-glucosidase (20 μL, 1 U/mL) and sodium phosphate buffer (100 μL), and the mixture was incubated at 37 °C for 15 min. Then, p-NPG (40 μL, 2 mM) was added, followed by incubation at 37 °C for another 20 min. The reaction was terminated by adding sodium carbonate (200 μL, 0.10 M). The absorbance was then measured at 405 nm using a microplate reader [29,30]. Acarbose was used as the standard antidiabetic drug. All concentrations were tested in triplicate and presented as mean ± standard deviation. The percentage inhibition of α-glucosidase activity was calculated using Equation (1).

### 2.6. Analysis of Glucose Uptake by Saccharomyces cerevisiae

*Saccharomyces cerevisiae* (bakers’ yeast) was subjected to centrifugation at 3000× *g* for 5 min. The supernatant was then discarded, and the pellet was centrifuged until a clear supernatant was observed. A 10% yeast solution was then prepared. A glucose solution (500 μL) (500 mM) was added to the sample solutions (500 μL) (100–20 mM), which were dissolved in DMSO. It was incubated at 37 °C for 10 min. The yeast solution (50 μL) and DNSA reagent (500 μL) were added and further incubated at 37 °C for 1 h. After 1 h, the solution mixture was boiled for 5 min. The tubes were centrifuged at 2500× *g* for 5 min and glucose was estimated in the supernatant, where the absorbance of solution mixtures was taken at 540 nm using a microplate reader (MULTISKAN Sky, Type 1530, Thermo Fisher Scientific, Waltham, MA, USA). Metformin (500 μL) (50–10 mM) was taken as the standard antidiabetic drug. All concentrations were triplicated and reported as mean ± standard deviation [5,7]. Percentage uptake of each compound at different concentrations was determined by using Equation (2).

(2) $$\%\;\mathrm{Glucose}\;\mathrm{uptake}=\frac{A\left(control\right)-A\left(test\right)}{A\left(control\right)}\;\times \;100$$

### 2.7. Statistical Analysis

Statistical analysis, to determine significant differences between electron-donating groups vs. electron-withdrawing groups and the position of substitution, was performed using the one-way ANOVA function in Microsoft Excel. A *p*-value of <0.05 was considered statistically significant.

### 2.8. In Silico Study

#### 2.8.1. Homology Modeling

Homology modeling was utilized to generate the 3D structure of the GLUT2 protein. A FASTA sequence (ID: P11168) containing 524 amino acid residues was obtained from the UniProt database [31]. The template P11168.1. A model with 100% total coverage was chosen to build the model based on GMQE; it achieved 0.87 using SWISS-MODEL software [32]. The protein was visualized via Discovery Studio Visualizer 2024 [6].

#### 2.8.2. Molecular Docking

The protein structures for pancreatic alpha-amylase (PDB: 4GQR) and alpha-glucosidase (PDB: 5NN8) were downloaded from the RSCB Protein Data Bank [33]. The Hydrogen atoms, the initial ligand present in the protein, and the water molecules were removed to get the clean protein using Discovery Studio. All ligands (synthesized molecules) obtained from the SDF format were obtained from the PubChem [34] database. Molecular docking was conducted using all default algorithms via AutoDock Vina PyRx-0.8 (PyRx Virtual Screening Tool). The grid box in AutoDock Vina was generated targeting the whole protein, where the dimensions for the GLUT2 protein were X = 112.4529, Y = 61.8964, Z = 77.7535, with centers at X = 9.2263, Y = 0.4137, Z = 2.7165. For alpha-amylase (PDB: 4GQR) enzyme, the grid box dimensions were at X = 58.0962, Y = 73.9016, Z = 58.5893, while centers were at X = 8.4487, Y = 27.9817, Z = 49.1306. Alpha-glucosidase (PDB: 5NN8) enzyme dimensions were X = 103.9171, Y = 93.7291, Z = 104.9702, while centers were at X = 3.6507, Y = −30.3167, Z = 83.0597. All the x, y, z coordinates were given in angstrom units. The binding affinities (kcal/mol) of the ligands were used to evaluate the docking results and to compare each ligand’s binding affinity. The interactions between ligand and protein receptor and the respective amino acid were visualized using Discovery Studio software [35,36].

#### 2.8.3. ADMET Profile Prediction

Using SMILES from PubChem database, (ADMET) profiles of compounds were obtained using admetSAR 3.0 [7,37].

## 3. Results and Discussions

### 3.1. Alpha-Amylase Inhibition

**Figure 1:**
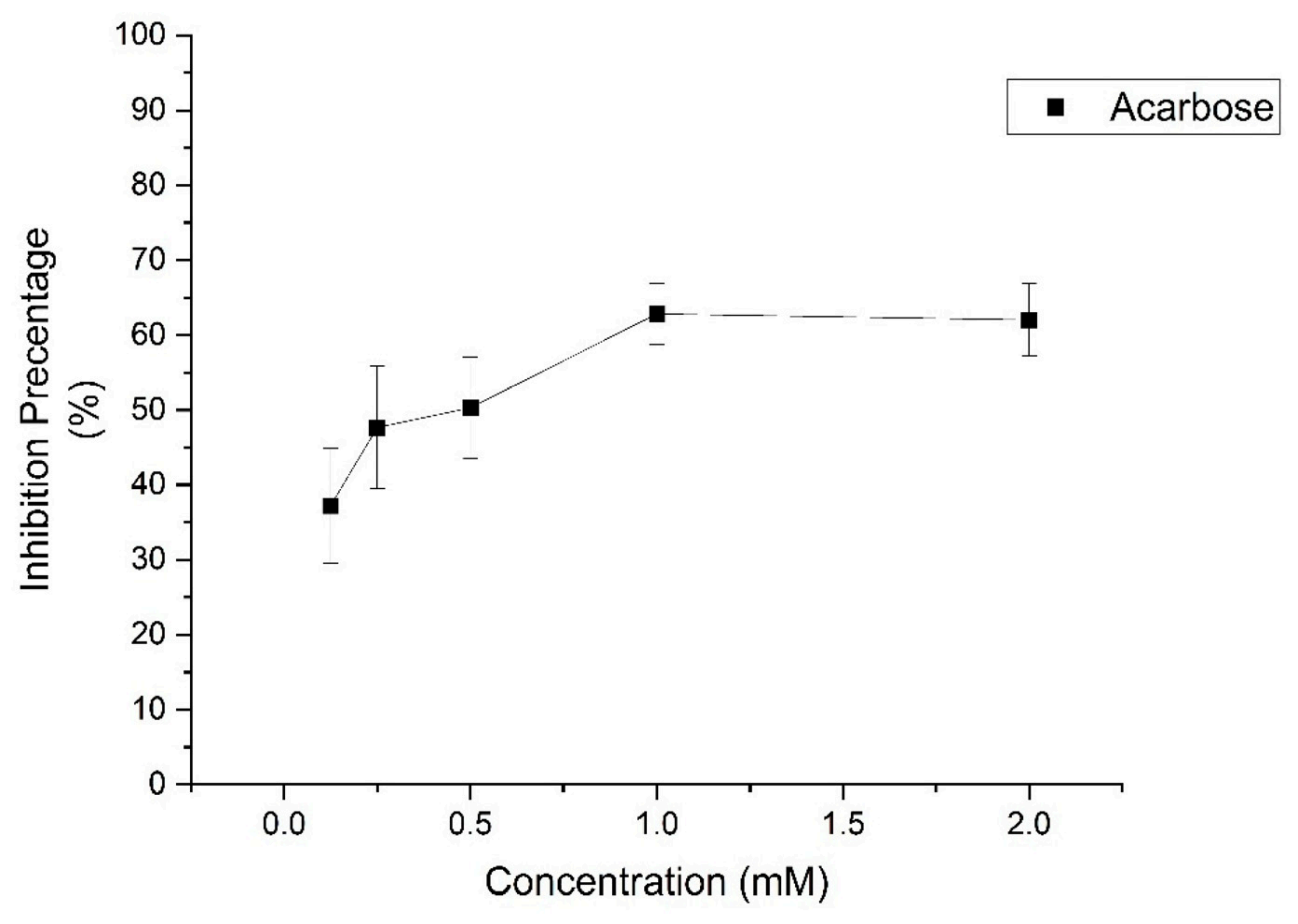
Percentage inhibition of α-amylase enzyme activity by the standard antidiabetic drug, acarbose, tested at concentrations of 2.000–0.125 mM.

**Figure 2:**
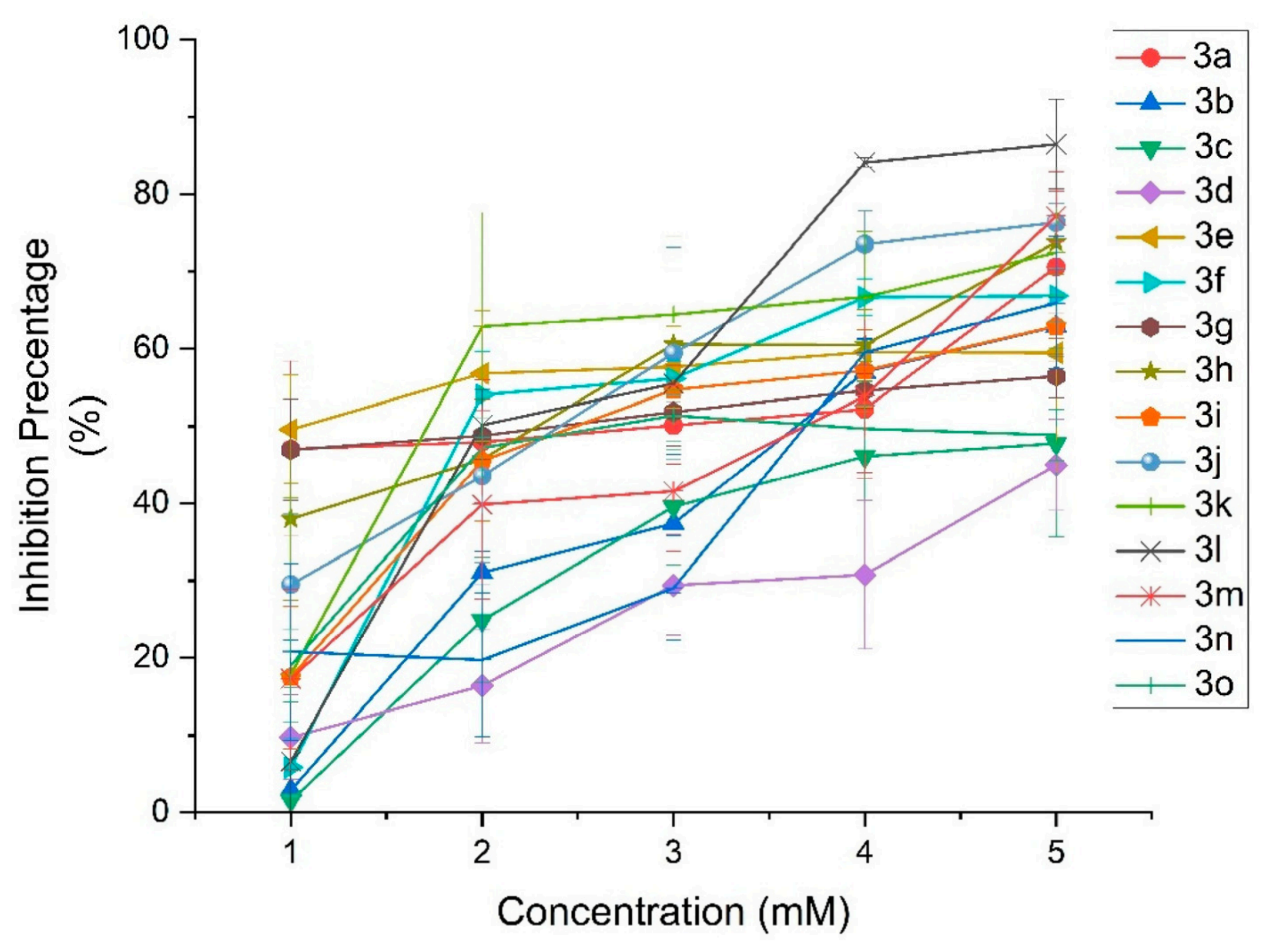
Percentage inhibition of α-amylase enzyme activity by the synthesized 2-phenyl-3*H*-quinazolin-4-one derivatives (**3a**–**3o**), tested at concentrations of 5.000–0.312 mM.

The α-amylase enzyme facilitates carbohydrate digestion, leading to uncontrolled postprandial glucose elevations in individuals with diabetes. Suppressing its activity represents an effective approach for managing hyperglycemia and mitigating the progression of diabetes [29]. This bioassay expressed the concentration required to inhibit 50% of alpha-amylase activity in mM concentration (Table 1). Figure 1 shows the inhibitory activity of the standard drug acarbose (IC_50_ = 0.53 mM), which serves as a benchmark for comparison, while Figure 2 illustrates the different degrees of potency exhibited by the tested compounds in inhibiting alpha amylase enzyme.Lower concentrations corresponding to IC_50_ values indicate higher inhibitory activity, whereas higher concentrations indicate lower inhibitory activity.

The relative inhibition of enzymes by these synthetic phenyl derivatives can be explained by the electronic effect of the substituents in the synthetic molecules and the positioning of these substituents. The moderate inhibition observed with the unsubstituted phenyl compound (0.80 mM) underscores the importance of testing ortho-, para-, and meta-substituted compounds. The lowest IC_50_ values were observed for ortho-methoxy and para-halogen derivatives. One-way ANOVA (*p* > 0.05) confirmed that both electron-donating and electron-withdrawing groups can yield high inhibition, consistent with bioassay findings.

The ortho-methoxy derivative, **3e**, has the lowest IC_50_ value (0.43 mM). The ortho-nitro derivative shows the lowest IC_50_ (1.18 mM) among nitro-substituted compounds. The nitro group withdraws electrons from the aromatic ring via resonance, reducing electron density and thereby affecting activity. Ortho-hydroxy derivatives exhibited higher activity than their meta- or para-substitutions, while meta-substitution generally gave weaker activity. Compound **3o**, which contains 4-hydroxy and 3-methoxy groups, has an IC_50_ of 1.09 mM. It is interesting to note that the activity increased when meta-substitution and para-substitution were combined. This indicates favorable synergistic interactions. At the para position, halogen-containing compounds such as **3j** (0.71 mM) and **3k** (0.70 mM) show improved activity. In contrast, electron-donating groups at the para position show reduced activity compared to ortho- or meta-substitutions. Steric hindrance likely explains why derivatives with two or three methoxy groups (3**l**–**n**) often show decreased activity, as bulky substituents prevent proper accommodation in the enzyme active site.

### 3.2. Alpha-Glucosidase Inhibition

Alpha-glucosidase is a hydrolase enzyme that is present in the brush border surface of the intestinal cells [38]. It works to facilitate the absorption of glucose by the small intestine by catalyzing the hydrolytic cleavage of oligosaccharides into absorbable monosaccharides [39,40]. Alpha-glucosidase inhibitors slow down the digestion of oligosaccharides into monosaccharides in the intestine and hence reduce the glucose level in the bloodstream, including postprandial hyperglycemia [40]. Alpha-glucosidase inhibitors are utilized orally to treat type 2 diabetes mellitus.

**Figure 3:**
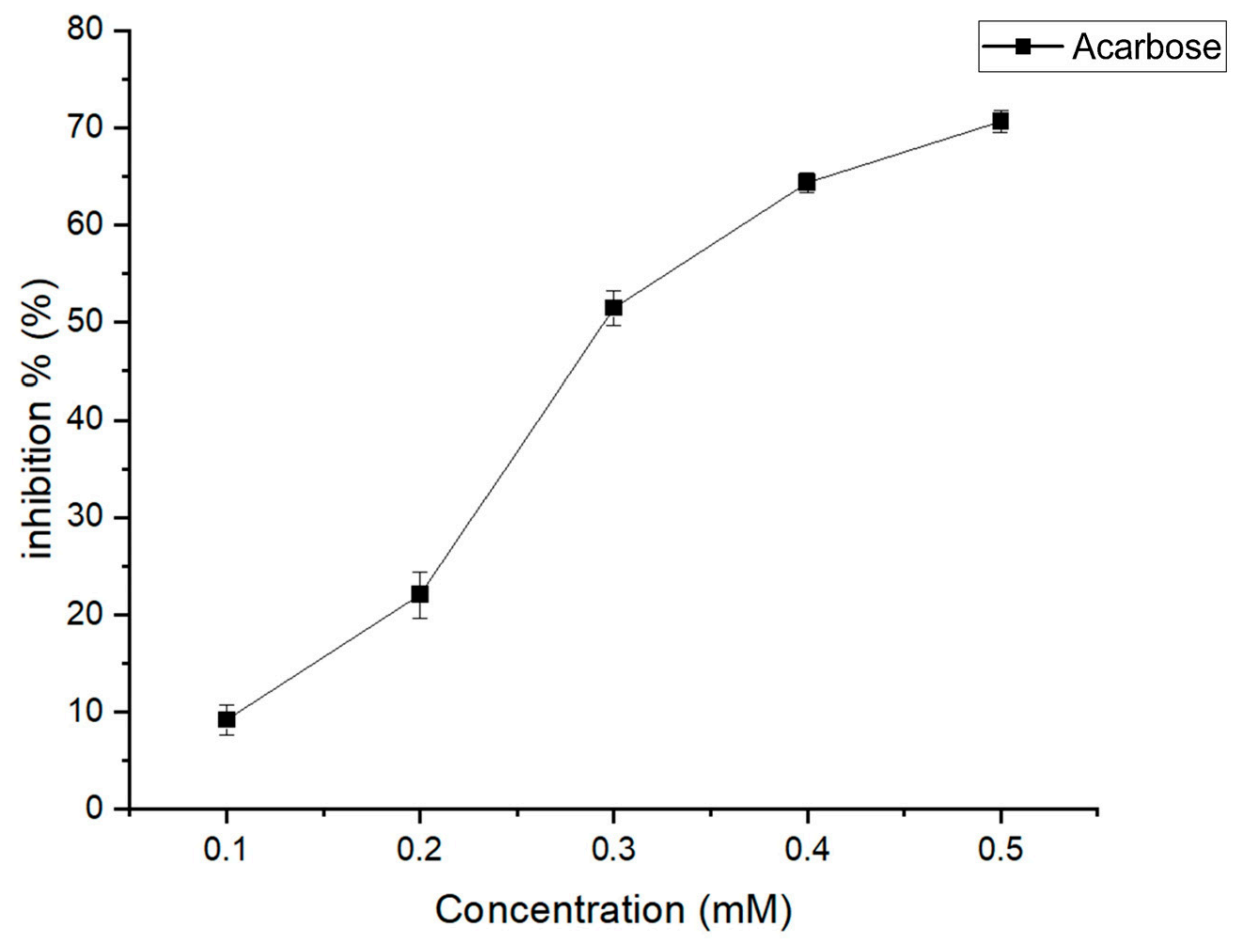
Percentage inhibition of α-glucosidase enzyme activity by the standard antidiabetic drug acarbose, tested at concentrations of 0.40–0.10 mM.

**Figure 4:**
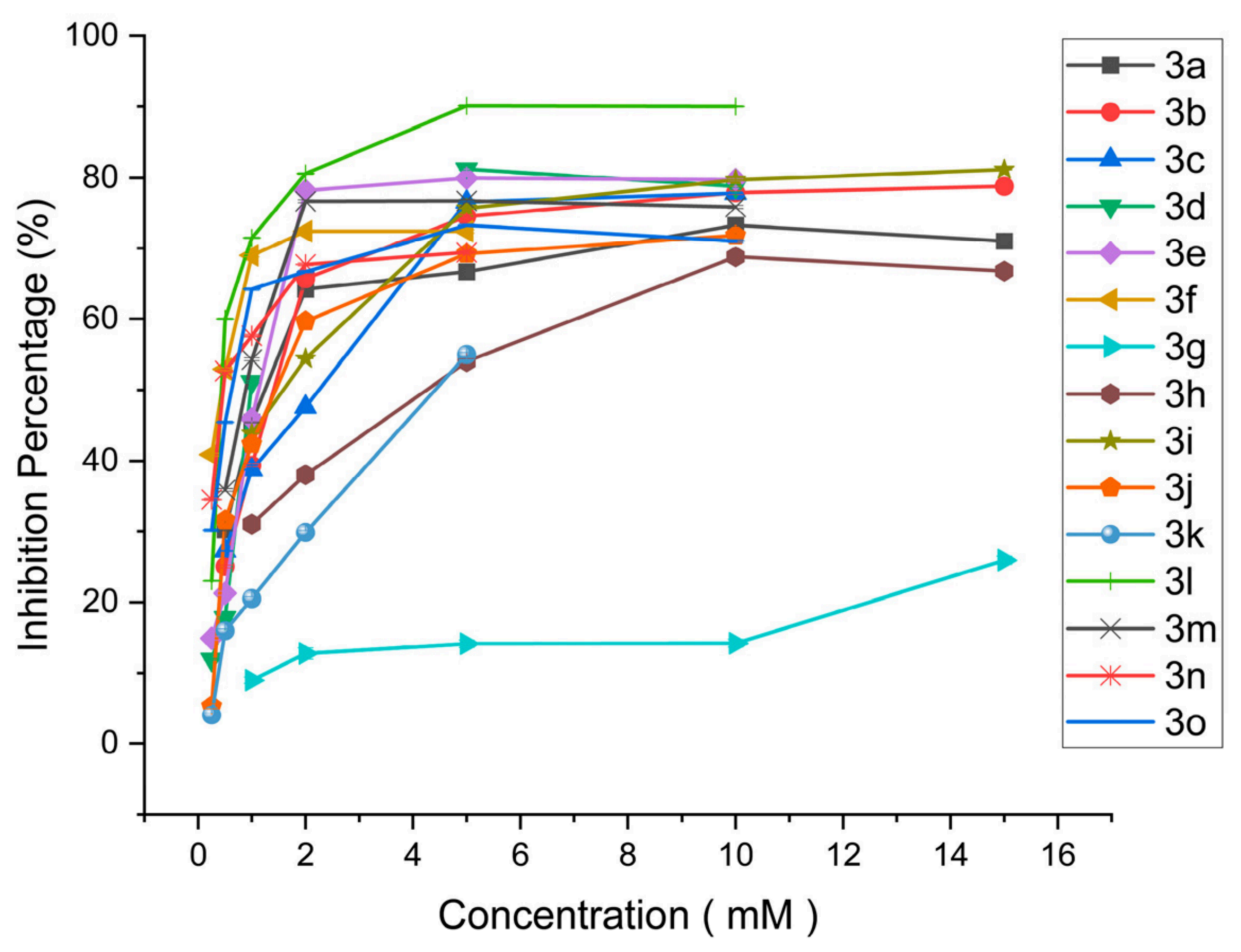
Percentage inhibition of α-glucosidase enzyme activity by the synthesized 2-phenyl-3*H*-quinazolin-4-one derivatives (**3a**–**3o**), tested at concentrations of 10.00–0.25 mM.

Figure 3 shows the inhibitory activity of the standard drug acarbose (IC_50_ = 0.29 mM), which serves as a benchmark for comparison, while Figure 4 illustrates the different degrees of potency exhibited by the synrthesized ompounds in inhibiting alpha glucosidase enzyme. The synthesized compounds displayed IC_50_ values ranging from 0.42 to 4.14 mM. The inhibitory activity of the synthesized derivatives (Table 1) was strongly influenced by both the position and electronic nature of the phenyl-ring substituents. 

The most potent inhibitors were shown by the para position, with the **3f** compound exhibiting higher activity (0.44 mM) than the **3d** compound (0.98 mM), highlighting the effect of electron-donating groups at this position. Halogen substitution at the para position showed variable inhibition, with the **3j** compound showing moderate activity (1.20 mM) and the **3k** compound being substantially less active (4.14 mM). The strongly electron-withdrawing nitro group significantly reduced activity (1.58 mM). Weaker inhibitory effects were displayed in meta-substituted derivatives, with the **3c** and **3h** compounds showing IC_50_ values of 2.02 and 4.12 mM, respectively, confirming that this position is less favorable for productive enzyme interaction. Moderate activity results in ortho substitution with **3b** (1.34 mM) and **3e** (1.06 mM), while the **3g** compound showed insufficient inhibition to determine the IC_50,_ likely due to steric hindrance impeding binding.

Markedly enhanced activity was displayed by the di- and tri-substituted derivatives as **3l** has the lowest IC_50_ value (0.42 mM), and the compounds **3m** (0.92 mM), **3n** (0.53 mM), and mixed hydroxy–methoxy derivative **3o** (0.57 mM) also showed higher inhibitions than the monosubstituted. These findings clearly showed that the introduction of multiple electron-donating groups produces a synergistic enhancement of α-glucosidase inhibitory activity. ANOVA (*p *> 0.05) indicated no significant differences among substituent positions, supporting the observation that both electron-donating and electron-withdrawing groups can contribute to inhibition depending on their electronic properties and the nature of their interactions with the target enzyme.

According to previously reported data, SAR analysis of chloro- and bromo-substituted 2-phenyl-3*H*-quinazolin-4-one derivatives show alpha-glucosidase inhibition potential, with IC_50_ values ranging from 12.50 ± 0.10 to 15.60 ± 0.10 μM, respectively, while nitro- and fluoro-substituted derivatives give decreased inhibition [41]. This reported trend is consistent with the results observed in the present study.

### 3.3. Glucose Uptake by Saccharomyces cerevisiae

**Figure 5:**
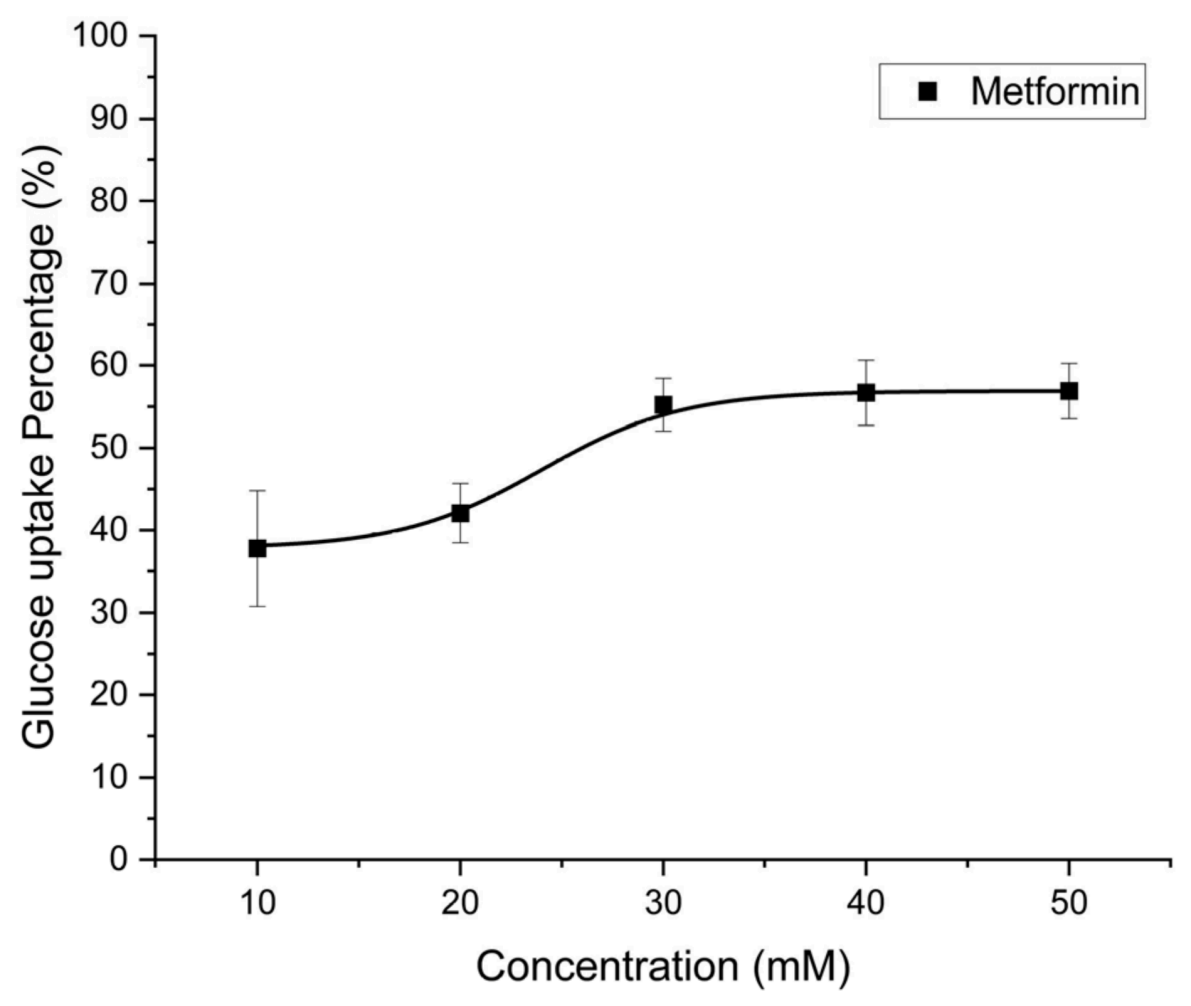
Glucose uptake percentage (%) by *Saccharomyces cerevisiae* in the presence of the standard antidiabetic drug, metformin, tested at concentrations of 50–10 mM.

**Figure 6:**
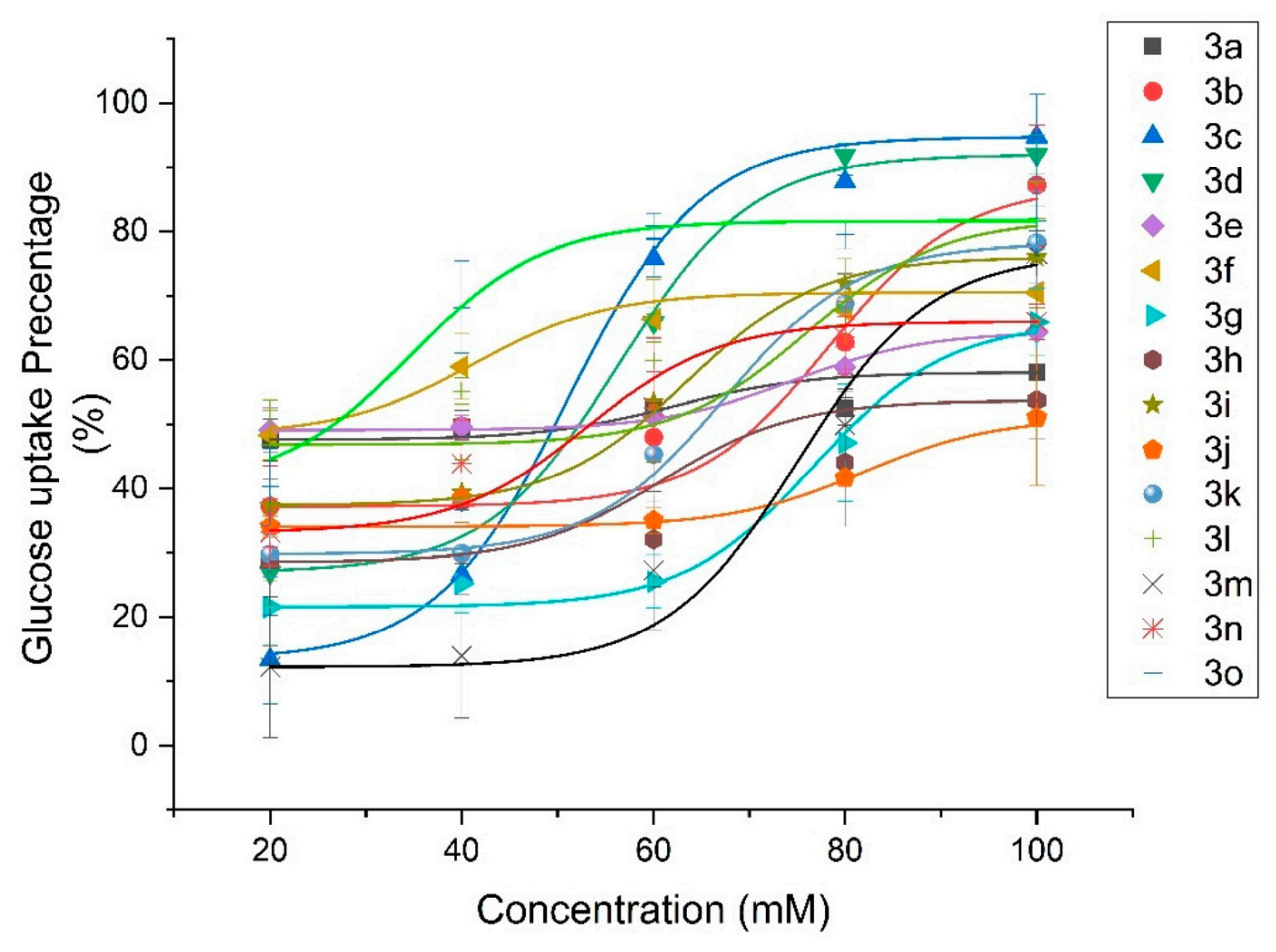
Glucose uptake percentage(%) by S. *cerevisiae* for the synthesized 2-phenyl-3*H*-quinazolin-4-one derivatives (**3a**–**3o**), tested at concentrations of 100–20 mM.

Yeast cells exhibit several key eukaryotic biological processes similar to humans, including vesicular transport and metabolic pathways [42]. Therefore, yeast cells play a significant role in antidiabetic research. The glucose uptake by yeast is expressed as the concentration of each tested compound required to achieve 50% uptake (Table 1). Figure 5 presents the glucose uptake activity of the standard drug metformin (50% glucose uptake at 25.99 mM), which serves as a benchmark, while Figure 6 illustrates the relative potencies of the tested compounds in promoting glucose uptake. Although yeast provides a cost-effective and physiologically relevant model, its lack of insulin-mediated regulation requires validation in mammalian systems [9,10]. The unsubstituted phenyl derivative **3a** showed only moderate glucose uptake potency (40.11 mM), indicating that substitution on the aromatic ring is essential for activity variations.

Hydroxy (**3b**, 47.04 mM) and methoxy (**3e**, 50.14 mM) substitutions at the ortho position show high potency, while the nitro analog (**3g**, 81.99 mM) shows much weaker potency. A similar trend was observed at the meta position. The methoxy derivative **3c** (49.74 mM) has better activity than the nitro analog **3h** (72.91 mM), but less significant than at ortho or para. The para–methoxy compound **3f** showed the highest potency among monosubstituted derivatives (24.02 mM), surpassing para–hydroxy (**3d**, 55.67 mM) and para–nitro (**3k**, 64.22 mM). The very weak activity of the compound **3j** (93.69 mM) highlights the effect of electron-withdrawing substituents. Given the enhanced potency of methoxy groups in monosubstituted derivatives, the testing for disubstituted and tri-substituted derivatives was conducted. The synergistic electronic contribution from ortho and para positions is highlighted in compound 3**l**. However, introducing an additional methoxy group at the meta position, as in **3m**, significantly reduces potency (80.10 mM), suggesting that meta substitution is unfavorable. A similar reduction was observed due to meta-substitution and steric hindrance in the tri-substituted methoxy compound, **3n**, which has a lower potency of 53.54 mM. Compound **3o** (26.63 mM) retained high potency despite containing a meta substituent, indicating that certain combinations remain favorable. As in many approved drugs in the market, methoxy groups can increase lipophilicity, improving cell membrane permeability [43]. This explains how the incorporation of methoxy-containing compounds can increase glucose uptake by yeast cells.

ANOVA (*p *< 0.05) showed significant differences between electron-donating and electron-withdrawing groups, confirming that electron-donating substituents consistently yielded higher potency. However, no significant differences (*p *> 0.05) were observed among ortho, meta, and para positions, consistent with bioassay results.

Overall, the ortho and para substituents have greater efficacy than the meta substituents due to the transmission of resonance electron donation through the aromatic ring. Electron donation from an electron-donating group like methoxy occurs through resonance, in which the electron donation takes place into the π-system of the aromatic ring, but the donated electrons reach the carbons of the ring in ortho and para positions. However, the meta position receives no such resonance contribution from the aromatic ring, making it less active than others.

The different behaviors of electron-donating and electron-withdrawing substituents can be explained electronically. The hydroxyl and methoxy substituents donate the electron density into the aromatic ring via resonance, causing an increase in the electron density, and the methoxy groups contribute to the increase in lipophilicity that favors membrane permeation, explaining the higher yeast glucose uptake activity of 3f [43]. In contrast, the nitro group is strongly electron-withdrawing by resonance, consistent with the weakest activity in all three assays. Multi-substituted compounds exhibit weak activity, primarily due to steric hindrance, which makes it difficult for the relevant enzyme to accommodate them. This is also supported by the docking results of this study, which show lower binding affinities for multi-substituted compounds.

### 3.4. Molecular Docking

**Figure 7:**
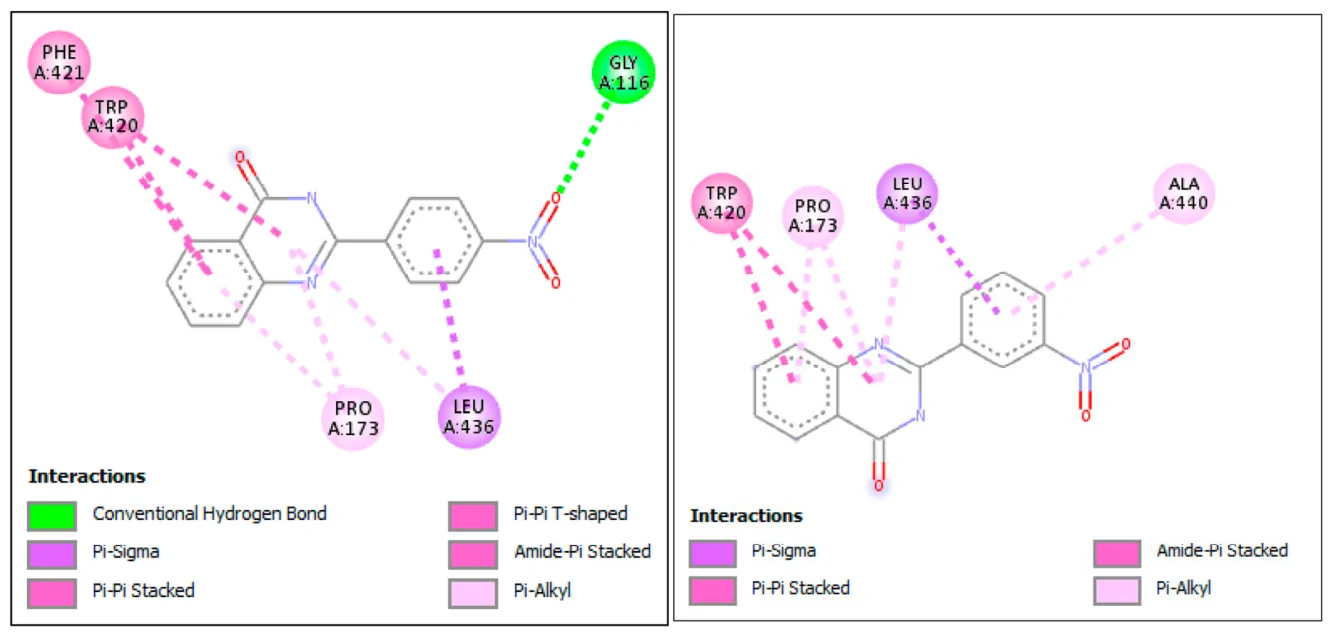
2D protein–ligand interaction diagrams for the top docking poses between GLUT2 protein and compounds (**3i** and **3h**), generated by AutoDock Vina (PyRx Virtual Screening Tool).

Lower binding energies generally indicate stronger binding affinity [32]. Docking results (Table 2) revealed that **3i** has a stronger binding affinity to the GLUT2 protein with a binding energy of −9.6 kcal/mol. Compound **3h** has a binding energy of -9.2 kcal/mol, and **3k** has a binding energy of −9.1 kcal/mol. Compound **3n** has the lowest binding energy among tested compounds with −7.7 kcal/mol energy. The rest of the compounds have moderate binding energies. The mismatch between the docking score and the bioassay results is mainly due to the structural divergence between yeast Hxt transporters and GLUT2. These two transporters are functionally similar, but structurally different from each other [44]. Therefore, we consider the docking results for GLUT2 independent of the yeast assay and instead as a preliminary assessment. Apart from that, docking studies only predict the binding affinity to a fixed/rigid receptor shape without considering the conformational changes during the biological process. Further, static docking does not account for conditions such as incubation time, pH, temperature, or cell permeability-like factors. Therefore, the results of the bioassay might not be compatible with docking results. Interaction diagrams provide a visual representation of how synthesized compounds bind to the binding sites of the GLUT2 protein (Figure 7). The detailed interaction diagrams reveal the specific amino acid residues involved in these binding interactions for the compounds with the best affinity. Compound **3i**, which gives highest binding affinity, has a conventional hydrogen bond with GLY A:116 amino acid residue as a strong polar interaction. Additionally, it forms pi–sigma, pi–pi stacked, pi–pi T-shaped, amide–pi stacked, and pi–alkyl interactions.

**Figure 8:**
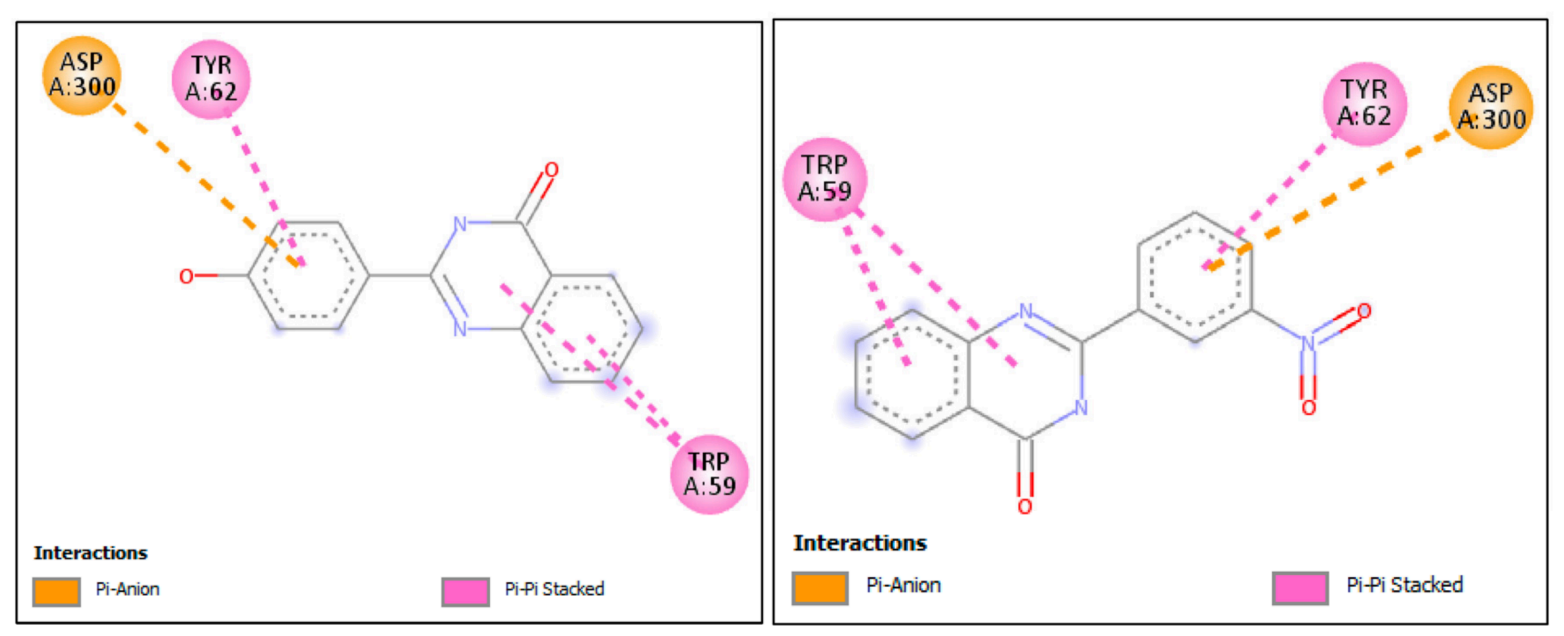
2D protein–ligand interaction diagrams for the top docking poses between α-amylase enzyme and compounds (**3d** and **3h**), generated by AutoDock Vina (PyRx Virtual Screening Tool).

The docking study of quinazolinone derivatives with alpha-amylase shows binding energies ranging from −6.8 to −8.2 kcal/mol (Table 3). The hydroxy and nitro compounds have lower binding energies, indicating greater affinity to the enzyme. Multi-substituted compounds with bulky structures exhibit reduced interactions with the enzyme at its active site. Compounds **3d** and **3h** (−8.2 kcal/mol) (Figure 8) have the highest binding affinities for amylase enzymes among the synthesized derivatives. Both compounds have pi–anion interaction with ASP A:300 and pi–pi stacked interactions with TRP A:59 and TYR A:62. Likewise, some differences were seen between docking and biological results. This means that binding energy alone cannot fully explain enzyme inhibition, and factors like solubility, flexibility, and toxicity may also play a role.

**Figure 9:**
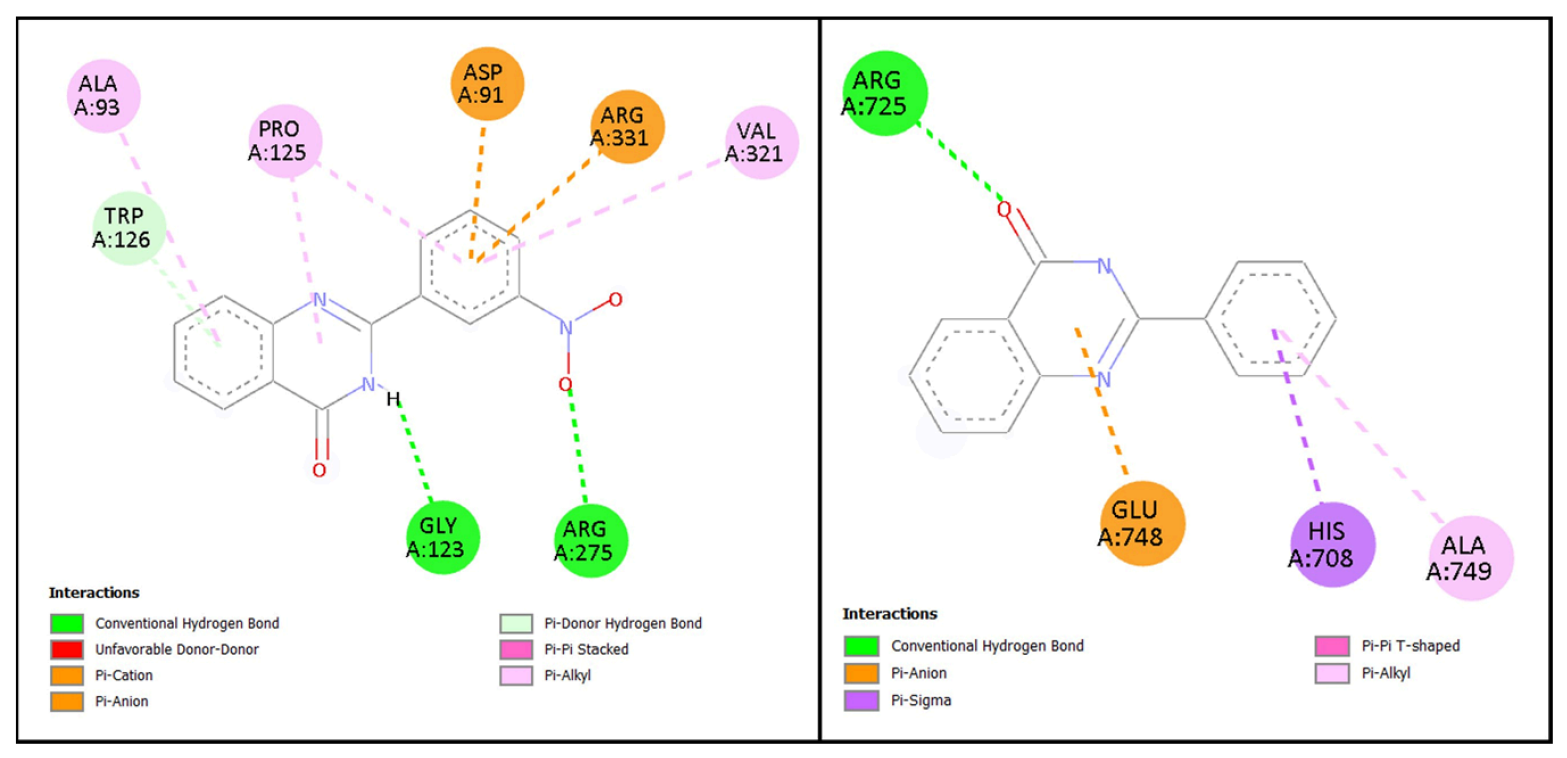
2D protein–ligand interaction diagrams for the top docking poses between α-glucosidase enzyme and compounds (**3h** and **3a**) generated by AutoDock Vina (PyRx Virtual Screening Tool).

Docking scores of interactions between all quinazolinone derivatives and the target enzyme alpha-glucosidase are shown in Table 4. Lower binding energies generally indicate stronger binding affinity, which correlates with higher inhibitory potency. The docking study revealed that **3h** has the highest binding affinity with the α-glucosidase enzyme with a binding energy of −7.9 kcal/mol. Interaction diagrams (Figure 9) provide a visual representation of how synthesized compounds bind to the active sites of alpha-glucosidase enzymes and the specific amino acid residues involved in these binding interactions. It demonstrates that **3h** exhibits interactions with the alpha-glucosidase enzyme, including hydrophobic contacts such as π-alkyl (Ala A:93, Pro A:125, Val A:321), π-anion (Asp A:91, Arg A:331) and H-bond (Arg A:275, Gly A:123), and π-donor H-bond (Trp A:126).

By considering the ADMET profile of these derivatives (**3a**–**3o**), the probability of human toxicity, absorption, metabolism, and secretion was predicted (Table 5). All derivatives satisfy Lipinski’s rule of five, hemolytic toxicity, and eye and skin irritation, supporting a favorable safety profile. Among these compounds, **3e**, **3l**, and **3f**, which gave higher activities for alpha-amylase inhibition, alpha-glucosidase inhibition, and glucose uptake by yeast, showed mixed but acceptable safety profiles with some significant predictions.

Compound **3e**, which has the highest activity for alpha-amylase inhibition, stands out as the best profile among all the compounds by having a negative prediction for mouse, rat, and rodent carcinogenicity, whereas other compounds show positivity towards at least two types. This compound shows an absence of nephrotoxicity and eye and skin irritation, although it raises concern due to potential for respiratory, oral, and reproductive toxicity compared to other compounds.

Compound **3l**, which has the highest activity for alpha-glucosidase inhibition, has potential negative effects for nephrotoxicity and eye and skin irritation, but predicts a positive effect towards rat/mouse/rodent carcinogenicity along with respiratory, oral, and reproductive toxicity.

Compound **3f**, which has the most potent glucose uptake, has a negative prediction for mouse carcinogenicity but not for rat or rodent carcinogenicity and nephrotoxicity. Compared to **3e** and **3l**, **3f** has a positive effect in crustacean toxicity, but the other parameters are similar for all three compounds.

Compounds **3m** and **3n** show negative predictions for nephrotoxicity, compounds **3a**, **3c** and **3o** show negative predictions for acute oral toxicity, and compounds **3a**,**3b** and **3o** show negative predictions for reproductive toxicity.

Overall, the integration of enzyme inhibition, yeast glucose uptake, and molecular docking analyses highlights quinazolinone derivatives as promising scaffolds for dual-action antidiabetic therapy, combining carbohydrate–digestive enzyme inhibition with enhanced cellular glucose regulation.

## 4. Conclusions

This study highlights the antidiabetic activity of 2-phenyl-3*H*-quinazolin-4-one (**3a-o**) through in vitro and in silico molecular docking approaches. These phenyl-substituted quinazolinone derivatives were tested and evaluated for glucose uptake potency and enzyme inhibition of alpha-amylase and alpha-glucosidase. These 2-phenyl-3*H*-quinazolin-4-one derivatives can be easily synthesized in good yields by the oxidative cyclocondensation of 2-aminobenzamide with an aryl aldehyde in the presence of FeCl_3_. Isolation of the product and purification can be done in simple steps, including the addition of EDTA, filtration, and recrystallization. Electron-donating groups at the ortho and para positions increased potency, while bulky or electron-withdrawing groups reduced potency for glucose uptake by yeast. It suggests that steric and electronic effects play crucial roles for glucose transport activity. Compound **3f** shows the highest glucose uptake value. The electron-donating methoxy at the ortho position and electron-withdrawing halogens at the para position showed higher amylase enzyme inhibition. Compound **3e** showed the highest activity for alpha-amylase inhibition. In the same way, the electron-donating groups substituted at the ortho and para positions enhance the alpha-glucosidase inhibitory activity. Compound **3l** gave the highest alpha-glucosidase inhibitory activity. Compounds **3d**, **3i,** and **3h** show the highest binding affinities despite their lower in vitro potency, suggesting that factors such as permeability and toxicity influence the bioassay. ADMET prediction further indicates compounds **3e**, **3f** and **3l as** possessing better safety profiles among other derivatives, supporting their potential as viable candidates for further optimization. Collectively, these results suggest that 2-phenyl-3*H*-quinazolin-4-one derivatives are a promising scaffold for developing antidiabetic therapeutic agents. These findings provide a foundation for future studies involving enzyme kinetics and molecular dynamics simulations to further assess the efficacy and mechanistic behavior of these compounds. Overall, this work opens new directions for the development of improved therapeutic agents for the treatment of diabetics.

## Figures and Tables

**Table 1:** Glucose uptake by yeast (50% uptake), in vitro α-amylase enzyme inhibitory (IC_50_) and in vitro α-glucosidase enzyme inhibitory (IC_50_) activities of the synthesized 2-phenyl-3H-quinazolin-4-one derivatives (**3a**–**3o**) (mean ± standard deviation).

Compounds	Glucose Uptake by Yeast 50% Uptake (mM)	α-Amylase IC_50_ (mM)	α-Glucosidase IC_50_ (mM)
Standard	25.99 ± 2.41 (Metformin)	0.53 ± 0.21 (Acarbose)	0.29 ± 0.50 (Acarbose)
**3a**	40.11 ± 2.56	0.80 ± 0.42	1.17 ± 0.10
**3b**	47.04 ± 2.26	2.08 ± 0.33	1.34 ± 0.25
**3c**	49.74 ± 2.26	NA	2.02 ± 0.08
**3d**	55.67 ± 3.40	NA	0.98 ± 0.15
**3e**	50.14 ± 2.11	0.43 ± 0.05	1.06 ± 0.30
**3f**	24.02 ± 0.96	0.97 ± 0.02	0.44 ± 0.20
**3g**	81.99 ± 2.82	1.18 ± 0.06	NA
**3h**	72.91 ± 3.16	1.56 ± 0.02	4.12 ± 0.37
**3i**	57.01 ± 2.03	1.46 ± 0.50	1.58 ± 0.05
**3j**	93.69 ± 2.13	0.71 ± 0.08	1.20 ± 0.40
**3k**	64.22 ± 1.75	0.70 ± 0.31	4.14 ± 0.30
**3l**	26.84 ± 2.19	1.34 ± 0.11	0.42 ± 0.10
**3m**	80.10 ± 2.28	2.19 ± 0.38	0.92 ± 0.20
**3n**	53.54 ± 2.68	2.10 ± 0.15	0.53 ± 0.20
**3o**	26.63 ± 2.47	1.09 ± 0.12	0.57 ± 0.18

**Table 2:** Molecular docking of the synthesized 2-phenyl-3*H*-quinazolin-4-one derivatives (**3a**–**3o**) with GLUT2: binding energies, interacting GLUT2 amino acid residues, and polar and hydrophobic interactions.

Compounds	Binding Energy (kcal/mol)	Amino Acid Residues	Polar Interactions	Hydrophobic Interactions
**3a**	−8.6	Trp420, Phe421, Pro173, Leu436, Ala440		π−π stacked; Trp420 π-alkyl; Pro173, Leu436, Ala440 amide-πstacked; Trp420, Phe421
**3b**	−8.6	Gly170, Trp420, Phe421, Pro173, Leu436, Ala440	Conventional-H; Gly170	π−π stacked; Trp420 π-alkyl; Pro173, Leu436, Ala440 amide-πstacked; Trp420, Phe421
**3c**	−8.1	Gln315, Trp420, Phe24, Ile319, Ile196		π−π stacked; Trp420 π-alkyl; Ile319, Ile196 π−π T-shaped; Phe24
**3d**	−8.7	Trp420, Phe421, Pro173, Leu436, Ala440		π−σ; Leu436 π−π stacked; Trp420 π−π T-shaped; Phe421 π-alkyl; Pro173, Leu436, Ala440 amide-πstacked; Trp420, Phe421
**3e**	−8.5	His192, Trp420, Phe421, Pro173, Leu436, Ala440, Ala424		π−σ; Leu436 π−π T-shaped; Phe421 alkyl; Pro173 π-alkyl; Pro173, His192, Ala440, Ala424, Trp420 amide-πstacked; Trp420, Phe421
**3f**	−8.9	Ser112, Trp420, Phe421, Pro173, Leu436, Ala440	Carbon-H; Ser112	π−π stacked; Trp420 π-alkyl; Pro173, Leu436, Ala440 π−π T-shaped; Phe421 amide-πstacked; Trp420, Phe421
**3g**	−8.3	Trp420, Phe421, Pro173, Leu436, Ala424		π−σ; Leu436 π-alkyl; Pro173, Ala424 π−π T-shaped; Phe421 amide-πstacked; Trp420, Phe421
**3h**	−9.2	Trp420, Phe421, Pro173, Leu436, Ala440		π−σ; Leu436 π−π stacked; Trp420 π-alkyl; Pro173, Leu436, Ala440 amide-πstacked; Trp420, Phe421
**3i**	−9.6	Ser112, Trp420, Phe421, Pro173, Leu436, Ala440	Conventional-H; Gly116	π−σ; Leu436 π−π stacked; Trp420 π−π T-shaped; Phe421 π-alkyl; Pro173, Leu436 amide-πstacked; Trp420, Phe421
**3j**	−8.8	Ser169, Pro173, Phe421, Ala424, Ala440, Trp420, Leu436	Conventional-H; Ser169	π-alkyl; Pro173, Phe421, Ala424, Ala440 amide-πstacked; Trp420, Leu436
**3k**	−9.1	Ser169, His192, Trp420, Phe421, Pro173, Leu436, Ala424	Conventional-H; Ser169 Carbon-H; His192	π−σ; Leu436 π−π stacked; Trp420 π−π T-shaped; Phe421 π-alkyl; Pro173, Ala424
**3l**	−8.8	Asn391, Val465, Tyr325, Gly462, Ile329, Pro463, Leu390, Leu387, Ala458	Carbon-H; Asn391	π−σ; Val465 π−π stacked; Tur325 alkyl; Pro463, Leu390 π-alkyl; Ala458, Ile329, Leu387, Pro463 amide-πstacked; Gly462
**3m**	−8.5	Ser169, Ser112, Trp420, Phe421, Pro173, Leu436, Ala424, Met174	Conventional-H; Ser169 Carbon-H; Ser112	π−σ; Leu436 π−π T-shaped; Phe421 π-alkyl; Pro173, Ala424 alkyl; Leu436, Ala440, Met174 amide-πstacked; Trp420, Phe421
**3n**	−7.7	Pro173, Met174, Ala424, leu436		π−σ; Pro173, Met174 alkyl; Met174, Leu436 π-alkyl; Ala424, Pro173
**3o**	−8.3	Trp420, Phe421, Pro173, Leu436, Ala424, Ala440		π−σ; Leu436 π−π stacked; Trp420 alkyl; Ala424 π-alkyl; Pro173, Phe421, Ala440

**Table 3:** Molecular docking of the 2-phenyl-3H-quinazolin-4-one derivatives (**3a**–**3o**) with α-amylase enzyme: binding energies, interacting amino acid residues of α-amylase enzyme, and polar and hydrophobic interactions.

Compounds	Binding Energy (kcal/mol)	Amino Acid Residues	Polar Interactions	Hydrophobic Interactions
**3a**	−7.7	Trp59, Tyr62		π−π stacked; Trp59, Tyr62
**3b**	−7.7	Trp59, Tyr62		π−π stacked; Trp59, Tyr62
**3c**	−8.0	Trp59, Tyr62, His101, Asp197	Conventional-H; His101, Asp197	π−π stacked; Trp59, Tyr62
**3d**	−8.2	Trp59, Tyr62, Asp300		π−π stacked; Trp59, Tyr62 π−anion; Asp300
**3e**	−7.4	Trp58, Trp59, Tyr62, Leu162, Asp300		π−π stacked; Trp59 π−anion; Asp300 π−π T-shaped; Trp58, Tyr62 alkyl; Leu162
**3f**	−7.3	Trp59, Tyr62, His299	Carbon-H; Ser112	π−π stacked; Trp59, Tyr62 π−alkyl; His299
**3g**	−7.6	Trp59, Tyr62, Adp197, Asp300		π−π stacked; Trp59 π−anion; Asp197, Asp300 π−π T-shaped; Tyr62
**3h**	−8.2	Trp59, Tyr62, Asp300		π−π stacked; Trp59, Tyr62 π−anion; Asp300
**3i**	−8.1	Trp59, Tyr62, Arg195, Asp300	Conventional-H; Arg195	π−π stacked; Trp59 π−anion; Asp300 π−π T-shaped; Tyr62
**3j**	−7.5	Trp59, Tyr62, His299, Asp300		π−π stacked; Trp59 π−anion; Asp300 π−π T-shaped; Tyr62 π−alkyl; His299
**3k**	−7.8	Trp59, Tyr62, Arg195, Glu233, Asp300	Conventional-H; Arg195	π−π stacked; Trp59 π−anion; Asp300 π−π T-shaped; Tyr62 Halogen; Glu233
**3l**	−7.3	Trp59, Glu233, His299, Asp300	Carbon-H; Glu233	π−π stacked; Trp59 π−anion; Asp300 π−alkyl; His299
**3m**	−7.3	Trp59, Tyr62, Leu165, Asp300, His305		π−π stacked; Trp59 π−anion; Asp300 π−π T-shaped; Tyr62 alkyl; Leu165 π−alkyl; Trp59, His305
**3n**	−6.8	Trp59, Tyr62, His305		π−π stacked; Trp59, Try62 π−alkyl; His305
**3o**	−7.2	Trp59, Tyr62, His299, Asp300	Carbon-H His299	π−π stacked; Trp59 π−anion; Asp300 π−π T-shaped; Trp59 π−alkyl; Trp59

**Table 4:** Molecular docking of the 2-phenyl-3*H*-quinazolin-4-one derivatives (**3a**–**3o**) with α-amylase enzyme: binding energies, interacting amino acid residues of α-glucosidase enzyme, and polar and hydrophobic interactions.

Compounds	Binding Energy (kcal/mol)	Amino Acid Residues	Polar Interactions	Hydrophobic Interactions
**3a**	−7.6	Arg725, Glu748, Ala749, His708	Conventional-H; Arg725	π−anion; Glu748 π−alkyl; Ala749 π−σ; His708
**3b**	−7.4	Arg275, Ala93, Pro125, Val321, Asp91	Conventional-H; Arg275, Ala93	π−σ; Pro125 π−alkyl; Val321, Arg275 π−anion; Asp91
**3c**	−7.1	Asp518, Asp616, Trp376, Phe649, Met519		π−anion; Asp518, Asp616 π−π stacked; Trp376, Phe649 π−sulfur; Met519
**3d**	−7.0	His742, Leu756, Val740, Trp804	Conventional-H; His742	π−alkyl; Leu756, π−π T-shaped; Trp804
**3e**	−7.0	Val867, Leu868, Met363, Arg594, Arg608, His717, Glu869	Conventional-H; Val867, Leu868 Carbon-H; Arg608	π−anion; Glu869 π−donor H; Met363 π−π T-shaped; His717 alkyl; His717 π−alkyl; Arg594, Leu868
**3f**	−6.9	His742, Leu756, Val740, Trp804	Conventional-H; His742	π−alkyl; Leu756, Val740 π−π T-shaped; Trp804
**3g**	−7.2	Lys760, Val755, His742, Val763, Thr764, Trp804	Conventional-H; His742	π−alkyl; Lys760, Val755 π−σ; Val 763
**3h**	−7.9	Ala93, Pro125, Trp126, Asp91, Gly123, Arg331, Val321, Arg275	Conventional-H; Arg275, Gly123 π-donor H; Trp126	π−alkyl; Ala93, Pro125, Val321 π−anion; Asp91, Arg331
**3i**	−7.1	Ile780, Leu701, Lys697, Thr700	Conventional-H; Ile780	π−alkyl; Lys697, Leu701 π−sigma; Thr700
**3j**	−7.2	Asp91, Cys127, Trp126, Trp126, Val321, Pro125	Conventional-H; Asp91, Cys127 π-donor H; Trp126	π−π; Trp126 alkyl; Val321 π−alkyl; Pro125
**3k**	−7.3	Arg275, Val321, Asp91, Pro125, Trp126	π-donor H; Trp126	π−anion; Asp91 π−σ; Pro125 π−alkyl; Arg275, Val321
**3l**	−6.4	Arg837, Trp859, Asp861	Conventional-H; Arg837, Trp859 Carbon-H; Asp861 π-donor H; Arg837	π−alkyl-Arg837
**3m**	−6.9	Gly123, Asp91, Cys127	Conventional-H; Gly123, Asp91, Cys127	π−anion; Asp91
**3n**	−6.2	His562, Thr469, Gly473, Pro475, Asn492, Phe467, Gly465, Leu462	Conventional-H; Asn492, Phe467, Gly465 Carbon-H; Gly473	π−sigma; Thr469 π−π T-shaped; His562 alkyl; Pro475, Leu462, Phe467
**3o**	−6.7	Met408, Asp406, Ser379, Leu405, Leu677, Asn417	Conventional-H; Met408, Asp406, Ser379, Leu405, Asn417	π−anion; Asp 406 π−σ; Leu 677; π-alkyl; Leu 677

**Table 5:** ADMET prediction profiles of the 2-phenyl-3*H*-quinazolin-4-one derivatives (**3a**–**3o**).

ADMET Predict Profile	3a	3b	3c	3d	3e	3f	3g	3h	3i	3j	3k	3l	3m	3n	3o
Ames mutagenesis	+	+	+	+	+	+	+	+	+	+	+	+	+	+	+
Mouse carcinogenicity	+	+	+	+	−	−	+	+	+	+	−	+	+	+	+
Rat carcinogenicity	+	+	+	+	−	+	+	+	+	+	+	+	+	+	+
Rodent carcinogenicity	+	+	+	+	−	+	+	+	+	+	+	+	+	+	+
Micronucleus	+	+	+	+	+	+	+	+	+	+	+	+	+	+	+
Reproductive toxicity	-	-	+	+	+	+	+	+	+	+	+	+	+	+	-
Mitochondrial toxicity	+	+	+	+	+	+	+	+	+	+	+	+	+	+	+
Hemolytic toxicity	−	−	−	−	−	−	−	−	−	−	−	−	−	−	−
Repeated dose toxicity	+	+	+	+	+	+	+	+	+	+	+	+	+	+	+
Acute oral toxicity	−	+	−	+	+	+	+	+	+	+	+	+	+	+	−
Respiratory toxicity	+	+	+	+	+	+	+	+	+	+	+	+	+	+	+
Nephrotoxicity	+	+	+	+	-	+	+	+	+	+	+	−	−	−	+
Eye corrosion	−	−	−	−	−	−	−	−	−	−	−	−	−	−	−
Eye irritation	+	+	+	+	−	−	−	+	−	−	−	−	−	−	+
Skin corrosion	−	−	−	−	−	−	−	−	−	−	−	−	−	−	−
Skin irritation	−	−	−	−	−	−	−	−	−	−	−	−	−	−	−
Skin sensitization	−	−	−	−	−	−	−	−	−	−	−	−	−	−	−
Acute dermal toxicity	+	+	+	+	+	+	+	+	+	+	+	+	+	−	+
Crustacean toxicity	−	+	+	-	-	+	+	+	+	+	+	−	−	−	−
Fish toxicity	−	+	+	+	+	+	+	+	+	+	+	−	−	−	−
Honey bee toxicity	−	−	−	−	−	−	−	−	−	−	−	−	−	−	−
Biodegradability	−	−	−	−	−	−	−	−	−	−	−	−	−	−	−
CYP1A2 inhibitor	+	+	+	+	+	+	+	+	+	+	+	+	+	+	+
CYP3A4 inhibitor	−	−	−	−	−	−	−	−	−	−	−	−	−	−	−
CYP2B6 inhibitor	−	−	−	−	−	−	−	−	−	−	−	−	−	−	−
CYP2C9 inhibitor	−	−	−	−	−	−	+	−	−	−	−	−	−	−	−
CYP2C19 inhibitor	−	−	−	−	−	−	+	−	−	+	−	−	−	−	−
CYP2D6 inhibitor	−	−	−	−	−	−	−	−	−	−	−	−	−	−	−
CYP1A2 substrate	−	−	−	−	+	+	+	+	+	+	+	+	+	+	−
CYP3A4 substrate	−	−	−	−	−	+	+	+	+	+	+	−	−	−	−
CYP2B6 substrate	−	−	−	−	−	−	−	+	−	+	+	−	−	−	−
CYP2C9 substrate	−	−	−	−	−	−	+	+	+	−	−	−	−	−	−
CYP2C19 substrate	−	−	−	−	+	+	+	+	+	+	+	−	−	−	−
CYP2D6 substrate	−	−	−	−	−	−	−	−	−	−	−	−	−	−	−
OATP1B1 inhibitor	+	+	+	+	+	+	+	+	+	+	+	+	+	+	+
OATP1B3 inhibitor	+	+	+	+	+	+	+	+	+	+	+	+	+	+	+
OATP2B1 inhibitor	−	−	−	−	−	−	−	−	−	−	−	−	−	−	−
OCT1 inhibitor	−	−	−	−	−	−	−	−	−	+	+	−	−	−	−
OCT2 inhibitor	−	−	−	−	−	−	−	−	−	−	−	−	−	−	−
PPARγ	−	−	−	−	−	−	−	−	−	−	−	−	−	−	−
Lipinski Rule	+	+	+	+	+	+	+	+	+	+	+	+	+	+	+

In the ADMET profile table, plus (+) sign denotes positive, active, or present (toxic, mutagenic, or an inhibitor), while a minus (−) sign denotes negative, inactive, or absent (non-toxic or safe).

## Data Availability

The original contributions presented in this study are included in the article/Supplementary Materials. Further inquiries can be directed to the corresponding authors.

## Supplementary Materials

The following supporting information can be downloaded at: https://www.mdpi.com/article/10.3390/pharmaceutics18081013/s1, Figure S01: ^1^H NMR of 2-phenyl-*3H*-quinazolin-4-one (3(a)); Figure S02: ^1^H NMR of 2-(2-hydroxyphenyl)-*3H*-quinazolin-4-one (3(b)); Figure S03: ^1^H NMR of 2-(3-hydroxyphenyl)-*3H*-quinazolin-4-one (3(c)); Figure S04: ^1^H NMR of 2-(4-hydroxyphenyl)-*3H*-quinazolin-4-one (3(d)); Figure S05: ^1^H NMR of 2-(2-methoxyphenyl)-*3H*-quinazolin-4-one (3(e)); Figure S06: ^1^H NMR of 2-(4-methoxyphenyl)-*3H*-quinazolin-4-one (3(f)); Figure S07: ^1^H NMR of 2-(2-nitrophenyl)-*3H*-quinazolin-4-one (3(g)); Figure S08: ^1^H NMR of 2-(3-nitrophenyl)-*3H*-quinazolin-4-one (3(h)); Figure S09:^1^H NMR of 2-(4-nitrophenyl)-*3H*-quinazolin-4-one (3(i)); Figure S10: ^1^H NMR of 2-(4-bromophenyl)-*3H*-quinazolin-4-one (3(j)); Figure S11: ^1^H NMR of 2-(4-fluorophenyl)-*3H*-quinazolin-4-one (3(k)); Figure S12: ^1^H NMR of 2-(2,4-dimethoxyphenyl)-*3H*-quinazolin-4-one (3(l)); Figure S13: ^1^H NMR of 2-(2,5-dimethoxyphenyl)-*3H*-quinazolin-4-one (3(m)); Figure S14: ^1^H NMR of 2-(3,4,5-trimethoxyphenyl)-*3H*-quinazolin-4-one (3(n)); Figure S15: ^1^H NMR of 2-(4-hydroxy-3-methoxyphenyl)-*3H*-quinazolin-4-one (3(o)); Figure S16: ^13^C NMR of 2-phenyl-*3H*-quinazolin-4-one (3(a)); Figure S17: ^13^C NMR of 2-(2-hydroxyphenyl)-*3H*-quinazolin-4-one (3(b)); Figure S18: ^13^C NMR of 2-(3-hydroxyphenyl)-*3H*-quinazolin-4-one (3(c)); Figure S19: ^13^C NMR of 2-(4-hydroxyphenyl)-*3H*-quinazolin-4-one (3(d)); Figure S20: ^13^C NMR of 2-(2-methoxyphenyl)-*3H*-quinazolin-4-one (3(e)); Figure S21:^13^C NMR of 2-(4-methoxyphenyl)-*3H*-quinazolin-4-one (3(f)); Figure S22: ^13^C NMR of 2-(2-nitrophenyl)-*3H*-quinazolin-4-one (3(g)); Figure S23: ^13^C NMR of 2-(3-nitrophenyl)-*3H*-quinazolin-4-one (3(h)); Figure S24: ^13^C NMR of 2-(4-nitrophenyl)-*3H*-quinazolin-4-one (3(i)); Figure S25: ^13^C NMR of 2-(4-bromophenyl)-*3H*-quinazolin-4-one (3(j)); Figure S26: ^13^C NMR of 2-(4-fluorophenyl)-*3H*-quinazolin-4-one (3(k)); Figure S27: ^13^C NMR of 2-(2,4-dimethoxyphenyl)-*3H*-quinazolin-4-one (3(l)); Figure S28: ^13^C NMR of 2-(2,5-dimethoxyphenyl)-*3H*-quinazolin-4-one (3(m)); Figure S29:^13^C NMR of 2-(3,4,5-trimethoxyphenyl)-*3H*-quinazolin-4-one (3(n)); Figure S30: ^13^CNMR of 2-(4-hydroxy-3-methoxyphenyl)-*3H*-quinazolin-4-one (3(o)); Figure S31: Mass spectrum of 2-phenyl-*3H*-quinazolin-4-one (3(a)); Figure S32: Mass spectrum of 2-(2-hydroxyphenyl)-*3H*-quinazolin-4-one (3(b)); Figure S33: Mass spectrum of 2-(3-hydroxyphenyl)-*3H*-quinazolin-4-one (3(c)); Figure S34: Mass spectrum of 2-(4-hydroxyphenyl)-*3H*-quinazolin-4-one (3(d)); Figure S35: Mass spectrum of 2-(2-methoxyphenyl)-*3H*-quinazolin-4-one (3(e)); Figure S36: Mass spectrum of 2-(4-methoxyphenyl)-*3H*-quinazolin-4-one (3(f)); Figure S37: Mass spectrum of 2-(2-nitrophenyl)-*3H*-quinazolin-4-one (3(g)); Figure S38: Mass spectrum of 2-(3-nitrophenyl)-*3H*-quinazolin-4-one (3(h)); Figure S39: Mass spectrum of 2-(4-nitrophenyl)-*3H*-quinazolin-4-one (3(i)); Figure S40: Mass spectrum of 2-(4-bromophenyl)-*3H*-quinazolin-4-one (3(j)); Figure S41: Mass spectrum of 2-(4-fluorophenyl)-*3H*-quinazolin-4-one (3(k)); Figure S42: Mass spectrum of 2-(2,4-dimethoxyphenyl)-*3H*-quinazolin-4-one (3(l)); Figure S43: Mass spectrum of 2-(2,5-dimethoxyphenyl)-*3H*-quinazolin-4-one (3(m)); Figure S44: Mass spectrum of 2-(3,4,5-trimethoxyphenyl)-*3H*-quinazolin-4-one (3(n)); Figure S45: Mass spectrum of 2-(4-hydroxy-3-methoxyphenyl)-*3H*-quinazolin-4-one (3(o)).

## References

[1] Papatheodorou K., Banach M., Bekiari E., Rizzo M., Edmonds M. (2018). Complications of Diabetes 2017. J. Diabetes Res..

[2] Alam U., Asghar O., Azmi S., Malik R.A. (2014). General Aspects of Diabetes Mellitus. Handbook of Clinical Neurology.

[3] Atkinson M.A., Eisenbarth G.S., Michels A.W. (2014). Type 1 Diabetes. Lancet.

[4] Olokoba A.B., Obateru O.A., Olokoba L.B. (2012). Type 2 Diabetes Mellitus: A Review of Current Trends. Oman Med. J..

[5] Paul S., Majumdar M. (2020). In-Vitro Antidiabetic Propensities, Phytochemical Analysis, and Mechanism of Action of Commercial Antidiabetic Polyherbal Formulation “Mehon”. Proceedings.

[6] Madiwalar V.S., Dwivedi P.S.R., Patil A., Gaonkar S.M.N., Kumbhar V.J., Khanal P., Patil B.M. (2022). Ficus Benghalensis Promotes the Glucose Uptake- Evidence with in Silico and in Vitro. J. Diabetes Metab. Disord..

[7] Jayasinghe R.N., Padukkage Dona N.T., Lorensu Hewage V.C., Egodawaththa N.M., Nesnas N., Gunaratna M.J. (2025). Evaluation of In Silico and In Vitro Antidiabetic Properties of 4-(Hydroxysubstituted Arylidene)-2-Phenyloxazol-5(4 *H* )-One Derivatives. ACS Omega.

[8] Özcan S., Johnston M. (1999). Function and Regulation of Yeast Hexose Transporters. Microbiol. Mol. Biol. Rev..

[9] Wu C.-H., Huang C.-H., Chung M.-C., Chang S.-H., Tsai G.-J. (2021). Exploration of Hypoglycemic Activity of Saccharomyces Pastorianus Extract and Evaluation of the Molecular Mechanisms. Molecules.

[10] Tchamani Piame L., Yako Y.Y. (2026). A Systematic Review of the Effects of Saccharomyces Boulardii on Diabetes Mellitus in Experimental Mice Models. Encyclopedia.

[11] Souza P.M.D., Magalhães P.D.O.E. (2010). Application of Microbial α-Amylase in Industry—A Review. Braz. J. Microbiol..

[12] Dirir A.M., Daou M., Yousef A.F., Yousef L.F. (2022). A Review of Alpha-Glucosidase Inhibitors from Plants as Potential Candidates for the Treatment of Type-2 Diabetes. Phytochem. Rev..

[13] Badolato M., Aiello F. (2016). 2,3-Dihydroquinazolin-4(1H)-One as a Privileged Scaffold in Drug Design. RSC Adv..

[14] Moheb M., Iraji A., Dastyafteh N., Khalili Ghomi M., Noori M., Mojtabavi S., Faramarzi M.A., Rasekh F., Larijani B., Zomorodian K. (2022). Synthesis and Bioactivities Evaluation of Quinazolin-4(3H)-One Derivatives as α-Glucosidase Inhibitors. BMC Chem..

[15] Bisht A.S., Negi J.S., Sharma D.K. (2020). Chemistry and Activity of Quinazoline Moiety: A Systematic Review Study. Int. J. Pharm. Chem. Anal..

[16] Baghershahi P., Dastyafteh N., Halimi M., Mohammadi-Khanaposhtani M., Ghafouri S.N., Noori M., Ghasemi F., Mojtabavi S., Faramarzi M.A., Torabi M. (2026). Design, Synthesis, in Vitro, and in Silico Studies on Promising α-Glucosidase Inhibitors Based on a Quinazolinone-Thiophene Scaffold. RSC Adv..

[17] Dhameja M., Gupta P. (2019). Synthetic Heterocyclic Candidates as Promising α-Glucosidase Inhibitors: An Overview. Eur. J. Med. Chem..

[18] Modh P.G., Patel L.J. (2011). In Vitro Screening on Alpha Amylase and Alpha Glucosidase Inhibitory Activities of Some Novel Quinazolinone Derivatives. Int. J. Pharm. Sci. Res..

[19] Pedrood K., Sherafati M., Mohammadi-Khanaposhtani M., Asgari M.S., Hosseini S., Rastegar H., Larijani B., Mahdavi M., Taslimi P., Erden Y. (2021). Design, Synthesis, Characterization, Enzymatic Inhibition Evaluations, and Docking Study of Novel Quinazolinone Derivatives. Int. J. Biol. Macromol..

[20] Mravljak J., Slavec L., Hrast M., Sova M. (2021). Synthesis and Evaluation of Antioxidant Properties of 2-Substituted Quinazolin-4(3H)-Ones. Molecules.

[21] Asif M. (2014). Chemical Characteristics, Synthetic Methods, and Biological Potential of Quinazoline and Quinazolinone Derivatives. Int. J. Med. Chem..

[22] Yavari A., Mohammadi-Khanaposhtani M., Moradi S., Bahadorikhalili S., Pourbagher R., Jafari N., Faramarzi M.A., Zabihi E., Mahdavi M., Biglar M. (2021). α-Glucosidase and α-Amylase Inhibition, Molecular Modeling and Pharmacokinetic Studies of New Quinazolinone-1,2,3-Triazole-Acetamide Derivatives. Med. Chem. Res..

[23] Inzucchi S.E., Bergenstal R.M., Buse J.B., Diamant M., Ferrannini E., Nauck M., Peters A.L., Tsapas A., Wender R., Matthews D.R. (2012). Management of Hyperglycemia in Type 2 Diabetes: A Patient-Centered Approach: Position Statement of the American Diabetes Association (ADA) and the European Association for the Study of Diabetes (EASD). Diabetes Spectr..

[24] Wang G.-W., Miao C.-B., Kang H. (2006). Benign and Efficient Synthesis of 2-Substituted 4(3 *H* )-Quinazolinones Mediated by Iron(III) Chloride Hexahydrate in Refluxing Water. Bull. Chem. Soc. Jpn..

[25] Rudolph J., Esler W.P., O’connor S., Coish P.D.G., Wickens P.L., Brands M., Bierer D.E., Bloomquist B.T., Bondar G., Chen L. (2007). Quinazolinone Derivatives as Orally Available Ghrelin Receptor Antagonists for the Treatment of Diabetes and Obesity. J. Med. Chem..

[26] Ali H., Jan A., Ali G., Ali S.S., Rahim A., Nahla U.A. (2023). Assessment of Antidiabetic, Hepatoprotective, and Analgesic Effects of Quinazolinone Derivative, (E)-1-Benzoyl-3-((4-(Dimethylamino) Benzylidene) Amino)-2-(4-(Dimethylamino) Phenyl)-2,3 Dihydroquinazoline-4(1h)-One, in Diabetes Induced Mice Model. ACS Omega.

[27] Tokalı F.S., Taslimi P., Tuzun B., Karakuş A., Sadeghian N., Gulçin İ. (2023). Novel Quinazolinone Derivatives: Potential Synthetic Analogs for the Treatment of Glaucoma, Alzheimer’s Disease and Diabetes Mellitus. Chem. Biodivers..

[28] Srinivasa M.G., Paithankar J.G., Saheb Birangal S.R., Pai A., Pai V., Deshpande S.N., Revanasiddappa B.C. (2023). Novel Hybrids of Thiazolidinedione-1,3,4-Oxadiazole Derivatives: Synthesis, Molecular Docking, MD Simulations, ADMET Study, In Vitro, and In Vivo Anti-Diabetic Assessment. RSC Adv..

[29] Shankara S.D., Isloor A.M., Kudva A.K., Raghu S.V., Jayaswamy P.K., Venugopal P.P., Shetty P., Chakraborty D. (2022). 2,5-Bis(2,2,2-trifluoroethoxy)phenyl-tethered 1,3,4-Oxadiazoles Derivatives: Synthesis, In Silico Studies, and Biological Assessment as Potential Candidates for Anti-Cancer and Anti-Diabetic Agent. Molecules.

[30] Khan S.A., Ali M., Latif A., Ahmad M., Khan A., Al-Harrasi A. (2022). Mercaptobenzimidazole-Based 1,3-Thaizolidin-4-Ones as Antidiabetic Agents: Synthesis, In Vitro α-Glucosidase Inhibition Activity, and Molecular Docking Studies. ACS Omega.

[31] UniProt. Uniprot. https://www.uniprot.org/.

[32] SWISS-MODEL. swissmodel.expasy.org. https://swissmodel.expasy.org.

[33] RCSB PDB. Rcsb.org. https://www.rcsb.org/.

[34] PubChem Nih.gov. https://pubchem.ncbi.nlm.nih.gov/.

[35] Dallakyan S., Olson A.J., Hempel J.E., Williams C.H., Hong C.C. (2015). Small-Molecule Library Screening by Docking with PyRx. Chemical Biology.

[36] Sarkar M., Nath A., Kumer A., Mallik C., Akter F., Moniruzzaman M., Ali M.A. (2021). Synthesis, Molecular Docking Screening, ADMET and Dynamics Studies of Synthesized 4-(4-Methoxyphenyl)-8-Methyl-3,4,5,6,7,8-Hexahydroquinazolin-2(1H)-One and Quinazolinone Derivatives. J. Mol. Struct..

[37] admetSAR: Index. Edu.cn. https://lmmd.ecust.edu.cn/admetsar3/.

[38] Nazir M., Abbasi M.A., Rehman A.U., Siddiqui S.Z., Khan K.M., Kanwal, Salar U., Shahid M., Ashraf M., Arif Lodhi M. (2018). New Indole Based Hybrid Oxadiazole Scaffolds with N-Substituted Acetamides: As Potent Anti-Diabetic Agents. Bioorganic Chem..

[39] Wu X.-Q., Wang J., Lü Z.-R., Tang H.-M., Park D., Oh S.-H., Bhak J., Shi L., Park Y.-D., Zou F. (2010). Alpha-Glucosidase Folding During Urea Denaturation: Enzyme Kinetics and Computational Prediction. Appl. Biochem. Biotechnol..

[40] Kumar V., Prakash O., Kumar S., Narwal S. (2011). α-Glucosidase Inhibitors from Plants: A Natural Approach to Treat Diabetes. Pharmacogn. Rev..

[41] Wei M., Chai W.-M., Wang R., Yang Q., Deng Z., Peng Y. (2017). Quinazolinone Derivatives: Synthesis and Comparison of Inhibitory Mechanisms on α-Glucosidase. Bioorg. Med. Chem..

[42] Aloysius M.T., Felekkis K., Petrou C., Egwim E.C., Andreou E. (2024). In Vitro Glucose Uptake in Yeast Cell Facilitated by Abelmoschus Esculentus L. (Okra Seed) for Management of Type 2 Diabetes. Preprints.

[43] Chiodi D., Ishihara Y. (2024). The Role of the Methoxy Group in Approved Drugs. Eur. J. Med. Chem..

[44] Mazumdar S., Marar T., Patki J., Devarajan S., Zambare V., Swami D. (2020). In Silico and in Vitro Analysis Reveal Multi-Target Anti-Hyperglycaemic Properties of Gedunin, a Limonoid from Neem (Azadirachta Indica). Clin. Phytoscience.

