# Supplementary material for: Synthesis and Anti-Diabetic Evaluation of 2-Phenyl-3H-quinazolin-4-one Derivatives
Source: Pharmaceutics. 2026 Aug 16;18(8):1013. doi: 10.3390/pharmaceutics18081013 (PMC13516565; doi:10.3390/pharmaceutics18081013)
Supplement: Supplementary file 1 [file pharmaceutics-18-01013-s001.zip › pharmaceutics-4449594-supplementary.pdf]

Synthesis and anti-diabetic evaluation of 2-phenyl-3*H*-quinazolin-4-one derivatives

Pathirana K. P. D. H. [1], D. A. Upeka Chathurangani [1], Julian Vlad [2], Dissanayake D. M. W. S. [1], Alwis Y. V. [1], G. Mashooda Jayah [1], Pathirana K. P. S. S. [3], Dinusha N. Udukala [1], Nishal M. Egodawaththa [2], Jason P. Farrah [4], Nasri Nesnas[2] and Medha J. Gunaratna [3]*

1 College of Chemical Sciences, Institute of Chemistry Ceylon, , Rajagiriya, 10107, Sri Lanka; stu215821@student.ichemc.edu.lk (P.K.P.D.H.); upeka@ichemc.edu.lk (D.A.U.C.); warshadissanayake0@gmail.com (D.D.M.W.S.); vindulaalwis95@gmail.com (A.Y.V.); stu215775@student.ichemc.edu.lk (G.M.J.); dinusha@ichemc.edu.lk (D.N.U.)
2 Department of Chemistry and Chemical Engineering, Florida Institute of Technology, 150 West University Boulevard, Melbourne, FL 32901, USA; jvlad2024@my.fit.edu (J.V.); enishalmaduj2020@my.fit.edu (N.M.E.); nesnas@fit.edu (N.N.)
3 Department of Chemistry, Faculty of Science, University of Kelaniya, Kelaniya, Sri Lanka; swapnapathirana@gmail.com
4 Body Revolution Aesthetics & Wellness, 1211 48th Avenue, Myrtle Beach, SC 29577, USA; drfarrah@bodyrevolutionwellness.com
* Correspondence: medhagunaratna@kln.ac.lk

SUPPORTING INFORMATION

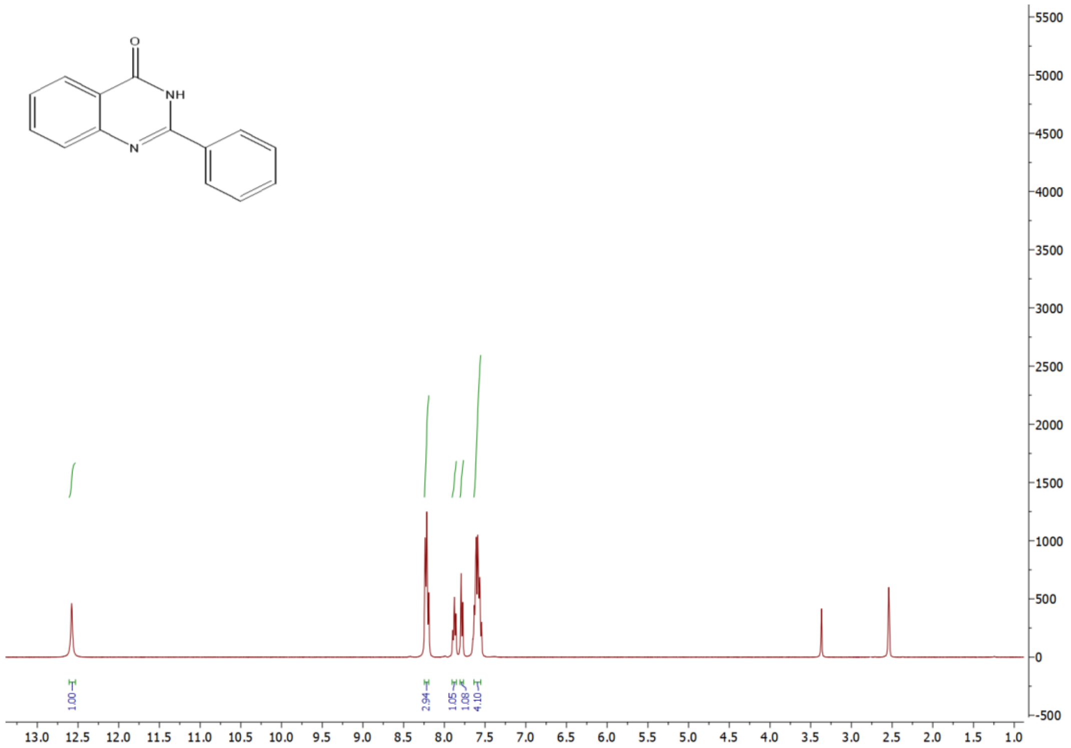

Figure S01: ^{1}H NMR of 3(**a**)

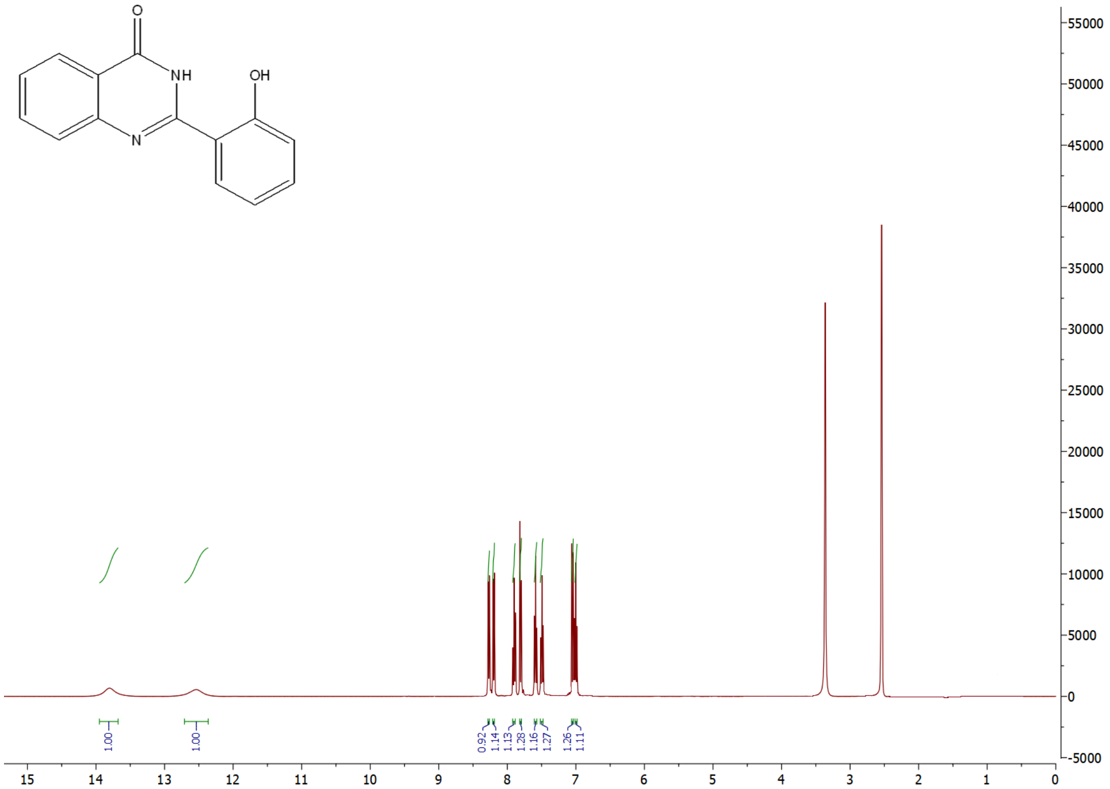

Figure S02: ^{1}H NMR of 3(**b**)

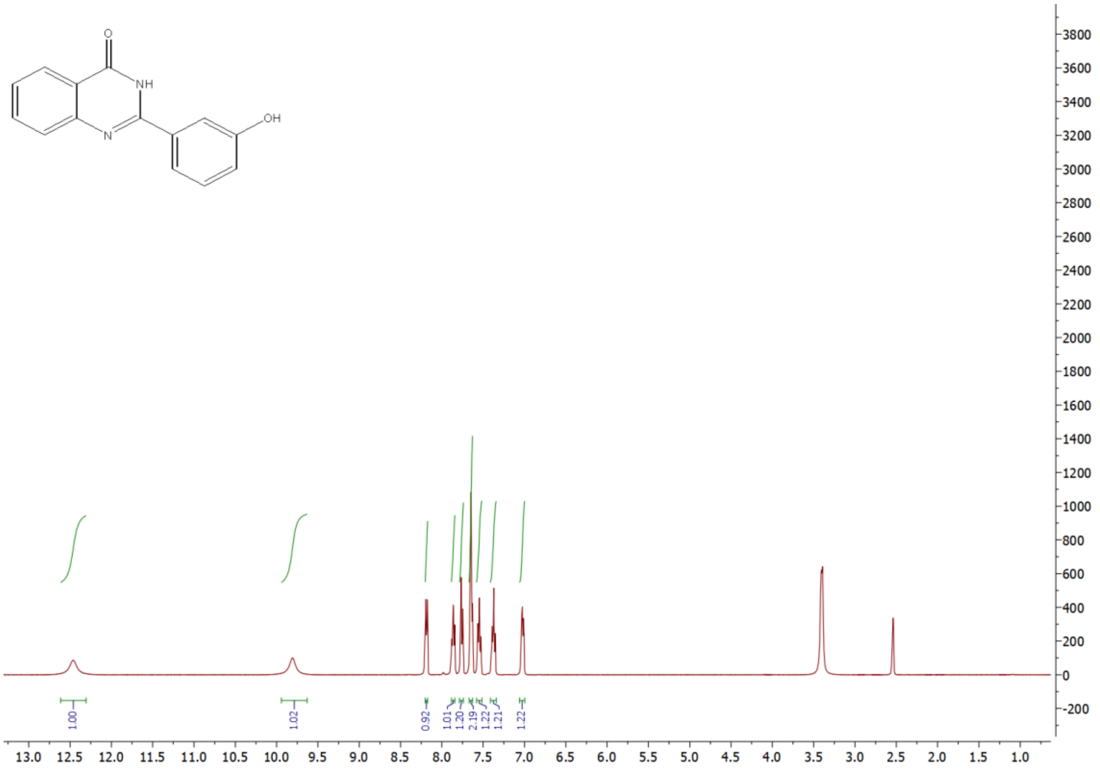

Figure S03: ^{1}H NMR of 3(**c**)

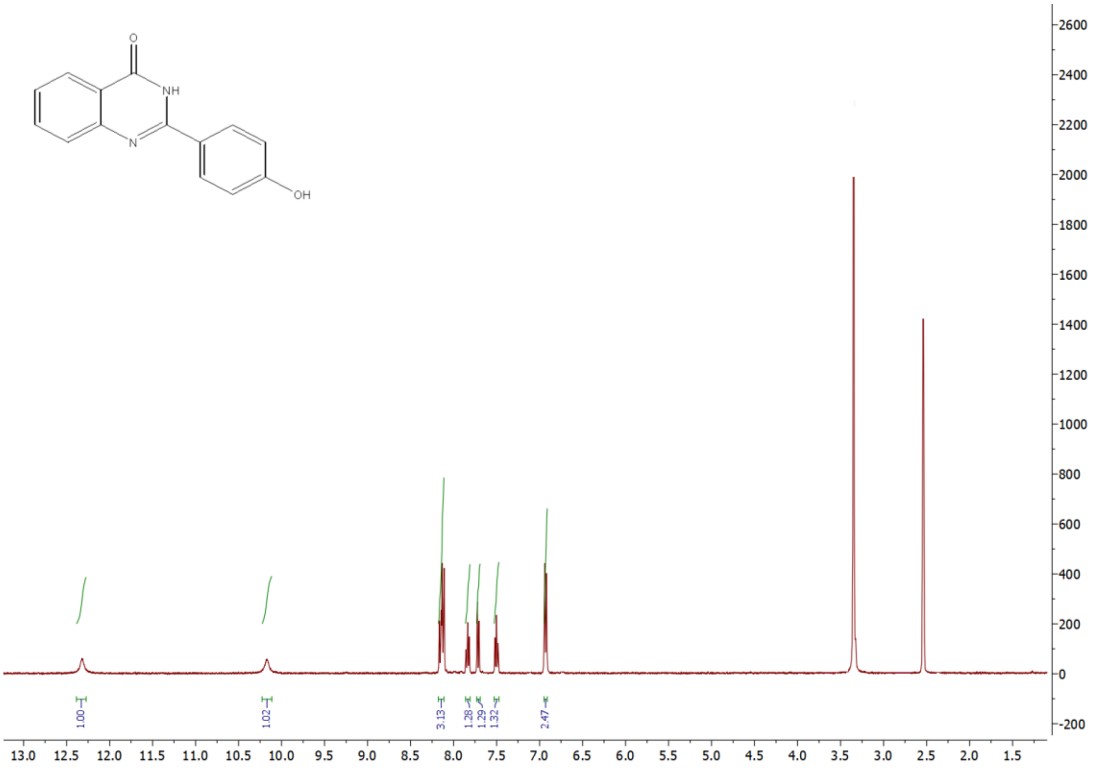

Figure S04: ^{1}H NMR of 3(**d**)

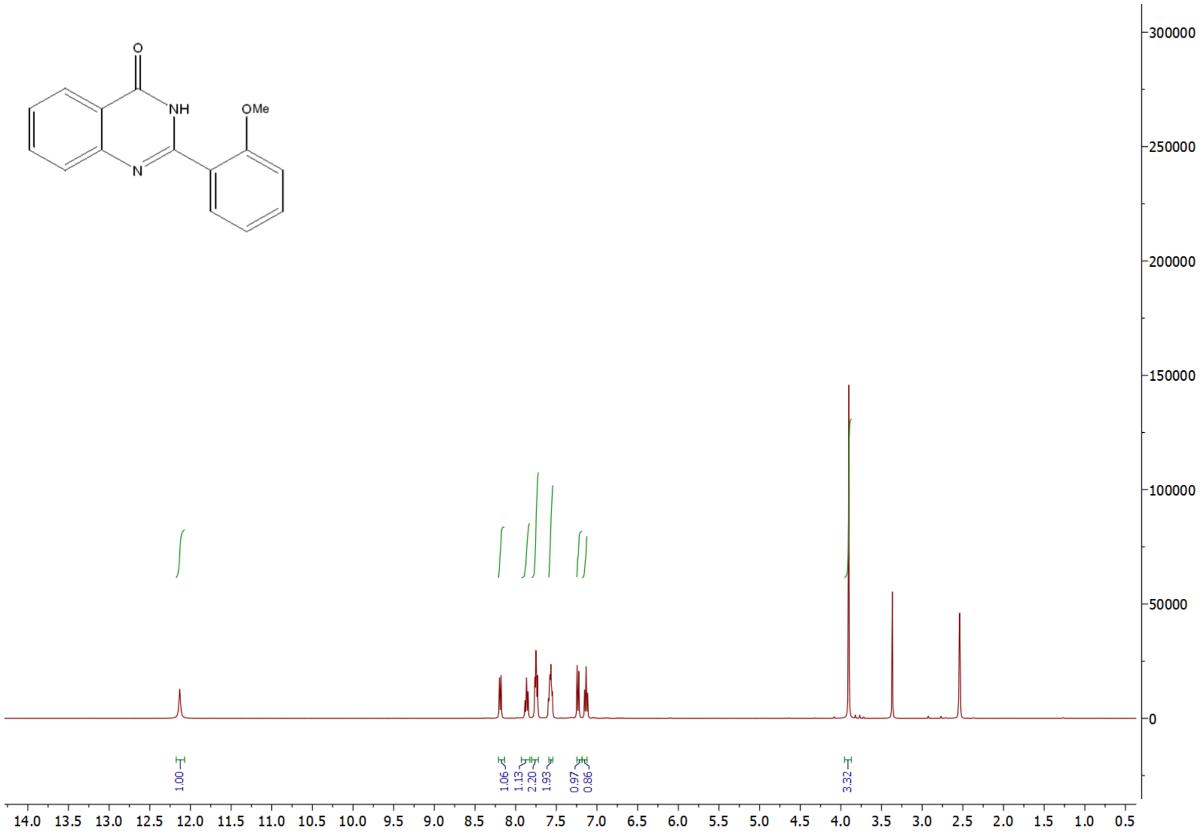

Figure S05: ^{1}H NMR of 3(**e**)

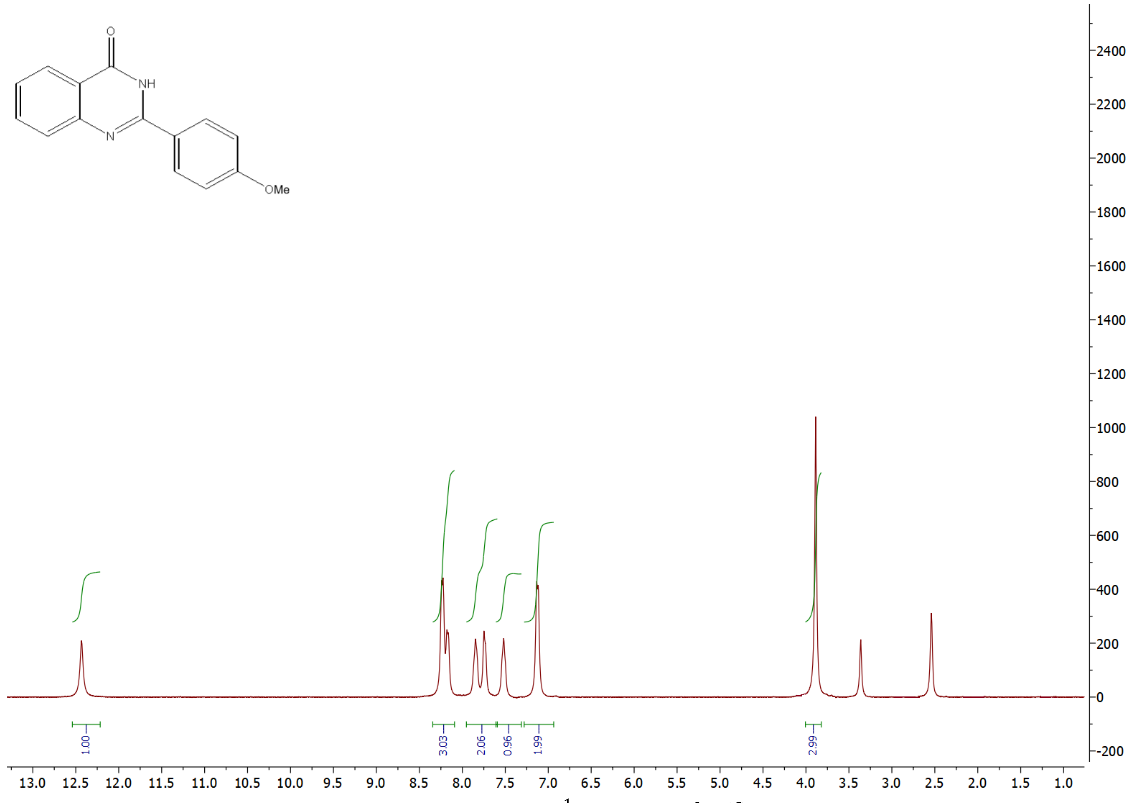

Figure S06: ^{1}H NMR of 3(**f**)

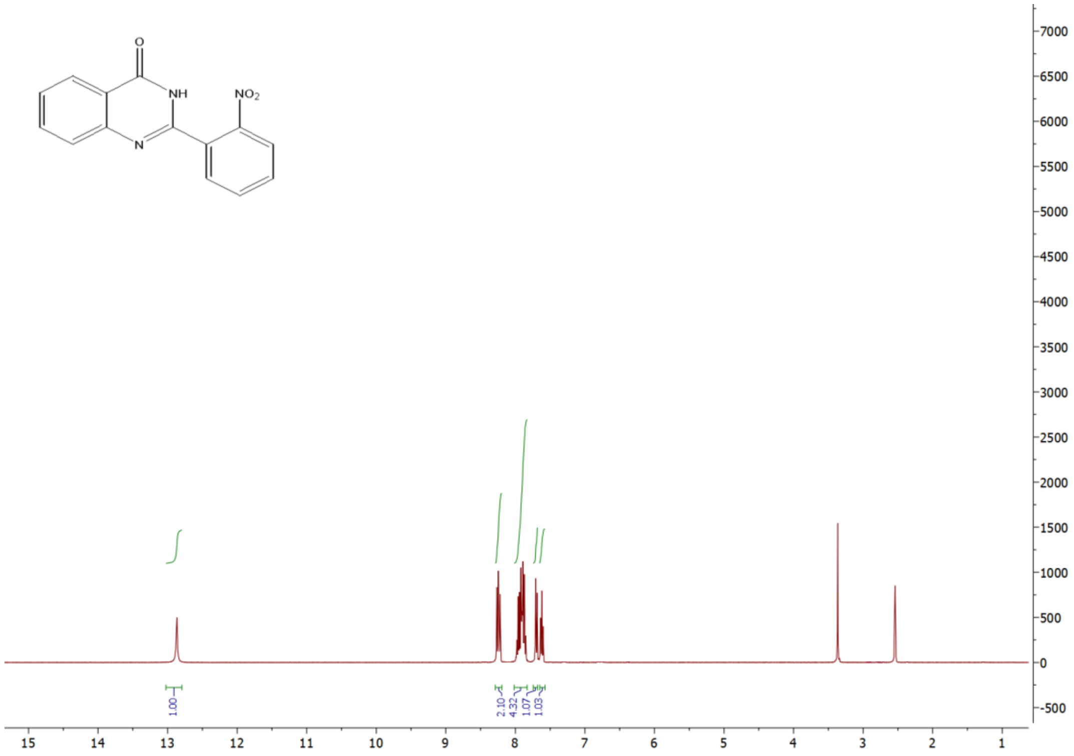

Figure S07: ^{1}H NMR of 3(**g**)

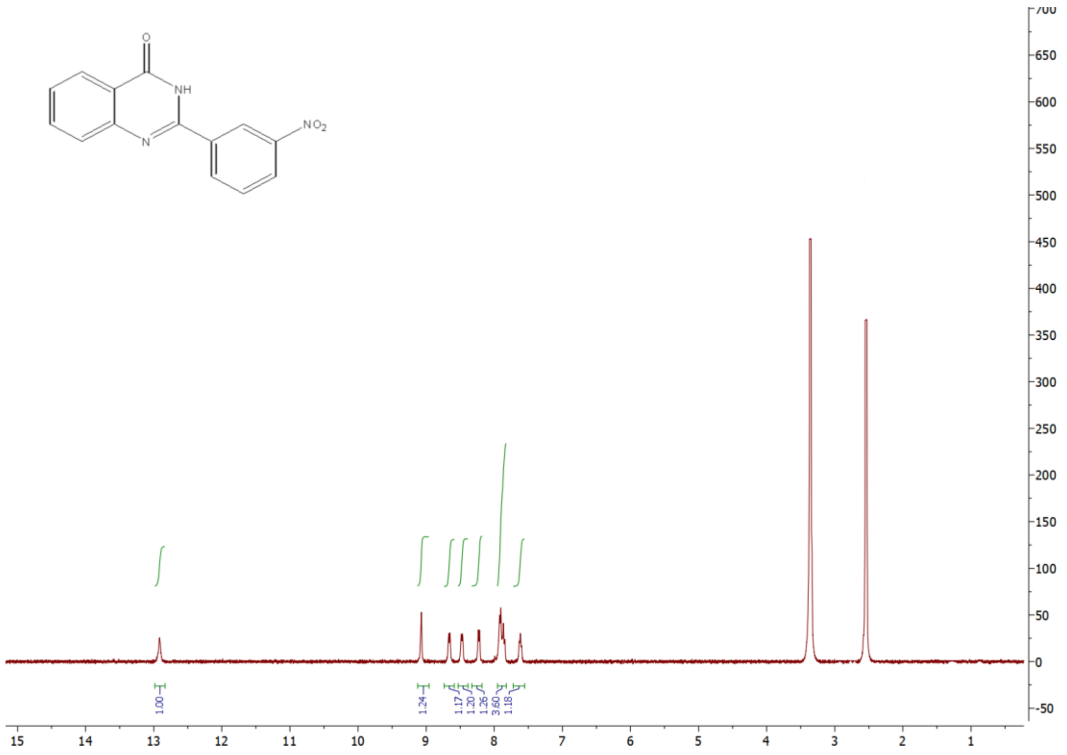

Figure S08: ^{1}H NMR of 3(**h**)

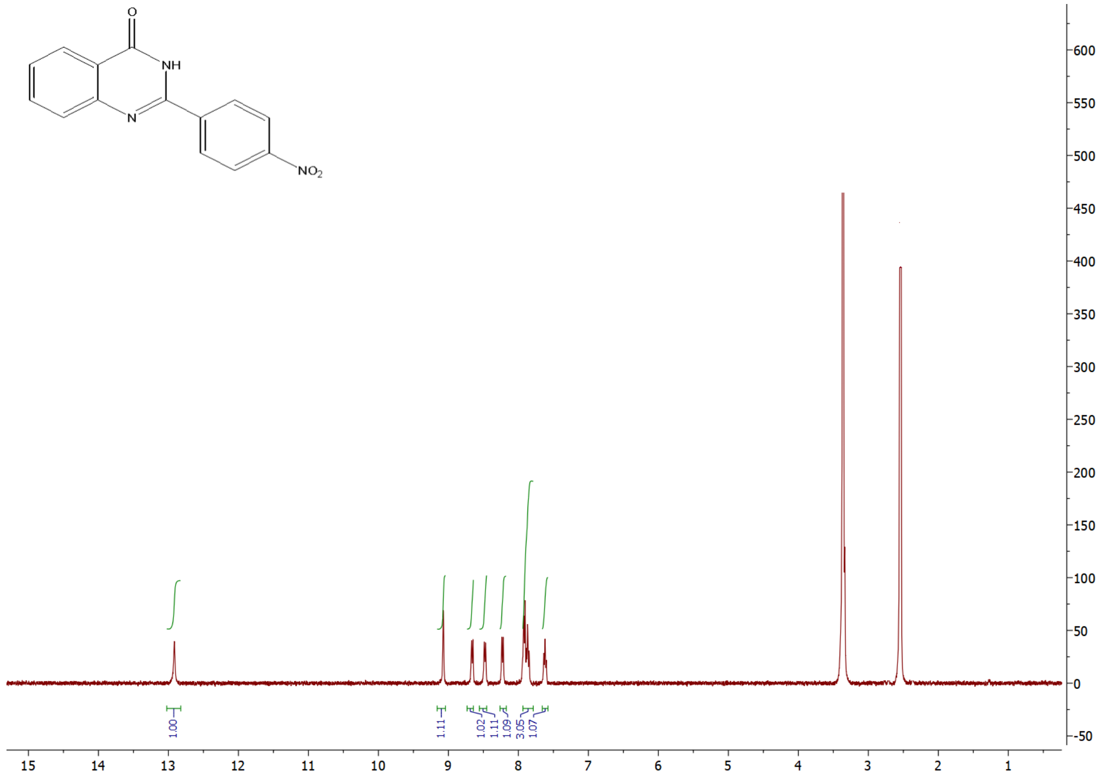

Figure S09: ^{1}H NMR of 3(**i**)

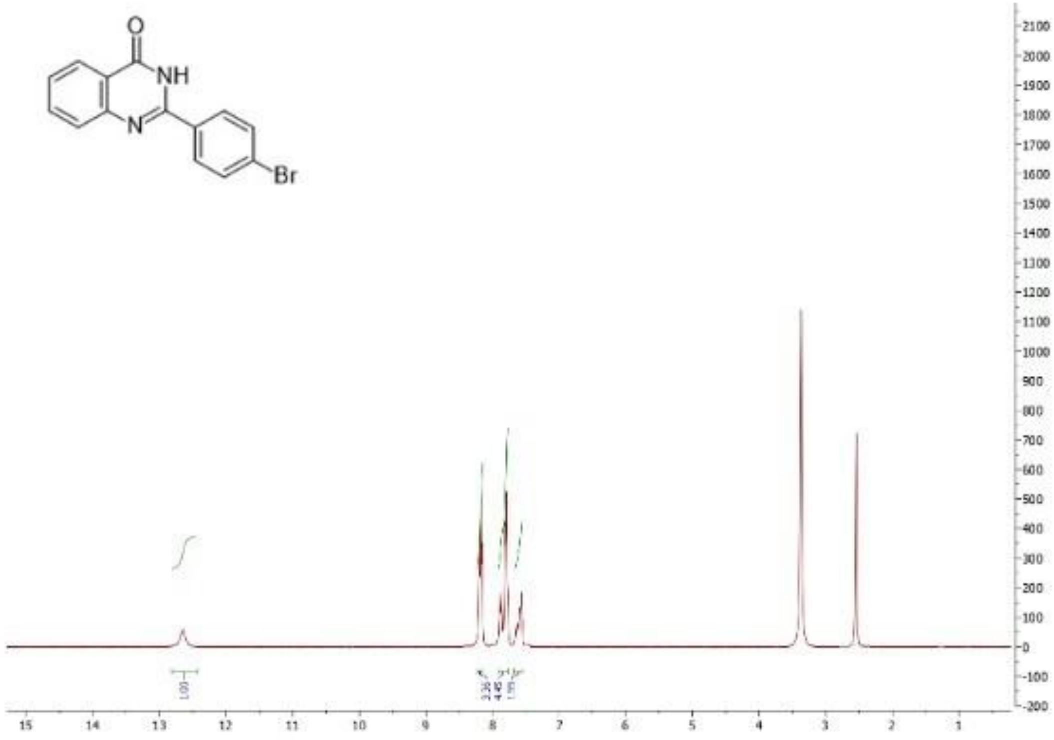

Figure S10: ^{1}H NMR of 3(**j**)

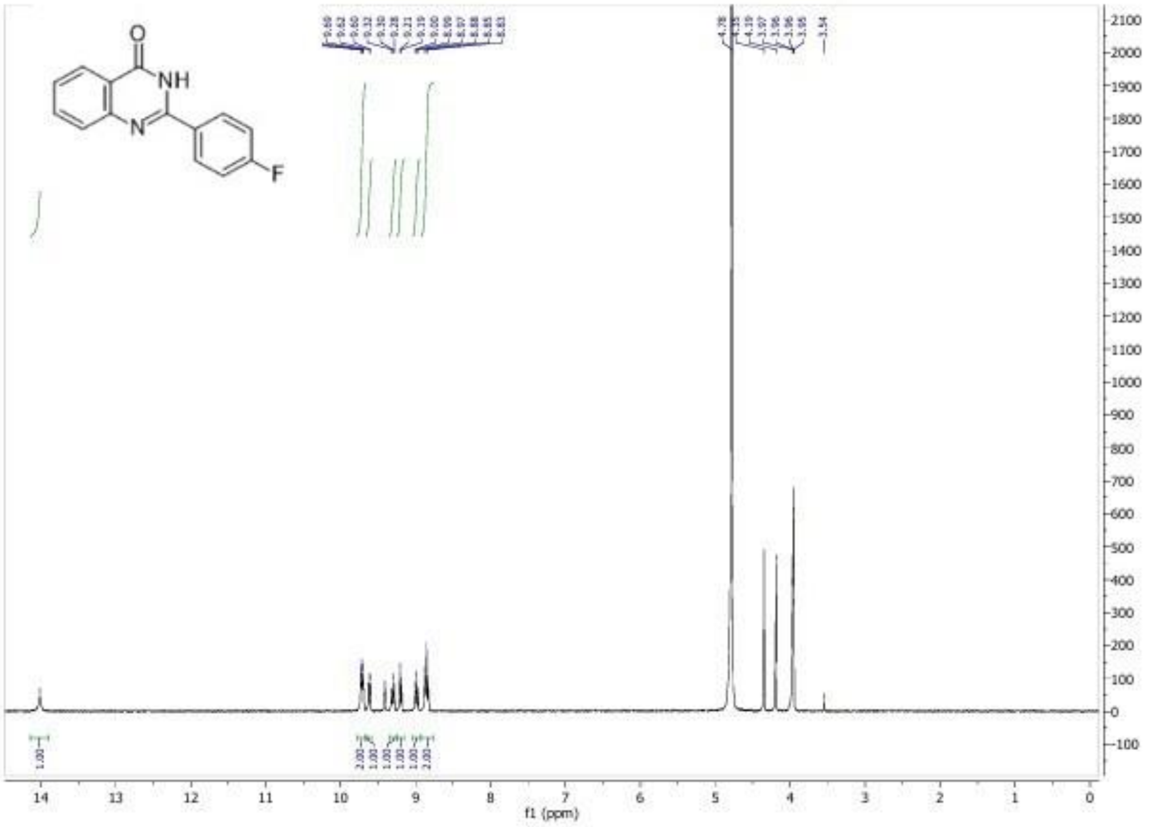

Figure S11: ¹H NMR of 3(**k**)

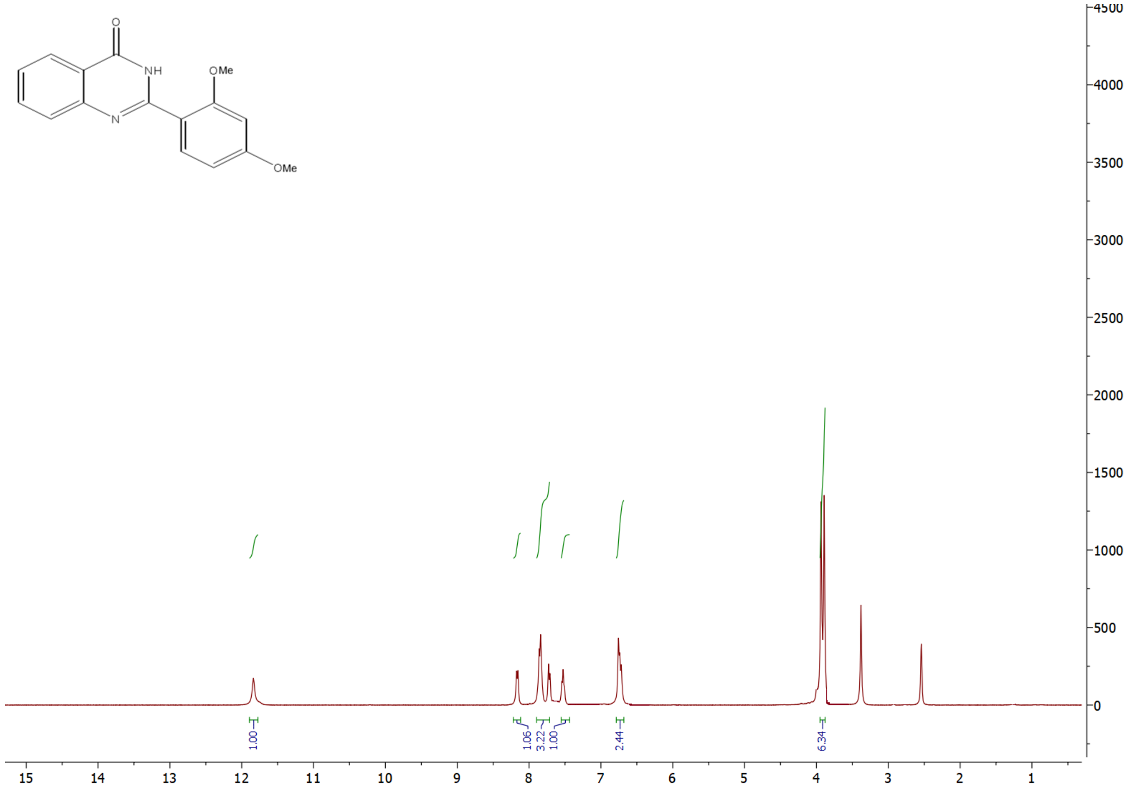

Figure S12: ¹H NMR of 3(**l**)

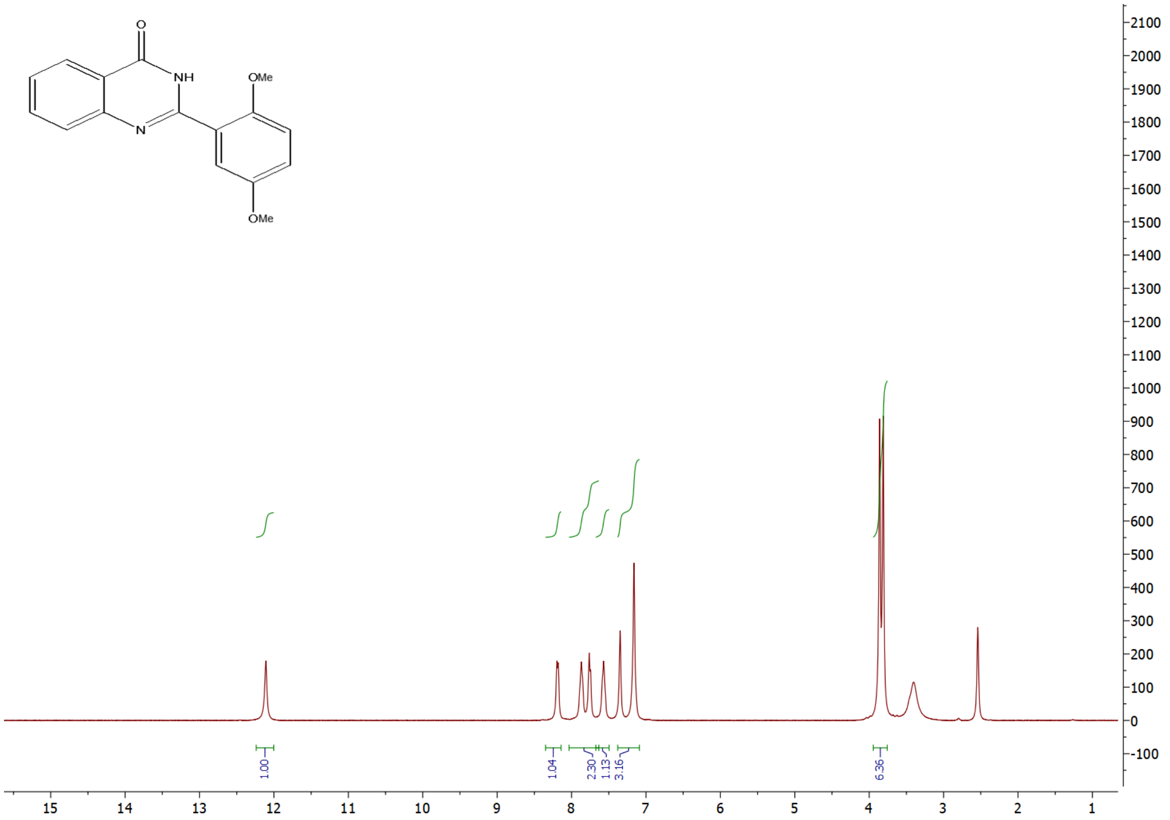

Figure S13: ^{1}H NMR of 3(**m**)

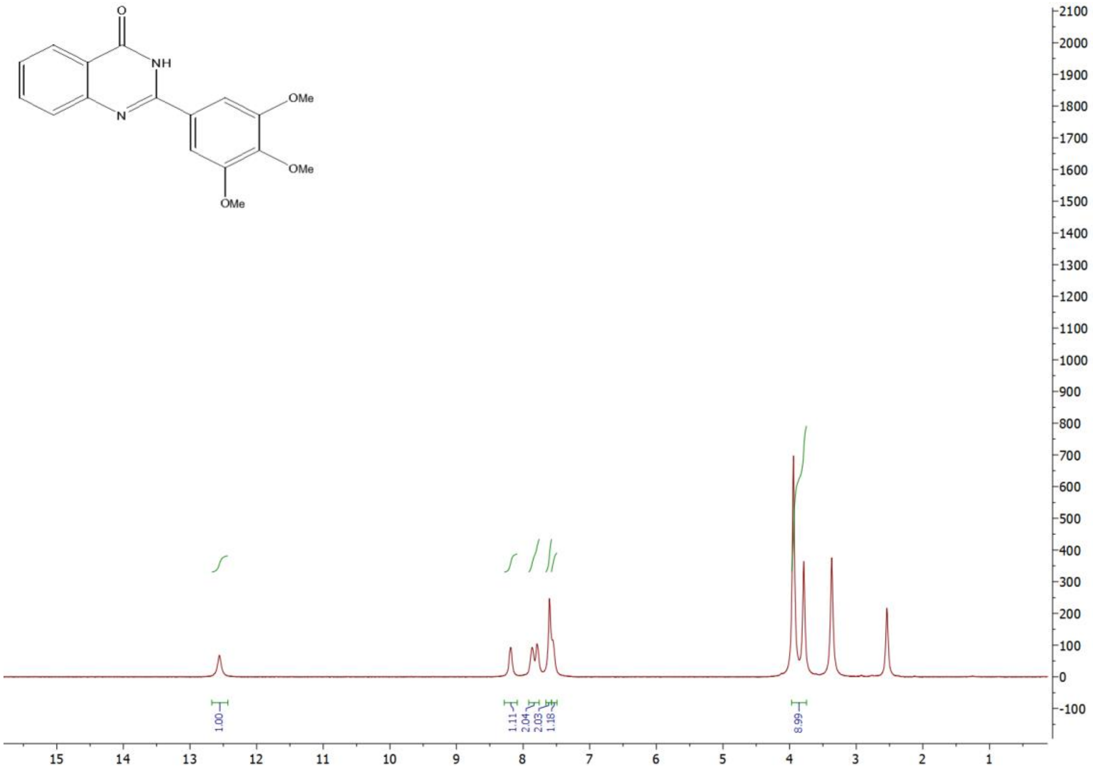

Figure S14: ^{1}H NMR of 3(**n**)

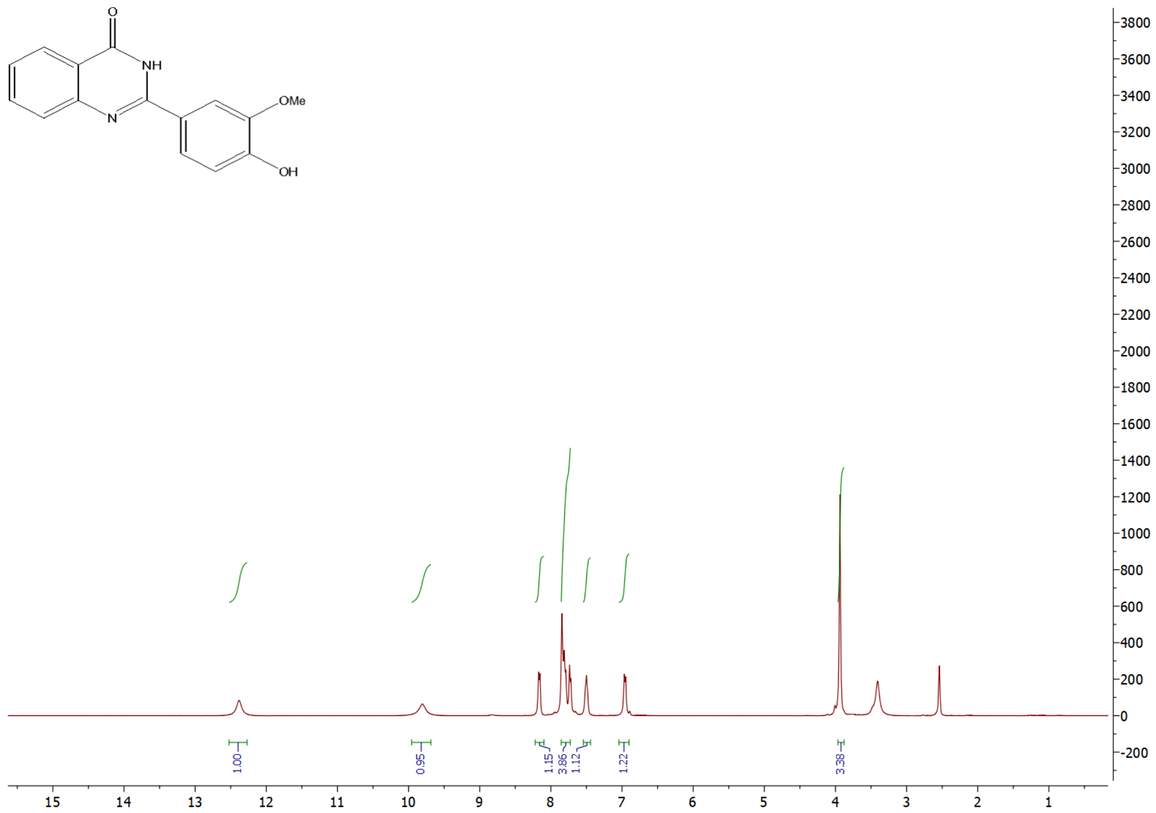

Figure S15: ^{1}H NMR of 3(**o**)

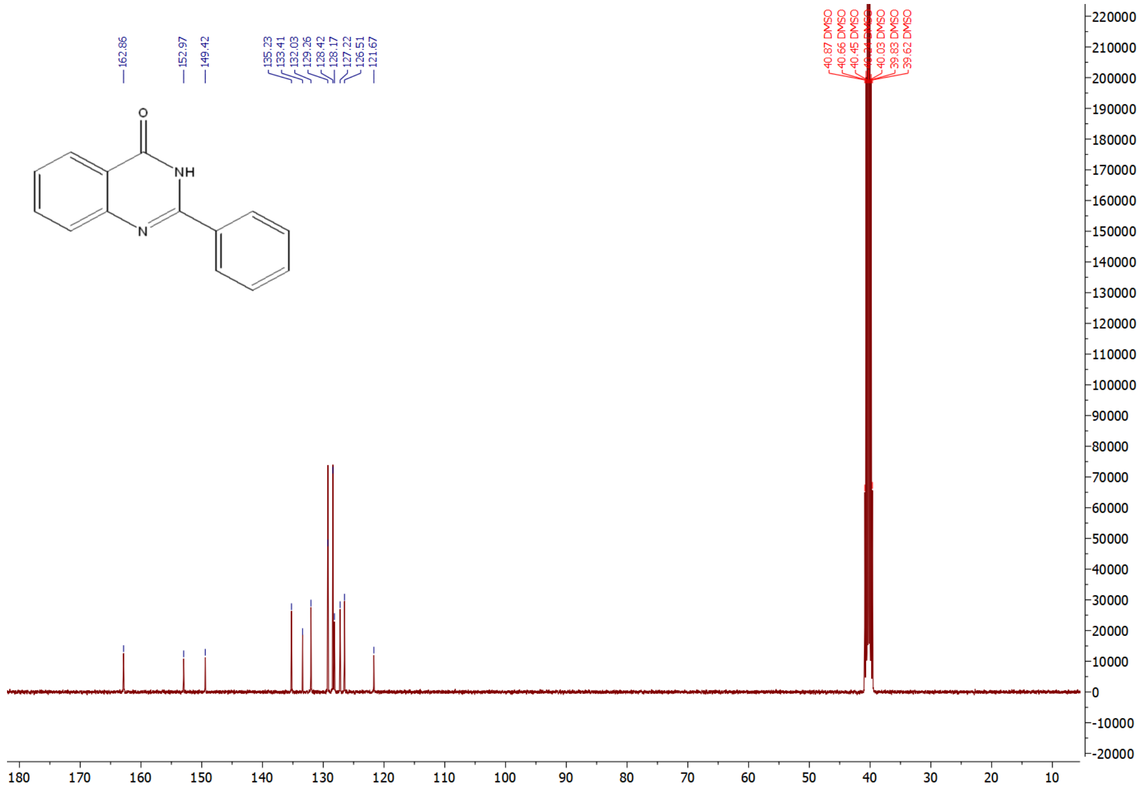

Figure S16: ^{13}C NMR of 3(**a**)

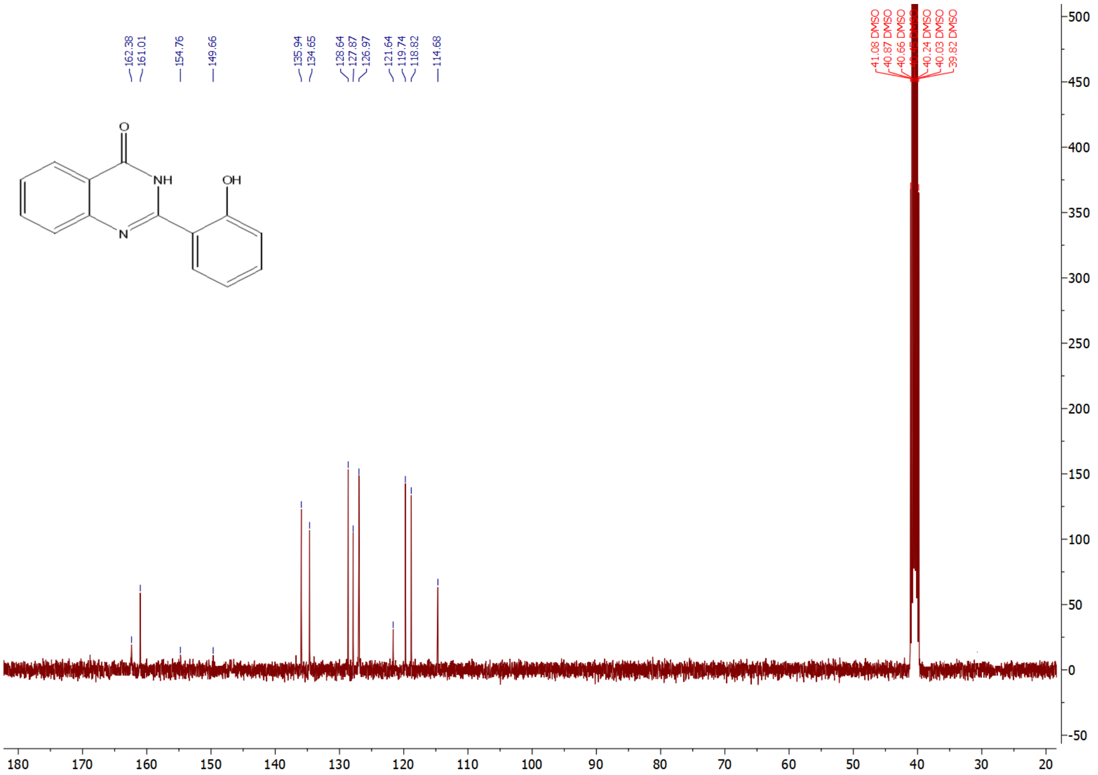

Figure S17: ^{13}C NMR of 3(**b**)

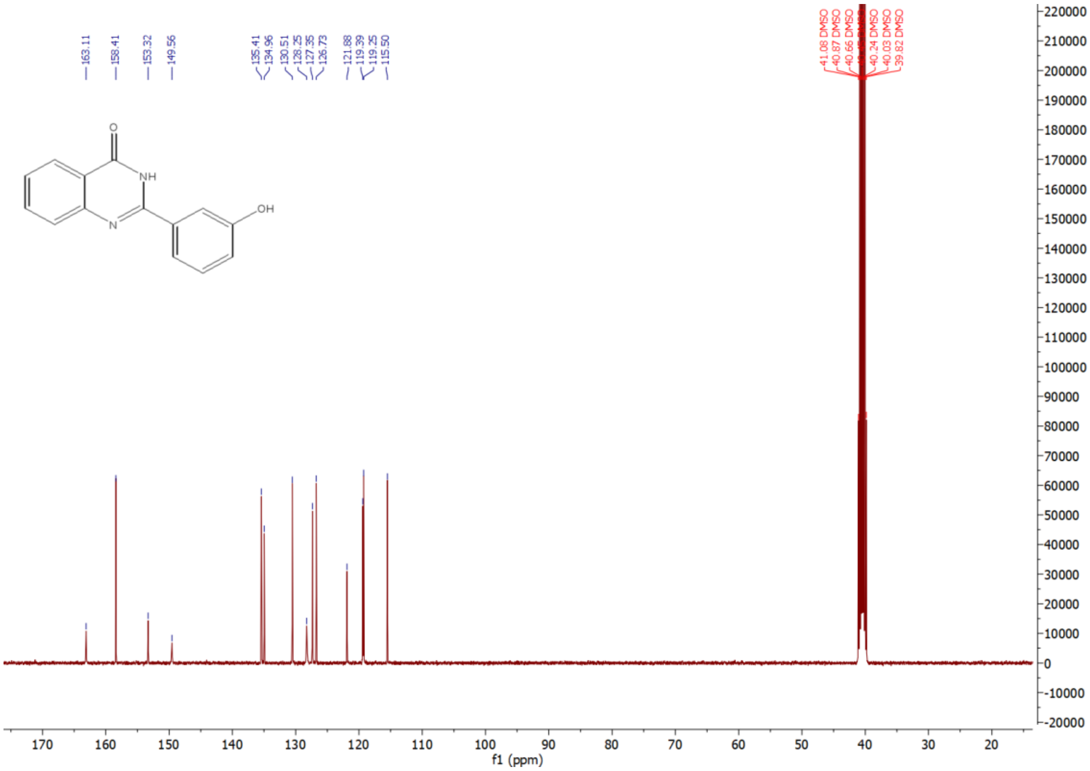

Figure S18: ^{13}C NMR of 3(**c**)

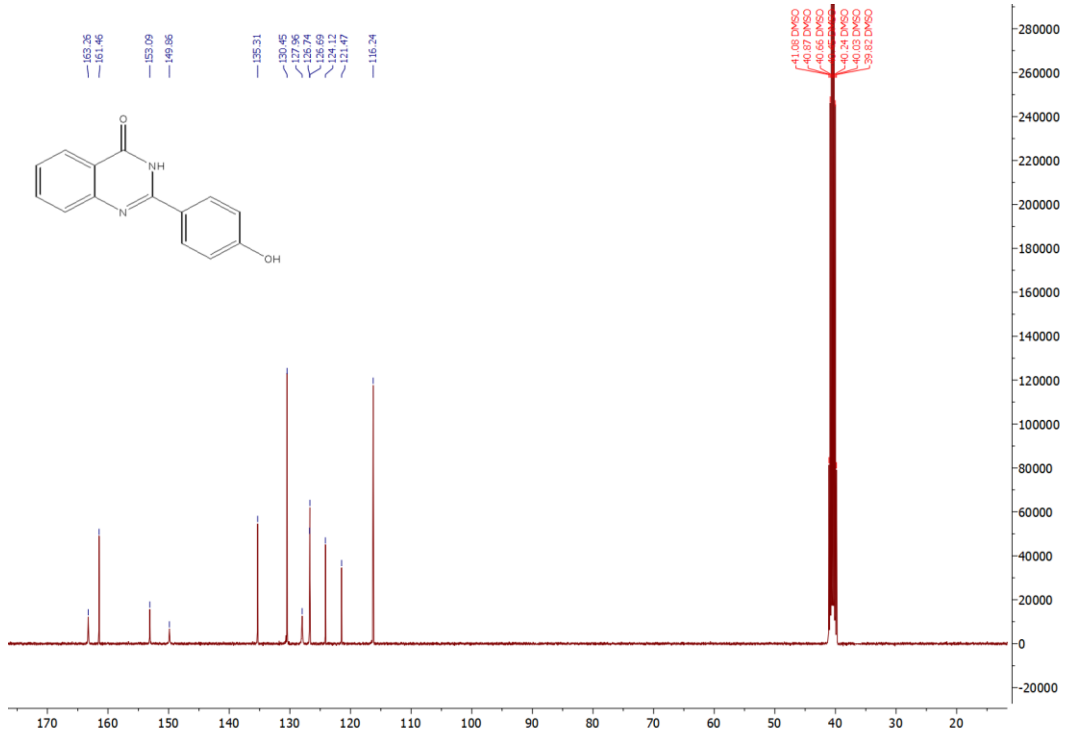

Figure S19: ^{13}C NMR of 3(**d**)

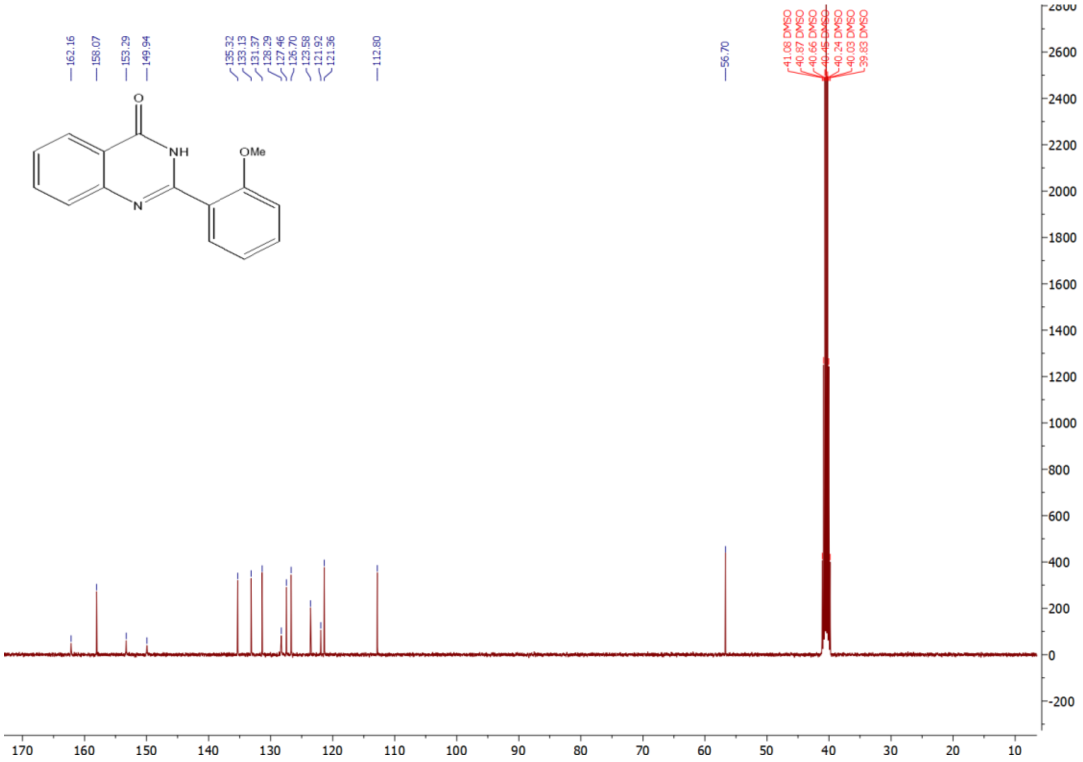

Figure S20: ^{13}C NMR of 3(**e**)

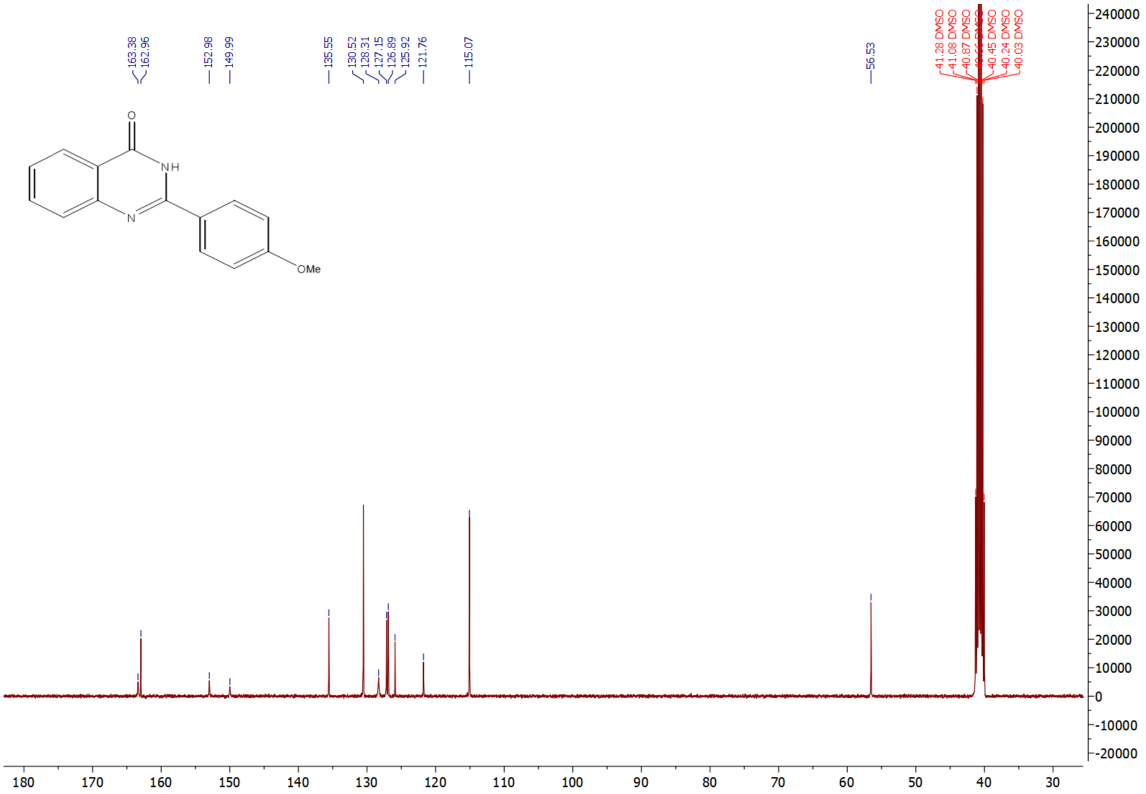

Figure S21: ^{13}C NMR of 3(**f**)

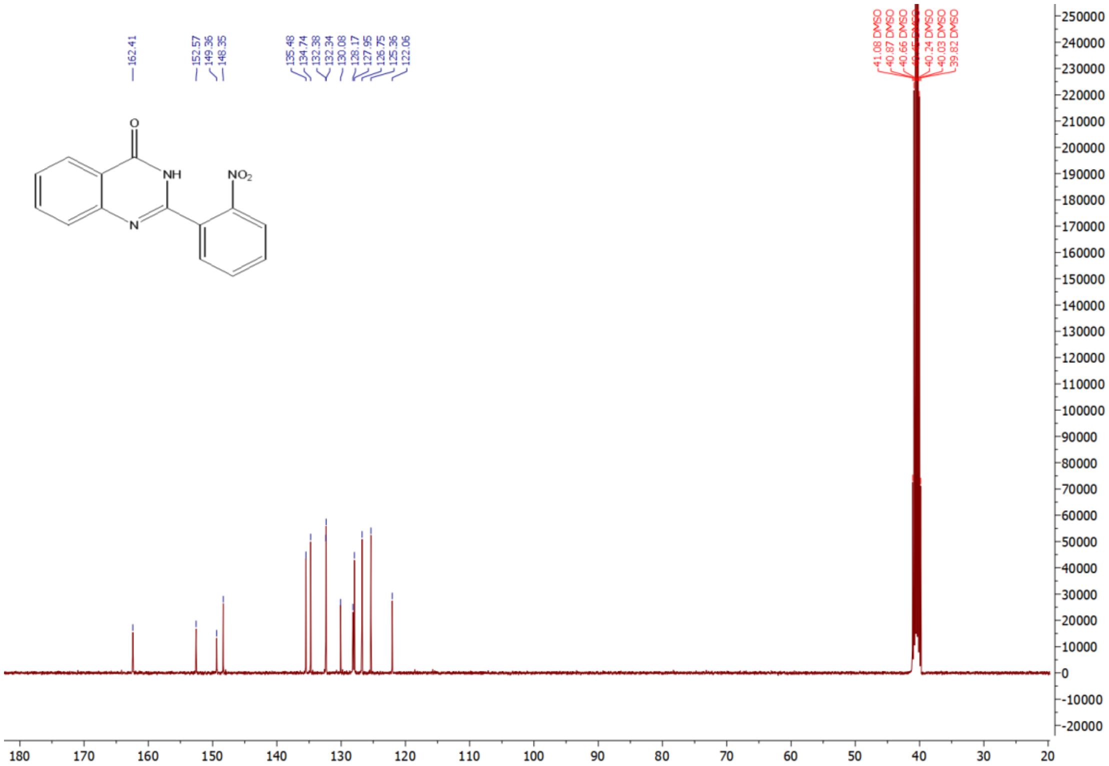

Figure S22: ^{13}C NMR of 3(**g**)

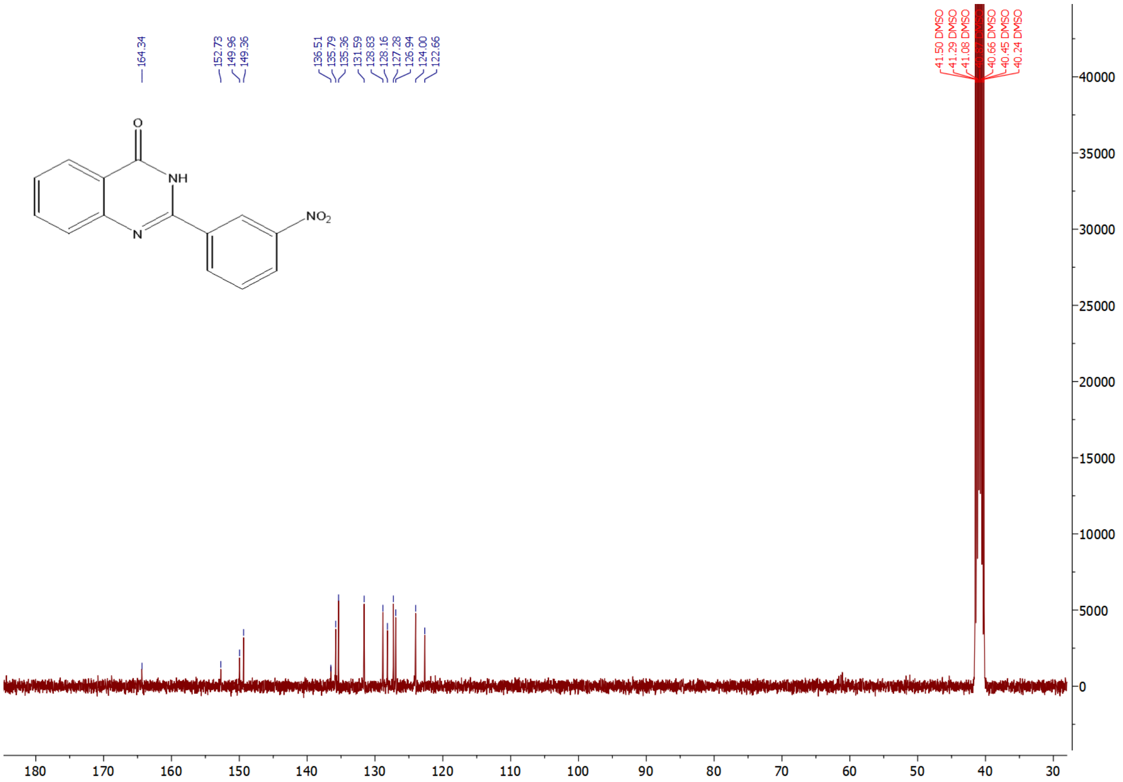

Figure S23: ^{13}C NMR of 3(**h**)

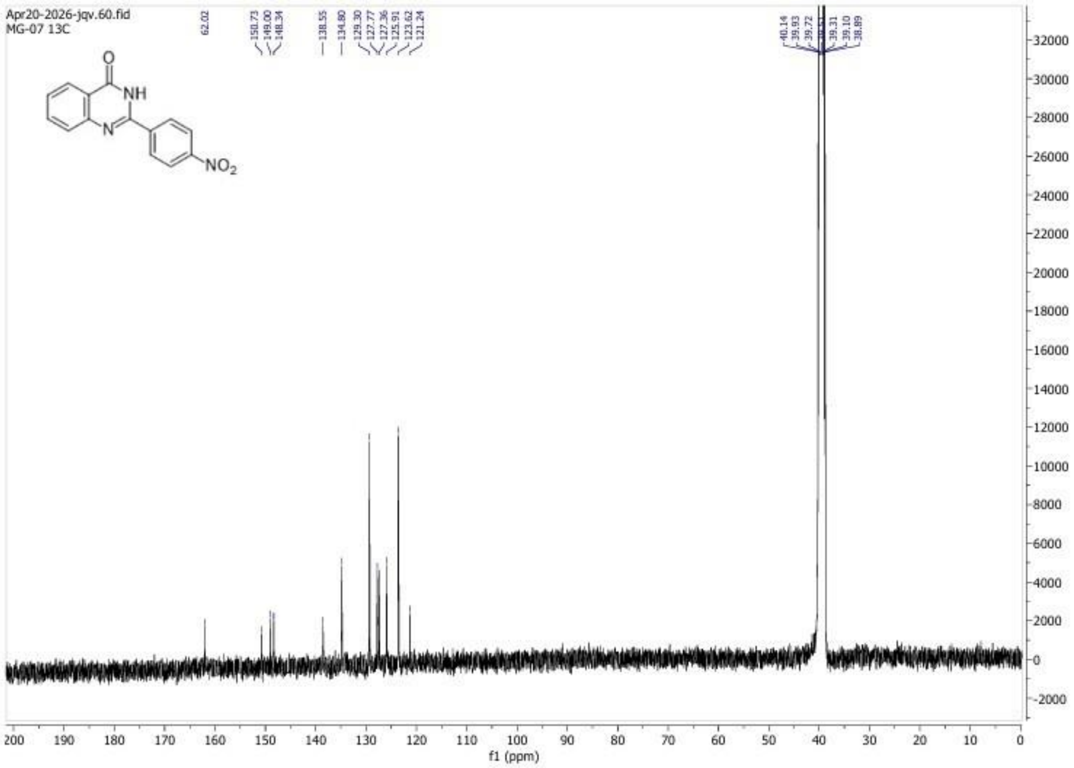

Figure S24: ^{13}C NMR of 3(**i**)

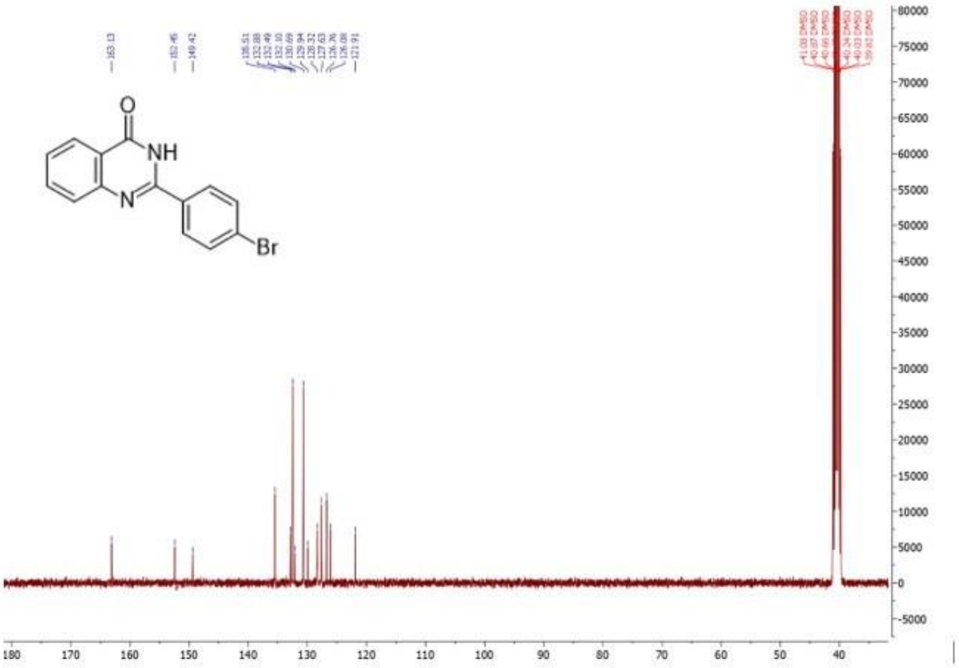

Figure S25: ^{13}C NMR of 3(**j**)

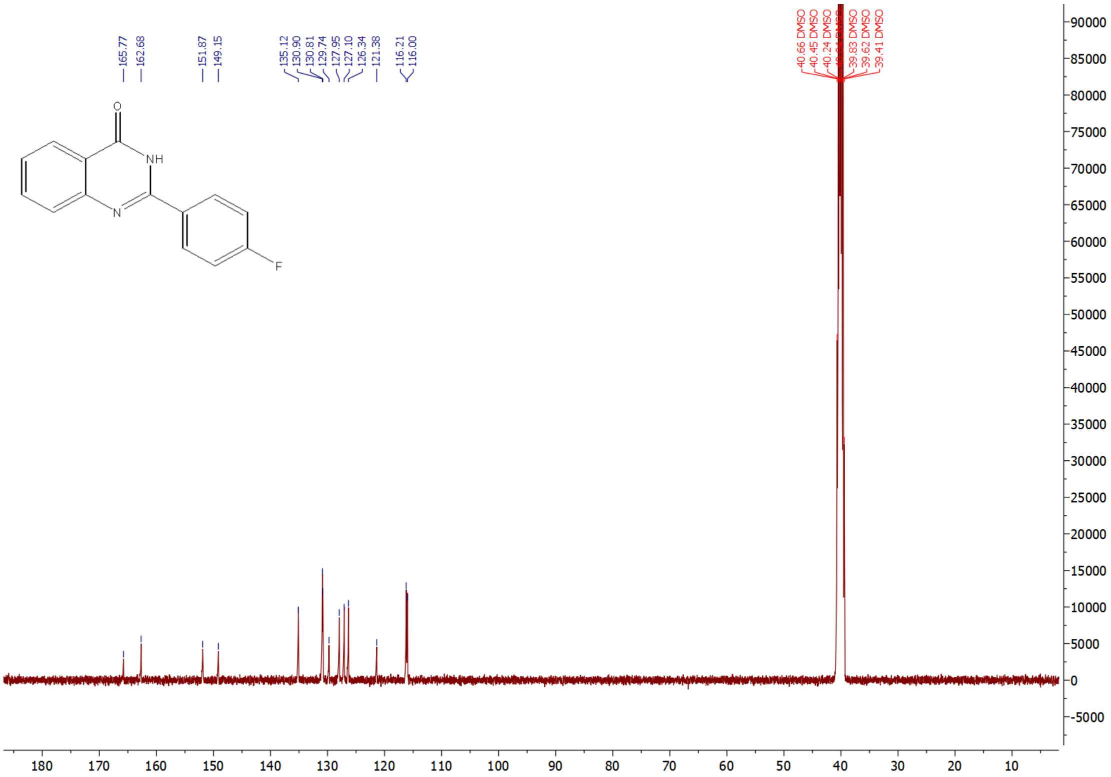

Figure S26: ^{13}C NMR of 3(**k**)

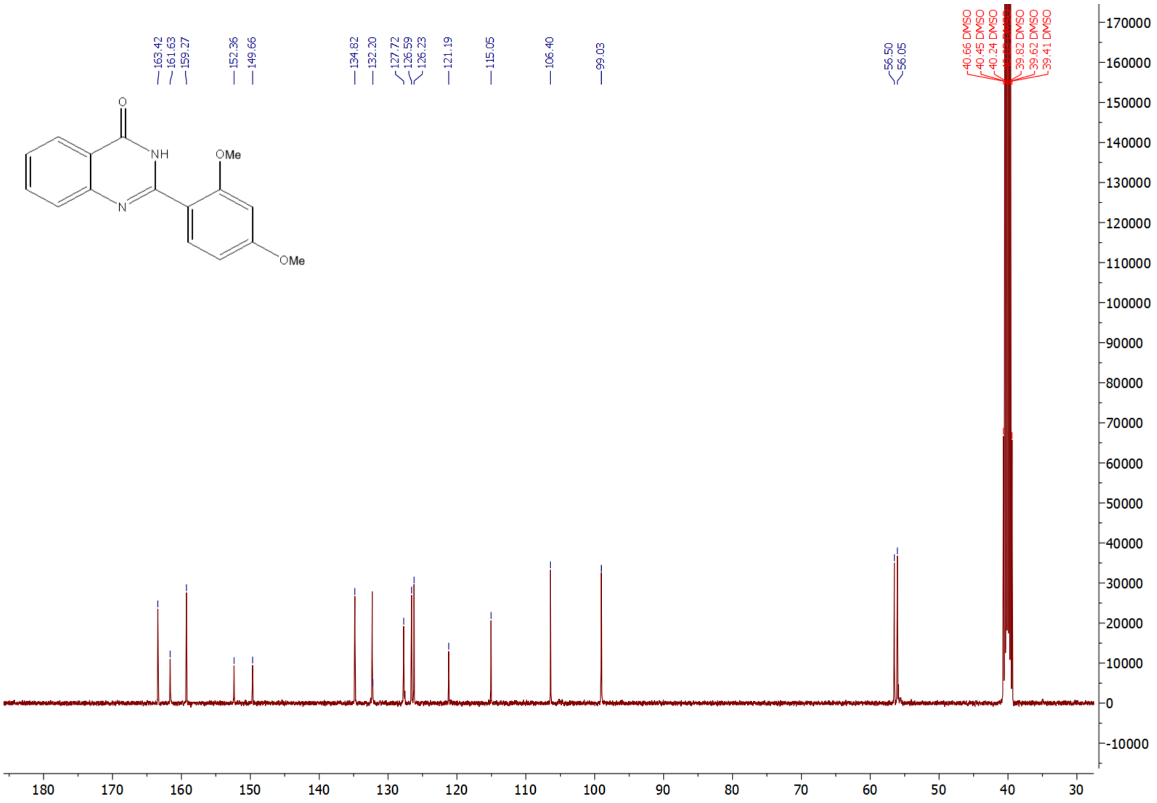

Figure S27: ^{13}C NMR of 3(**l**)

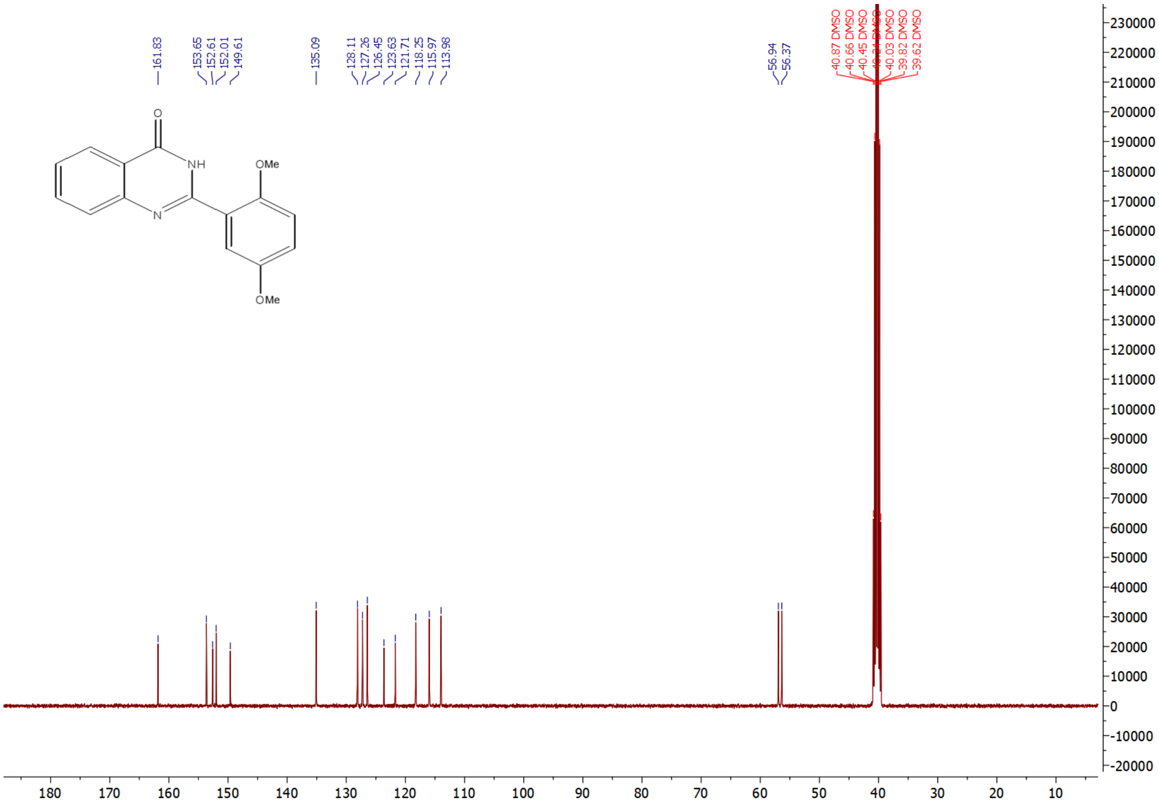

Figure S28: ^{13}C NMR of 3(**m**)

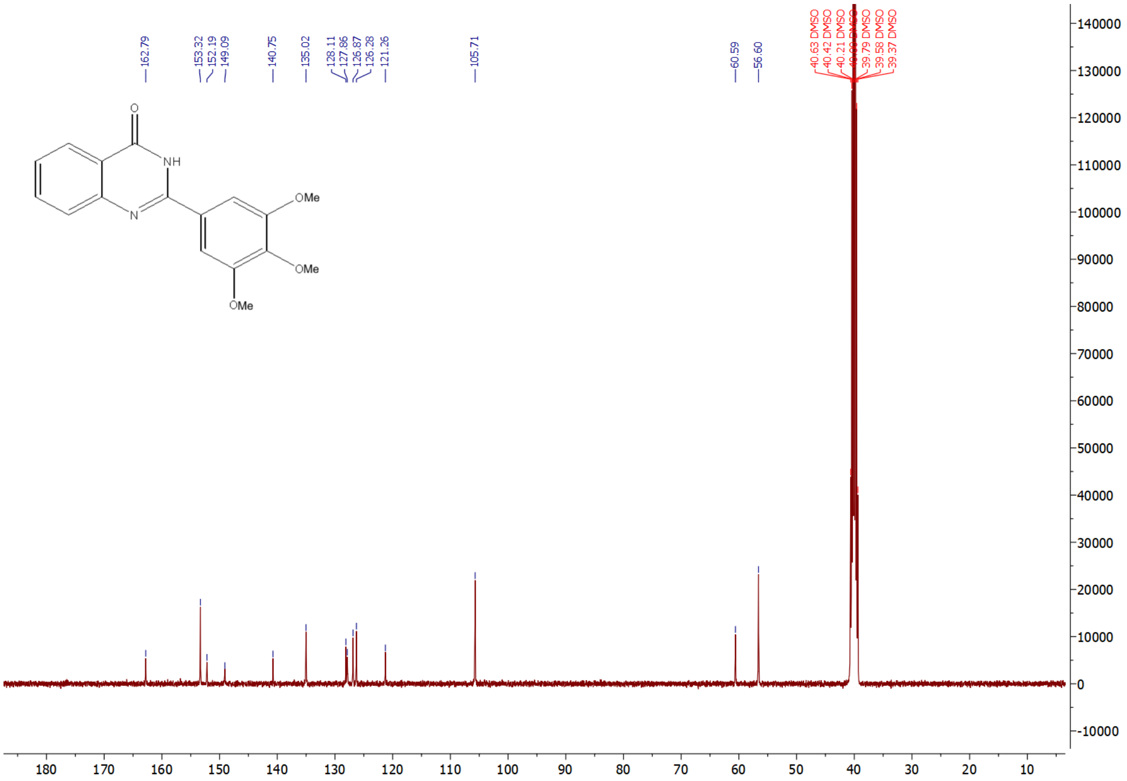

Figure S29: ^{13}C NMR of 3(**n**)

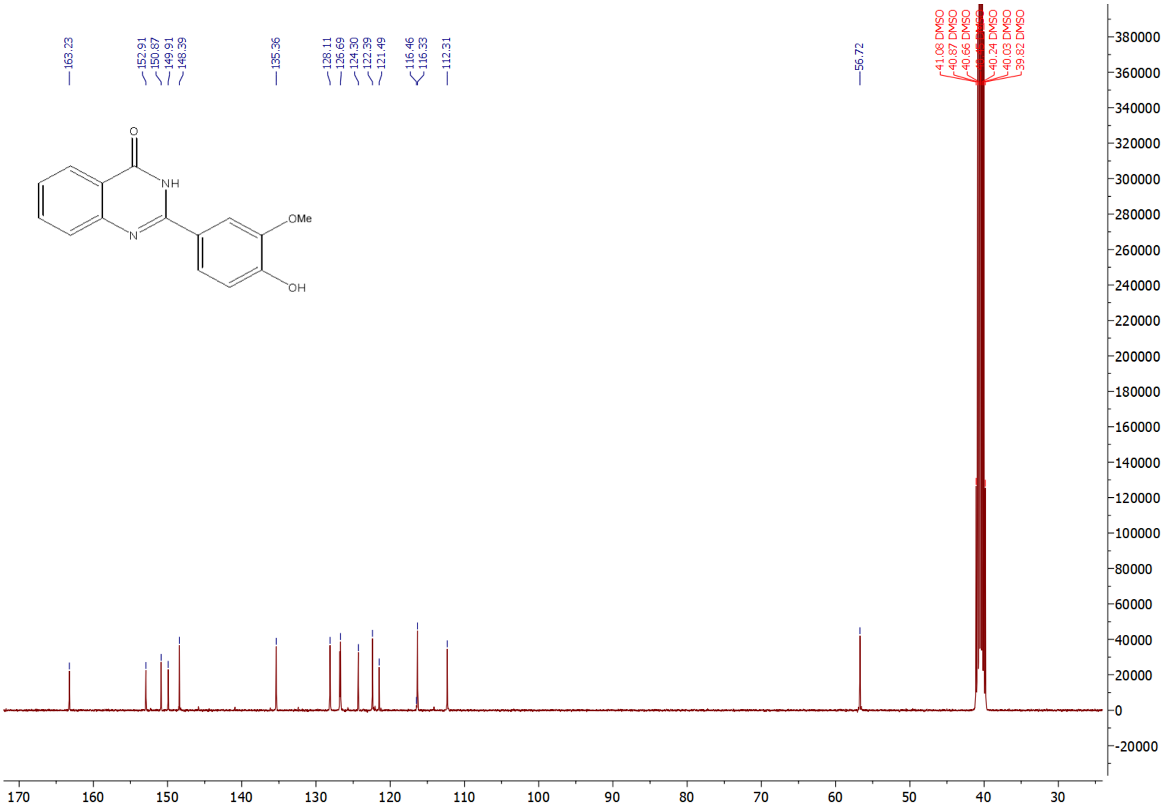

Figure S30: ^{13}C NMR of 3(**o**)

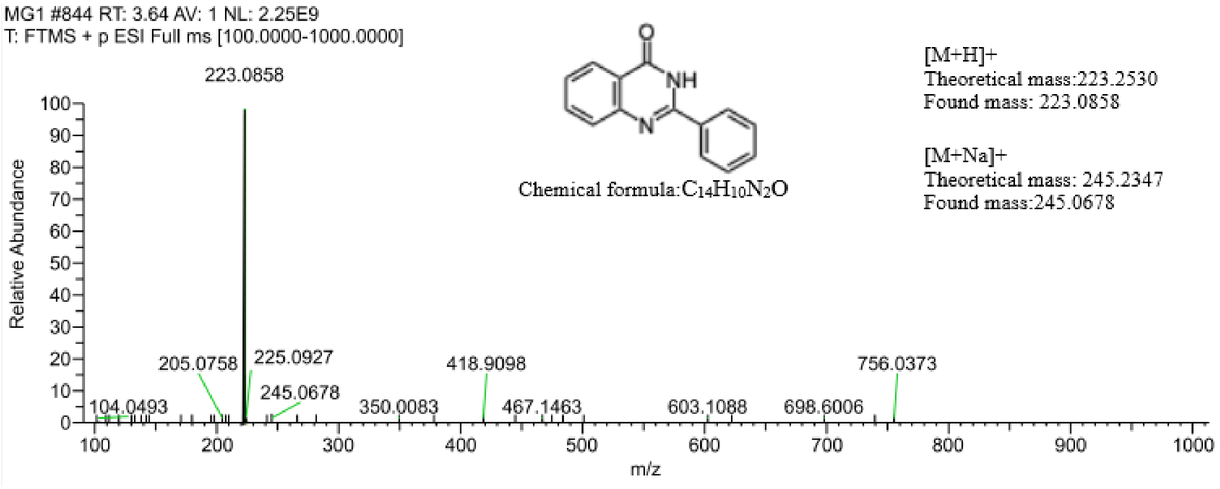

Figure S31: Mass spectrum of 3(**a**)

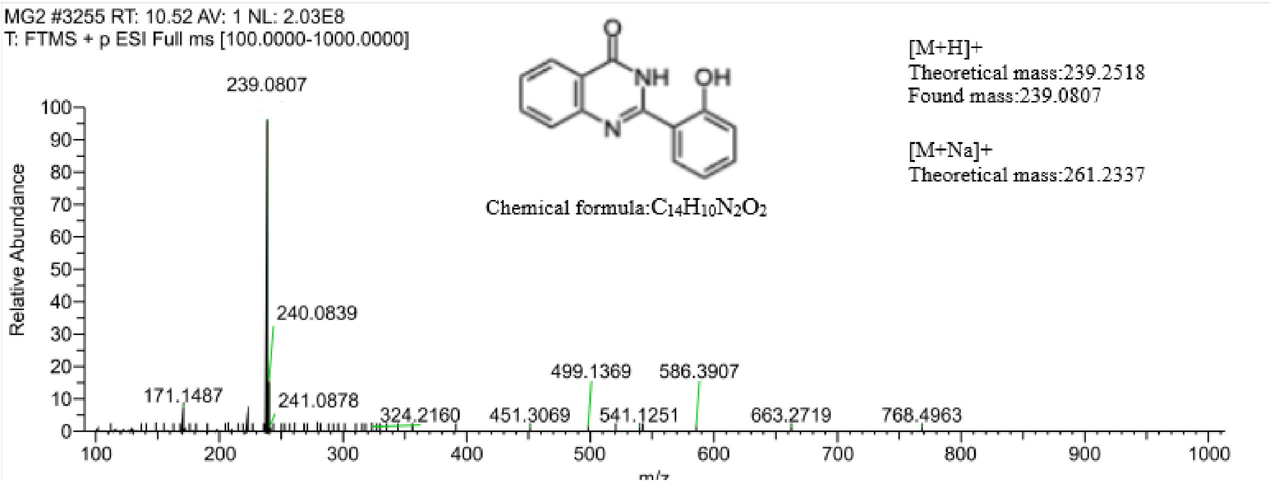

Figure S32: Mass spectrum of 3(**b**)

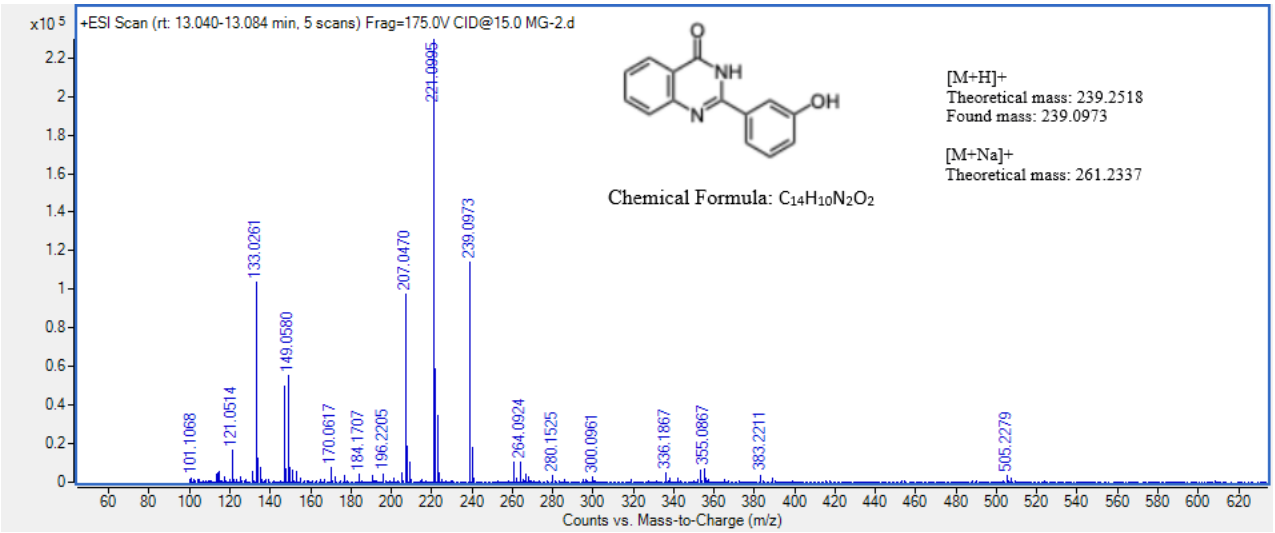

Figure S33: Mass spectrum of 3(**c**)

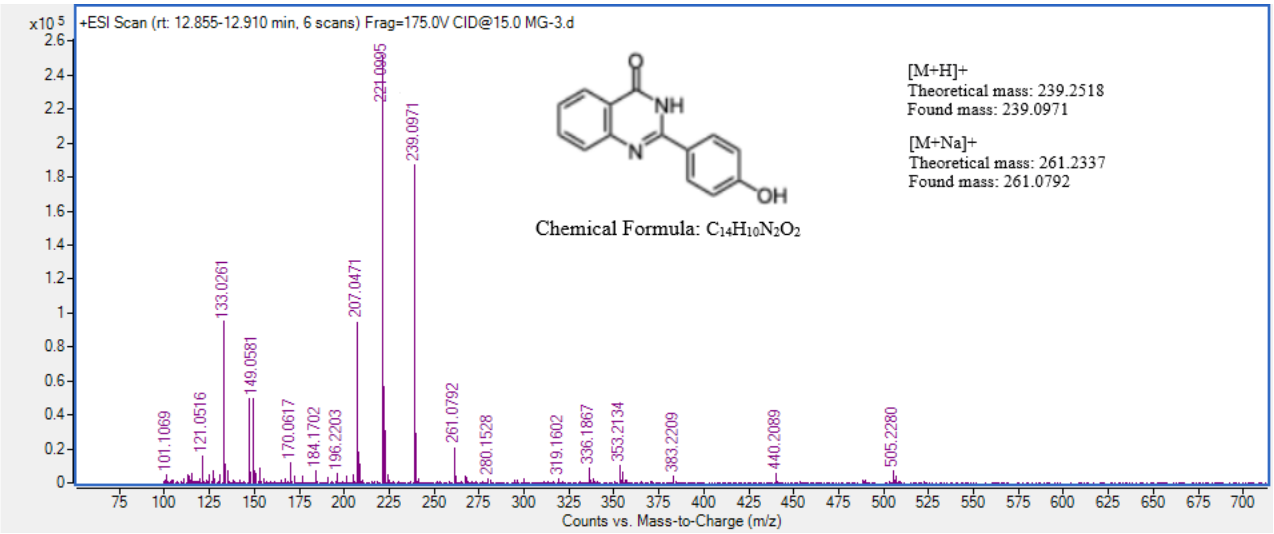

Figure S34: Mass spectrum of 3(**d**)

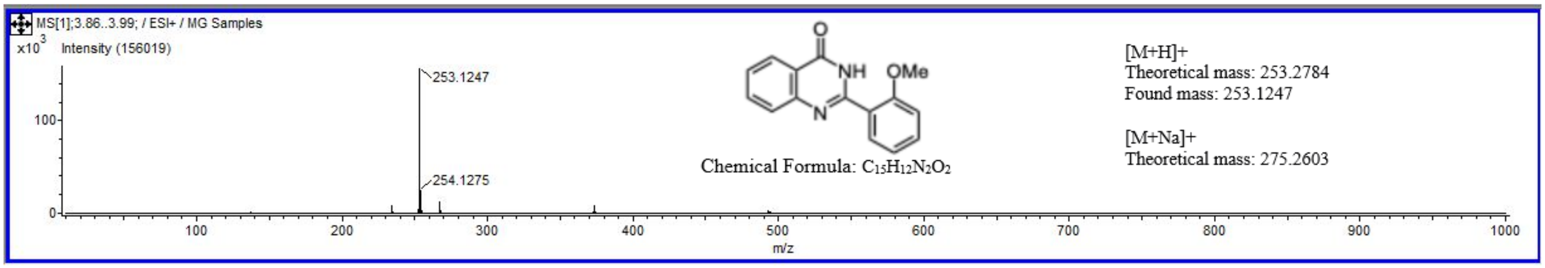

Figure S35: Mass spectrum of 3(**e**)

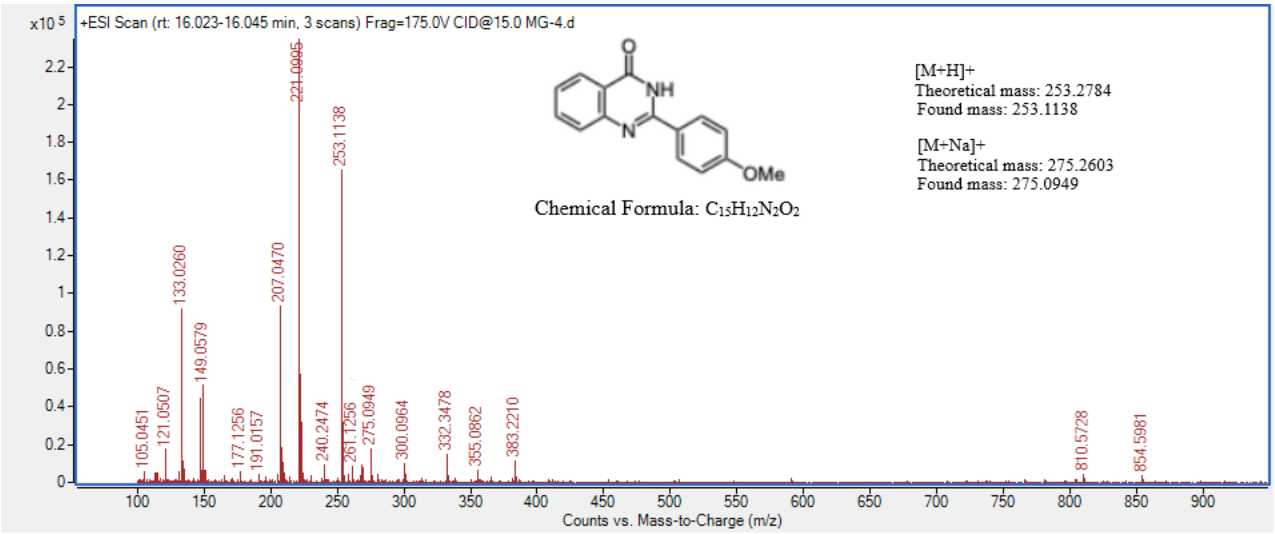

Figure S36: Mass spectrum of 3(**f**)

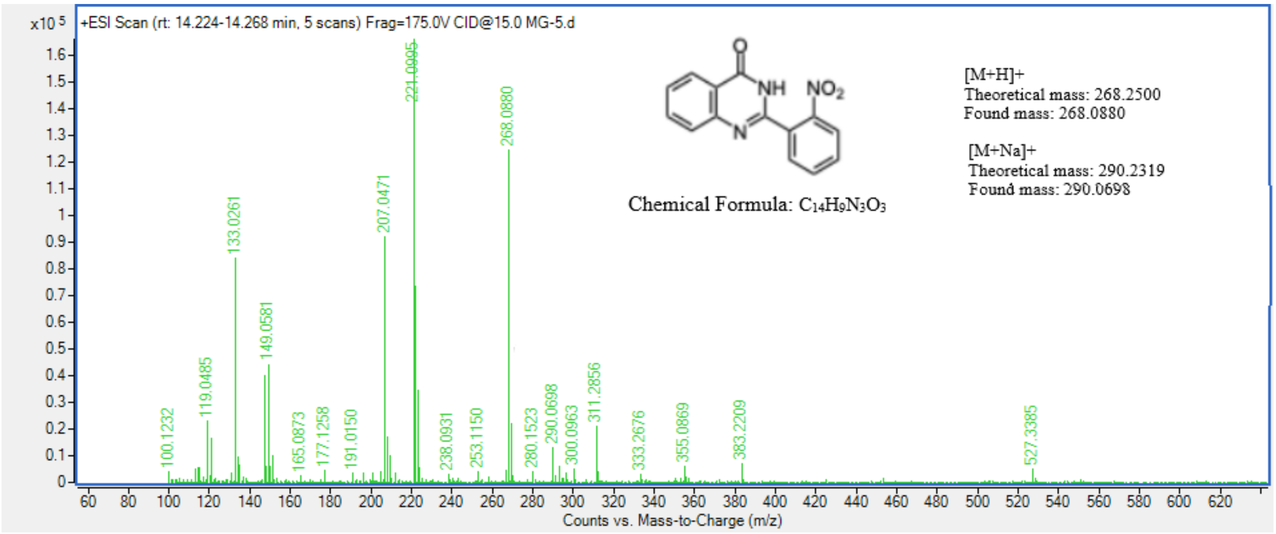

Figure S37: Mass spectrum of 3(**g**)

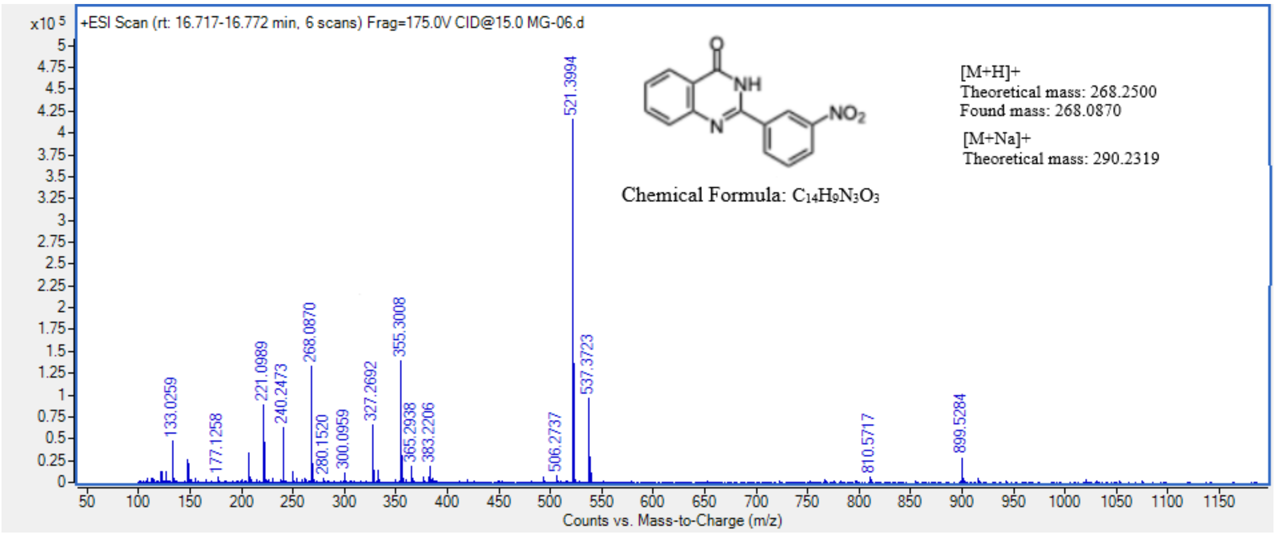

Figure S38: Mass spectrum of 3(**h**)

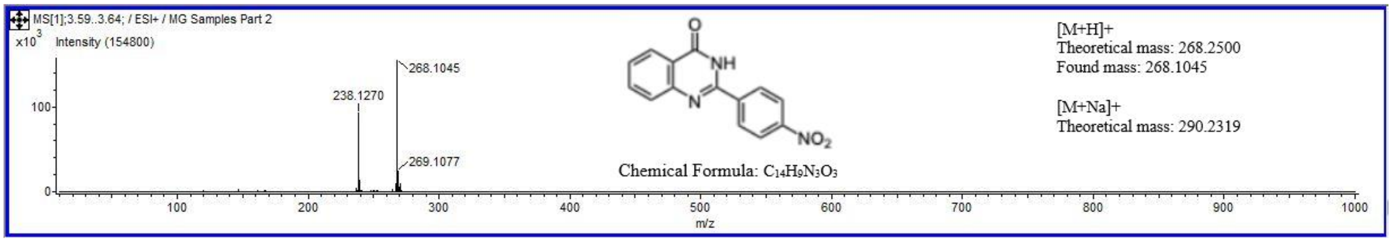

Figure 39: Mass spectrum of 3(**i**)

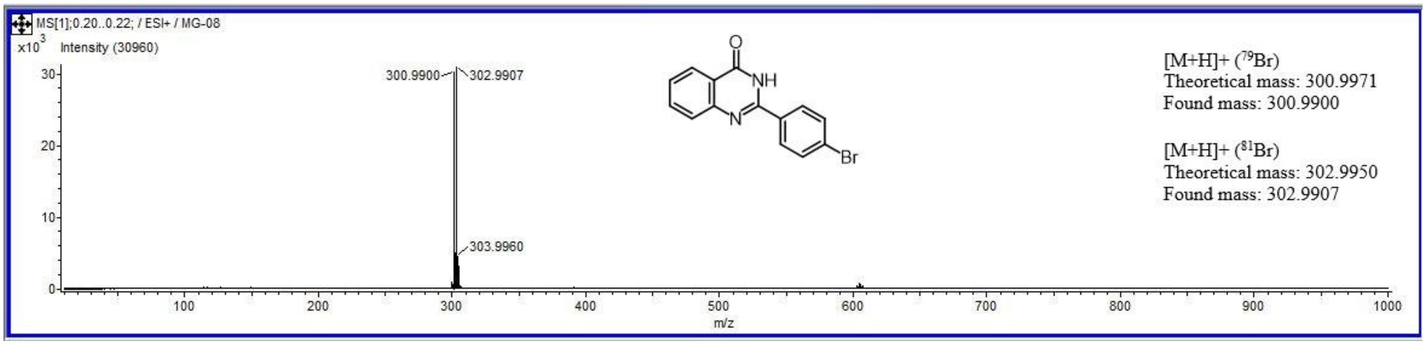

Figure S40: Mass spectrum of 3(**j**)

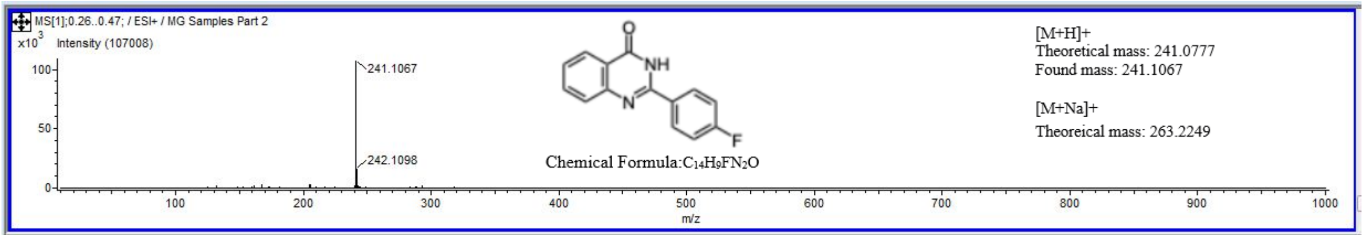

Figure S41: Mass spectrum of 3(**k**)

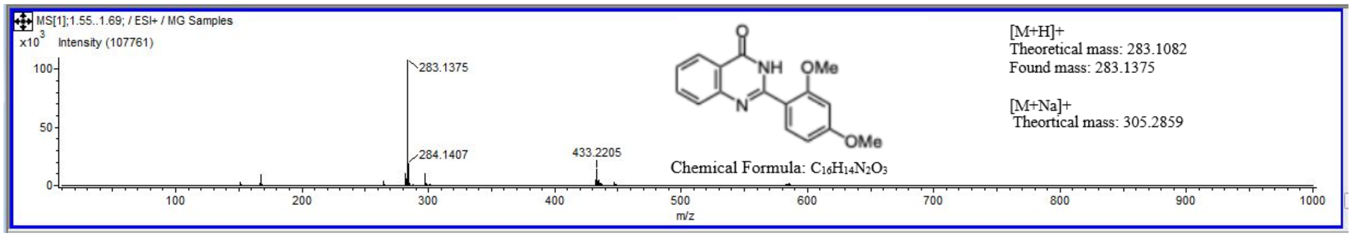

Figure S42: Mass spectrum of 3(**l**)

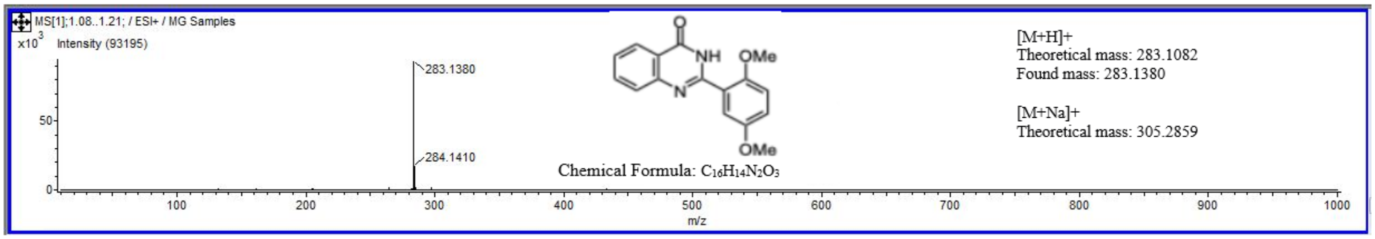

Figure S43: Mass spectrum of 3(**m**)

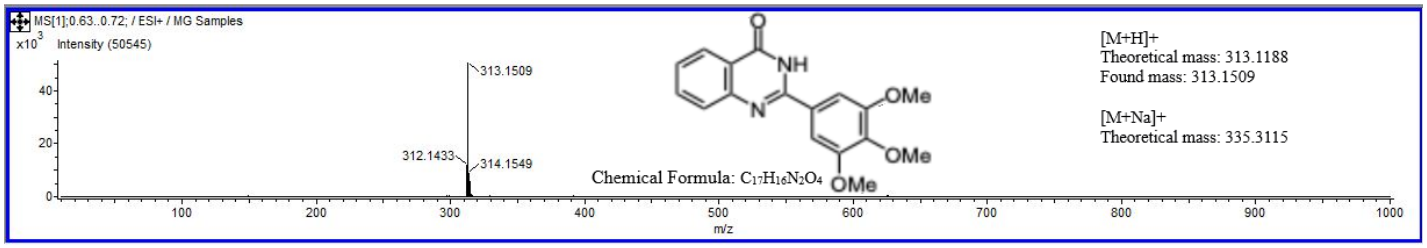

Figure S44: Mass spectrum of 3(**n**)

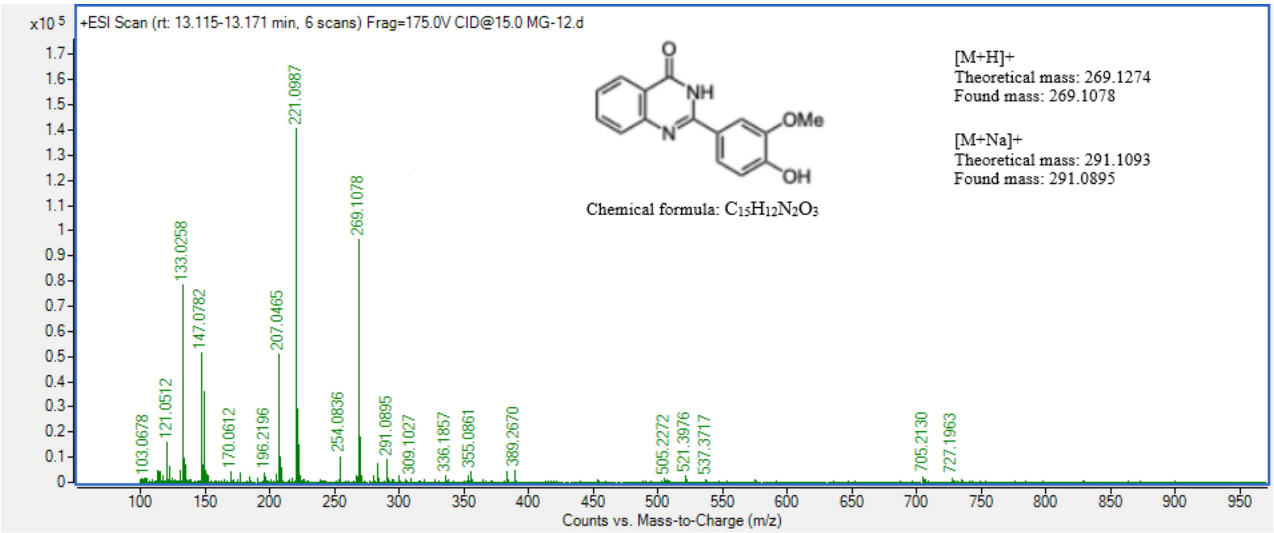

Figure S45: Mass spectrum of 3(**o**)
